# Supplementary material for: New mechanism to identify cost savings in English NHS prescribing: minimising ‘price per unit’, a cross-sectional study
Source: BMJ Open. 2018 Feb 8;8(2):e019643. doi: 10.1136/bmjopen-2017-019643 (PMC5829890; doi:10.1136/bmjopen-2017-019643)
Supplement: Supplementary file 1 [file bmjopen-2017-019643supp001.pdf]

The forma	Code	Sept_Quantity	Formulation	Alternative formulation	Really equivalent?	Alternative name	Alternative quantity	Number of words in formulation	Alternative code	DM+Dnonbioequivalent
Alum Hydrox	0101010C0AAAAAA	20973	Cap	Tab		Alum Hydrox	#N/A	1	0101010C0AAADAD	TRUE
Mag Carb_H	0101010F0AAAUAU	3217	Heavy Cap	Cap		Mag Carb_C	#N/A	2	0101010F0AAAIAI	TRUE
Co-Magaldro	0101010G0AAABAB	1556288	Susp	Liq		Co-Magaldro	#N/A	1	0101010G0AAAFAF	TRUE
Mag Ox_Cap	0101010I0AAABAB	6850	Cap	Tab	Y	Mag Ox_Tab	0101010I0AA	1	0101010I0AAAEAE	TRUE
Mag Ox_Cap	0101010I0AAACAC	17542	Cap	Tab	Y	Mag Ox_Tab	0101010I0AA	1	0101010I0AAALAL	TRUE
Mag Ox_Tab	0101010I0AAAEAE	1166	Tab	Cap	Y	Mag Ox_Cap	0101010I0AA	1	0101010I0AAABAB	TRUE
Mag Ox_Cap	0101010I0AAAHAH	56	Cap	Tab	Y	Mag Ox_Tab	0101010I0AA	1	0101010I0AABIBI	TRUE
Mag Ox_Tab	0101010I0AAALAL	120	Tab	Cap	Y	Mag Ox_Cap	0101010I0AA	1	0101010I0AAACAC	TRUE
Mag Ox_Tab	0101010I0AAAXAX	424	Tab	Cap	Y	Mag Ox_Cap	0101010I0AA	1	0101010I0AAAYAY	TRUE
Mag Ox_Cap	0101010I0AAAYAY	836	Cap	Tab	Y	Mag Ox_Tab	0101010I0AA	1	0101010I0AAAXAX	TRUE
Mag Ox_Tab	0101010I0AABIBI	2259	Tab	Cap	Y	Mag Ox_Cap	0101010I0AA	1	0101010I0AAAHAH	TRUE
Simeticone_	0101010R0AAADAD	5650	Dps	Conc Dps		Simeticone_	#N/A	1	0101010R0AAAFAF	TRUE
Simeticone_	0101010R0AAAEAE	22185	Tab Chble	Cap	N	Simeticone_	0101010R0AA	2	0101010R0AAAHAH	TRUE
Simeticone_	0101010R0AAAHAH	21670	Cap	Tab Chble	N	Simeticone_	0101010R0AA	1	0101010R0AAAEAE	TRUE
Sod Bicarb_L	0101012B0AAAUAU	25400	Liq Spec	Oral Soln	Y	Sod Bicarb_C	0101012B0AA	2	0101012B0AABWBW	TRUE
Sod Bicarb_L	0101012B0AAAZAZ	1800	Liq Spec	Oral Soln		Sod Bicarb_C	#N/A	2	0101012B0AAAHAH	TRUE
Sod Bicarb_L	0101012B0AABSBS	4770	Liq Spec	Oral Soln	Y	Sod Bicarb_C	0101012B0AA	2	0101012B0AABVBV	TRUE
Sod Bicarb_C	0101012B0AABVBV	435	Oral Soln	Liq Spec	Y	Sod Bicarb_L	0101012B0AA	2	0101012B0AABSBS	TRUE
Sod Bicarb_C	0101012B0AABWBW	10780	Oral Soln	Liq Spec	Y	Sod Bicarb_L	0101012B0AA	2	0101012B0AAAUAU	TRUE
Calc Carb_Ta	0101021C0AAAFAF	6190	Tab Chble	Cap	N	Calc Carb_Ca	0101021C0AA	2	0101021C0AAAPAP	TRUE
Calc Carb_Ca	0101021C0AAAPAP	30	Cap	Tab		Calc Carb_Ta	#N/A	1	0101021C0AABTBT	TRUE
Calc Carb_Ta	0101021C0AAATAT	2340	Tab	Cap		Calc Carb_Ca	#N/A	1	0101021C0AAANAN	TRUE
Calc Carb_Ca	0101021C0AABQBQ	112	Cap	Gran Sach		Calc Carb_Gr	#N/A	1	0905011D0AAAMAM	TRUE
Calc Carb_Ta	0101021C0AABXBX	2536	Tab Chble	Tab	Y	Calc Carb_Ta	#N/A	2	0101021C0AACFCF	TRUE
Calc Carb_Lic	0101021C0AACECE	6460	Liq Spec	Susp		Calc Carb_Su	#N/A	2	0101021C0AABPBP	TRUE
Calc Carb_Di	0101021C0AACICI	6637	Disper Tab	Cap		Calc Carb_Ca	#N/A	2	0101021C0AABCBC	TRUE
Calc Carb_Lic	0101021C0AACUCU	250	Liq Spec	Susp		Calc Carb_Su	#N/A	2	0101021C0AABKBK	TRUE
Calc Carb_Or	0101021C0AACXCX	19200	Oral Susp	Liq Spec		Calc Carb_Lic	#N/A	2	0101021C0AACACA	TRUE
Calc Carb_Or	0101021C0AACYCY	7150	Oral Susp	Liq Spec		Calc Carb_Lic	#N/A	2	0101021C0AACBCB	TRUE
Atrop Sulf_Ta	0102000ACAAAGAG	1583	Tab	Cap		Atrop Sulf_Ca	#N/A	1	0102000ACAAALAL	TRUE
Glycopyrroni	0102000L0AAAWAW	240574	Oral Soln	Liq Spec		Glycopyrroni	#N/A	2	0102000L0AAADAD	TRUE
Glycopyrroni	0102000L0AAAXAX	71384	Oral Susp	Liq Spec		Glycopyrroni	#N/A	2	0102000L0AAADAD	TRUE
Glycopyrroni	0102000L0AAAZAZ	14200	Oral Soln	Liq Spec		Glycopyrroni	#N/A	2	0102000L0AAAIAI	TRUE
Glycopyrroni	0102000L0AABABA	31330	Oral Susp	Liq Spec		Glycopyrroni	#N/A	2	0102000L0AAAIAI	TRUE
Glycopyrroni	0102000L0AABBBB	2400	Oral Soln	Oral Susp	Y	Glycopyrroni	0102000L0AA	2	0102000L0AABCBC	TRUE
Glycopyrroni	0102000L0AABCBC	7062	Oral Susp	Oral Soln	Y	Glycopyrroni	0102000L0AA	2	0102000L0AABBBB	TRUE
Glycopyrroni	0102000L0AABDBD	13880	Oral Soln	Liq Spec		Glycopyrroni	#N/A	2	0102000L0AAAEAE	TRUE
Glycopyrroni	0102000L0AABEBE	13430	Oral Susp	Liq Spec		Glycopyrroni	#N/A	2	0102000L0AAAEAE	TRUE
Glycopyrroni	0102000L0AABFBF	24018	Oral Soln	Liq Spec		Glycopyrroni	#N/A	2	0102000L0AAAKAK	TRUE
Glycopyrroni	0102000L0AABGBG	16815	Oral Susp	Liq Spec		Glycopyrroni	#N/A	2	0102000L0AAAKAK	TRUE
Glycopyrroni	0102000L0AABHBH	17850	Oral Soln	Liq Spec		Glycopyrroni	#N/A	2	0102000L0AAAJAJ	TRUE
Glycopyrroni	0102000L0AABIBI	15460	Oral Susp	Liq Spec		Glycopyrroni	#N/A	2	0102000L0AAAJAJ	TRUE
Hyoscine But	0102000N0AAAPAP	13640	Oral Soln	Liq Spec		Hyoscine But	#N/A	2	0102000N0AAAGAG	TRUE
Hyoscine But	0102000N0AAAQAQ	5570	Oral Susp	Liq Spec		Hyoscine But	#N/A	2	0102000N0AAAGAG	TRUE
Mebeverine	0102000P0AAABAB	10601606	Tab	Oral Pdr Sach	N	Mebeverine	0102000P0AA	1	0102000P0AAAEAE	TRUE
Mebeverine	0102000P0AAAEAE	84	Oral Pdr Sach	Tab	N	Mebeverine	0102000P0AA	3	0102000P0AAABAB	TRUE
Propanthelin	0102000Y0AABBBB	225	Liq Spec	Mix		Propanthelin	#N/A	2	0102000Y0AAAEAE	TRUE
Cimetidine_T	0103010D0AAAAAA	8971	Tab	Tab Chble		Cimetidine_T	#N/A	1	0103010D0AAAFAF	TRUE
Cimetidine_(	0103010D0AAALAL	43560	Oral Soln	Oral Susp		Cimetidine_(	#N/A	2	0103010D0AAAGAG	TRUE
Ranitidine H(	0103010T0AAAAAA	16933447	Tab	Cap		Ranitidine H(	#N/A	1	0103010T0AABJBJ	TRUE
Ranitidine H(	0103010T0AAACAC	3037892	Tab	Tab Eff	N	Ranitidine H(	0103010T0AA	1	0103010T0AAAJAJ	TRUE
Ranitidine H(	0103010T0AAAIAI	103545	Tab Eff	Cap		Ranitidine H(	#N/A	2	0103010T0AABJBJ	TRUE
Ranitidine H(	0103010T0AAAJAJ	23985	Tab Eff	Tab	N	Ranitidine H(	0103010T0AA	2	0103010T0AAACAC	TRUE
Ranitidine H(	0103010T0AAAPAP	54538	Tab	Tab Eff		Ranitidine H(	#N/A	1	0103010T0AABKBK	TRUE
Ranitidine H(	0103010T0AABABA	600	Liq Spec	Oral Soln		Ranitidine H(	#N/A	2	0103010T0AABLBL	TRUE
Ranitidine H(	0103010T0AABQBQ	57396	Oral Soln	Liq Spec		Ranitidine H(	#N/A	2	0103010T0AAANAN	TRUE
Ranitidine H(	0103010T0AABRBR	47162	Oral Susp	Liq Spec		Ranitidine H(	#N/A	2	0103010T0AAANAN	TRUE
Esomeprazol	0103050E0AAAAAA	1952352	Tab E/C	Cap E/C	Y	Esomeprazol	0103050E0AA	2	0103050E0AAAFAF	TRUE
Esomeprazol	0103050E0AAABAB	1827340	Tab E/C	Cap E/C	Y	Esomeprazol	0103050E0AA	2	0103050E0AAAGAG	TRUE
Esomeprazol	0103050E0AAAFAF	735398	Cap E/C	Tab E/C	Y	Esomeprazol	0103050E0AA	2	0103050E0AAAAAA	TRUE
Esomeprazol	0103050E0AAAGAG	704875	Cap E/C	Tab E/C	Y	Esomeprazol	0103050E0AA	2	0103050E0AAABAB	TRUE
Lansoprazole	0103050L0AAAHAH	1694934	Orodisper Tab	Gran Sach		Lansoprazole	#N/A	2	0103050L0AAADAD	TRUE
Lansoprazole	0103050L0AAAJAJ	3696	Oral Soln	Oral Susp	Y	Lansoprazole	0103050L0AA	2	0103050L0AAAYAY	TRUE
Lansoprazole	0103050L0AAAMAM	5208	Oral Soln	Oral Susp	Y	Lansoprazole	0103050L0AA	2	0103050L0AAAXAX	TRUE
Lansoprazole	0103050L0AAAQAQ	100	Oral Soln	Oral Susp	Y	Lansoprazole	0103050L0AA	2	0103050L0AAAZAZ	TRUE
Lansoprazole	0103050L0AAAXAX	20180	Oral Susp	Oral Soln	Y	Lansoprazole	0103050L0AA	2	0103050L0AAAMAM	TRUE
Lansoprazole	0103050L0AAAYAY	23330	Oral Susp	Oral Soln	Y	Lansoprazole	0103050L0AA	2	0103050L0AAAJAJ	TRUE
Lansoprazole	0103050L0AAAZAZ	16530	Oral Susp	Oral Soln	Y	Lansoprazole	0103050L0AA	2	0103050L0AAAQAQ	TRUE
Omeprazole_	0103050P0AAAAAA	88356671	Cap E/C	Tab E/C	Y	Omeprazole_	0103050P0AA	2	0103050P0AABDBD	TRUE
Omeprazole_	0103050P0AAAEAE	2424691	Cap E/C	Tab E/C	Y	Omeprazole_	0103050P0AA	2	0103050P0AABEBE	TRUE
Omeprazole_	0103050P0AAAFAF	6546668	Cap E/C	Cap		Omeprazole_	#N/A	2	0103050P0AAABAB	TRUE
Omeprazole_	0103050P0AAAJAJ	5380	Oral Soln	Oral Susp	Y	Omeprazole_	0103050P0AA	2	0103050P0AABLBL	TRUE
Omeprazole_	0103050P0AAAKAK	3055	Oral Soln	Oral Susp	Y	Omeprazole_	0103050P0AA	2	0103050P0AABPBP	TRUE
Omeprazole_	0103050P0AAAQAQ	4190	Oral Soln	Oral Susp	Y	Omeprazole_	0103050P0AA	2	0103050P0AABMBM	TRUE
Omeprazole_	0103050P0AABCBC	77687	Tab E/C	Cap	Y	Omeprazole_	#N/A	2	0103050P0AAABAB	TRUE
Omeprazole_	0103050P0AABDBD	698699	Tab E/C	Cap E/C	Y	Omeprazole_	0103050P0AA	2	0103050P0AAAAAA	TRUE
Omeprazole_	0103050P0AABEBE	87206	Tab E/C	Cap E/C	Y	Omeprazole_	0103050P0AA	2	0103050P0AAAEAE	TRUE
Omeprazole_	0103050P0AABLBL	355694	Oral Susp	Oral Soln	Y	Omeprazole_	0103050P0AA	2	0103050P0AAAJAJ	TRUE

Omeprazole_	0103050P0AABMBM	193620	Oral Susp	Oral Soln	Y	Omeprazole_	0103050P0AA	2	0103050P0AAAQAQ	TRUE
Omeprazole_	0103050P0AABNBN	93949	Oral Susp	Liq Spec		Omeprazole_	#N/A	2	0103050P0AAAIAI	TRUE
Omeprazole_	0103050P0AABPBP	10390	Oral Susp	Oral Soln	Y	Omeprazole_	0103050P0AA	2	0103050P0AAAKAK	TRUE
Loperamide I	0104020L0AAAAAA	9462179	Cap	Tab	Y	Loperamide I	0104020L0AA	1	0104020L0AAADAD	TRUE
Loperamide I	0104020L0AAABAB	1143729	Oral Soln	Liq		Loperamide I	#N/A	2	0104020L0AAAEAE	TRUE
Loperamide I	0104020L0AAADAD	1434945	Tab	Cap	Y	Loperamide I	0104020L0AA	1	0104020L0AAAAAA	TRUE
Loperamide I	0104020L0AAAPAP	10170	Oral Susp	Oral Soln	Y	Loperamide I	0104020L0AA	2	0104020L0AAAQAQ	TRUE
Loperamide I	0104020L0AAAQAQ	2450	Oral Soln	Oral Susp	Y	Loperamide I	0104020L0AA	2	0104020L0AAAPAP	TRUE
Mesalazine_	0105010B0AAABAB	56389	Suppos	Tab G/R	N	Mesalazine_	0105010B0AA	1	0105010B0AAAWAW	TRUE
Mesalazine_	0105010B0AAAGAG	12640	Suppos	Tab E/C	Y	Mesalazine_	0105010B0AA	1	0105010B0AAAHAH	TRUE
Mesalazine_	0105010B0AAAHAH	15870	Tab E/C	Suppos	N	Mesalazine_	0105010B0AA	2	0105010B0AAAGAG	TRUE
Mesalazine_	0105010B0AAAIAI	725598	Tab	Gran Sach	N	Mesalazine_	0105010B0AA	1	0105010B0AAAPAP	TRUE
Mesalazine_	0105010B0AAANAN	47767	Gran Sach	Gran Sach G/R	Y	Mesalazine_	0105010B0AA	2	0105010B0AAAXAX	TRUE
Mesalazine_	0105010B0AAAPAP	28941	Gran Sach	Tab	Y	Mesalazine_	0105010B0AA	2	0105010B0AAAIAI	TRUE
Mesalazine_	0105010B0AAAWAW	18574	Tab G/R	Suppos	N	Mesalazine_	0105010B0AA	2	0105010B0AAABAB	TRUE
Mesalazine_	0105010B0AAAXAX	53999	Gran Sach G/R	Gran Sach	Y	Mesalazine_	0105010B0AA	3	0105010B0AAANAN	TRUE
Sulfasalazine	0105010E0AAAAAA	2372151	Tab	Suppos	N	Sulfasalazine	0105010E0AA	1	0105010E0AAACAC	TRUE
Sulfasalazine	0105010E0AAABAB	3736217	Tab E/C	Suppos	Y	Sulfasalazine	0105010E0AA	2	0105010E0AAACAC	TRUE
Sulfasalazine	0105010E0AAACAC	1383	Suppos	Tab	N	Sulfasalazine	0105010E0AA	1	0105010E0AAAAAA	TRUE
Sulfasalazine	0105010E0AAAEAE	8000	Oral Susp	Liq Spec		Sulfasalazine	#N/A	2	0105010E0AAAIAI	TRUE
Ispag Husk_C	0106010E0AAAHAH	229169	Gran Eff Sach	Pdr Sach		Ispag Husk_P	#N/A	3	0106010E0AAASAS	TRUE
Bisacodyl_Ta	0106020C0AAAAAA	4924625	Tab E/C	Suppos	N	Bisacodyl_Su	0106020C0AA	2	0106020C0AAADAD	TRUE
Bisacodyl_Su	0106020C0AAADAD	21633	Suppos	Tab E/C	N	Bisacodyl_Ta	0106020C0AA	1	0106020C0AAAAAA	TRUE
Bisacodyl_Su	0106020C0AAAEAE	72239	Suppos	Enema	N	Bisacodyl_En	0106020C0AA	1	0106020C0AAAJAJ	TRUE
Bisacodyl_En	0106020C0AAAJAJ	848	Enema	Suppos	Y	Bisacodyl_Su	0106020C0AA	1	0106020C0AAAEAE	TRUE
Docusate So	0106020I0AAAJAJ	2973	Micro-Enem	Suppos		Docusate So	#N/A	1	0106020I0AAAMAM	TRUE
Docusate So	0106020I0AAAKAK	9752953	Cap	Tab		Docusate So	#N/A	1	0106020I0AAADAD	TRUE
Senna_Tab 1	0106020M0AAAPAP	250443	Tab	Tab Chble	N	Senna_Tab C	#N/A	1	0106020M0AAAQAQ	TRUE
Diltiazem HC	0107010AAAAAJAJ	41610	Crm	Gel		Diltiazem HC	#N/A	1	0107010AAAAABAB	TRUE
Diltiazem HC	0107010AAAAAKAK	12590	Oint	Crm	Y	Diltiazem HC	0107010AAAA	1	0107010AAAAAJAJ	TRUE
Glyceryl Trini	0107040A0AAAIAI	129420	Oint	Paste		Glyceryl Trini	#N/A	1	0107040A0AAAFAF	TRUE
Glyceryl Trini	0107040A0AAAWAW	1410	Oint	Paste		Glyceryl Trini	#N/A	1	0107040A0AAAGAG	TRUE
Chenodeoxy	0109010G0AAABAB	60	Cap	Tab		Chenodeoxy	#N/A	1	0109010G0AAACAC	TRUE
Ursodeoxych	0109010U0AAAAAA	345655	Tab	Cap	Y	Ursodeoxych	#N/A	1	0109010U0AAAHAH	TRUE
Pancreatin_C	0109040N0AAAZAZ	224	G/R Cap	Cap		Pancreatin_C	#N/A	2	0109040N0AAAGAG	TRUE
Bendroflume	0202010B0AAABAB	35856484	Tab	Cap	Y	Bendroflume	#N/A	1	0202010B0AAATAT	TRUE
Bendroflume	0202010B0AAACAC	786594	Tab	Cap	Y	Bendroflume	#N/A	1	0202010B0AAARAR	TRUE
Bendroflume	0202010B0AAAQAQ	230	Liq Spec	Syr		Bendroflume	#N/A	2	0202010B0AAALAL	TRUE
Bendroflume	0202010B0AAAUAU	300	Liq Spec	Mix		Bendroflume	#N/A	2	0202010B0AAAGAG	TRUE
Bendroflume	0202010B0AAAXAX	12350	Oral Susp	Liq Spec		Bendroflume	#N/A	2	0202010B0AAAPAP	TRUE
Chloroth_Ora	0202010D0AAAUAU	18393	Oral Susp	Oral Soln	Y	Chloroth_Ora	0202010D0AA	2	0202010D0AABCBC	TRUE
Chloroth_Ora	0202010D0AABCBC	3900	Oral Soln	Oral Susp	Y	Chloroth_Ora	0202010D0AA	2	0202010D0AAAUAU	TRUE
Chloroth_Liq	0202010D0AABIBI	1720	Liq Spec	Susp		Chloroth_Sus	#N/A	2	0202010D0AAATAT	TRUE
Chlortalidone	0202010F0AAAAAA	29140	Tab	Pdrs		Chlortalidone	#N/A	1	0202010F0AAAJAJ	TRUE
Hydchloroth_	0202010L0AAABAB	3170	Tab	Cap		Hydchloroth_	#N/A	1	0202010L0AAAWAW	TRUE
Indapamide_	0202010P0AAAAAA	8333460	Tab	Cap	Y	Indapamide_	#N/A	1	0202010P0AAACAC	TRUE
Metolazone_	0202010V0AAANAN	15552	Tab	Cap		Metolazone_	#N/A	1	0202010V0AAABAB	TRUE
Furosemide_	0202020L0AABBBB	9846615	Tab	Cap	Y	Furosemide_	#N/A	1	0202020L0AACUCU	TRUE
Furosemide_	0202020L0AABDBD	23302639	Tab	Cap	Y	Furosemide_	#N/A	1	0202020L0AACWCW	TRUE
Furosemide_	0202020L0AABYBY	485	Liq Spec	Mix		Furosemide_	#N/A	2	0202020L0AAAWAW	TRUE
Furosemide_	0202020L0AABZBZ	530	Liq Spec	Mix		Furosemide_	#N/A	2	0202020L0AAAVAV	TRUE
Furosemide_	0202020L0AACACA	5000	Liq Spec	Oral Soln		Furosemide_	#N/A	2	0202020L0AADJDJ	TRUE
Amiloride HC	0202030C0AAASAS	21172	Oral Soln	Soln		Amiloride HC	#N/A	2	0202030C0AAAIAI	TRUE
Spironol_Tab	0202030S0AAATAT	4415864	Tab	Tab E/C	Y	Spironol_Tab	#N/A	1	0202030S0AAARAR	TRUE
Spironol_Tab	0202030S0AAAUAU	791773	Tab	Cap	Y	Spironol_Cap	#N/A	1	0202030S0AADZDZ	TRUE
Spironol_Tab	0202030S0AAAVAV	605010	Tab	Cap	Y	Spironol_Cap	#N/A	1	0202030S0AAABAB	TRUE
Spironol_Ora	0202030S0AACMCM	2850	Oral Soln	Oral Susp	Y	Spironol_Ora	0202030S0AA	2	0202030S0AAECEC	TRUE
Spironol_Ora	0202030S0AACNCN	3838	Oral Soln	Oral Susp	Y	Spironol_Ora	0202030S0AA	2	0202030S0AAEAEA	TRUE
Spironol_Ora	0202030S0AACPCP	5140	Oral Soln	Oral Susp	Y	Spironol_Ora	0202030S0AA	2	0202030S0AAEBEB	TRUE
Spironol_Ora	0202030S0AACQCQ	1400	Oral Soln	Oral Susp	Y	Spironol_Ora	0202030S0AA	2	0202030S0AAEDED	TRUE
Spironol_Liq	0202030S0AACRCR	2010	Liq Spec	Oral Susp	Y	Spironol_Ora	0202030S0AA	2	0202030S0AAEEEE	TRUE
Spironol_Liq	0202030S0AACWCW	300	Liq Spec	Susp		Spironol_Sus	#N/A	2	0202030S0AABJBJ	TRUE
Spironol_Liq	0202030S0AADCDC	500	Liq Spec	Liq	Y	Spironol_Liq	#N/A	2	0202030S0AABYBY	TRUE
Spironol_Ora	0202030S0AAEAEA	30316	Oral Susp	Oral Soln	Y	Spironol_Ora	0202030S0AA	2	0202030S0AACNCN	TRUE
Spironol_Ora	0202030S0AAEBEB	53225	Oral Susp	Oral Soln	Y	Spironol_Ora	0202030S0AA	2	0202030S0AACPCP	TRUE
Spironol_Ora	0202030S0AAECEC	19719	Oral Susp	Oral Soln	Y	Spironol_Ora	0202030S0AA	2	0202030S0AACMCM	TRUE
Spironol_Ora	0202030S0AAEDED	16970	Oral Susp	Oral Soln	Y	Spironol_Ora	0202030S0AA	2	0202030S0AACQCQ	TRUE
Spironol_Ora	0202030S0AAEEEE	4125	Oral Susp	Liq Spec	Y	Spironol_Liq	0202030S0AA	2	0202030S0AACRCR	TRUE
Co-Amilofrus	0202040B0AAAHAH	400	Liq Spec	Susp		Co-Amilofrus	#N/A	2	0202040B0AAADAD	TRUE
Amiodarone	0203020D0AAAUAU	1180	Oral Soln	Oral Susp	Y	Amiodarone	0203020D0AA	2	0203020D0AACHCH	TRUE
Amiodarone	0203020D0AAAVAV	850	Liq Spec	Susp		Amiodarone	#N/A	2	0203020D0AAARAR	TRUE
Amiodarone	0203020D0AAAYAY	400	Oral Soln	Oral Susp	Y	Amiodarone	0203020D0AA	2	0203020D0AACICI	TRUE
Amiodarone	0203020D0AABEBE	100	Liq Spec	Susp		Amiodarone	#N/A	2	0203020D0AAAIAI	TRUE
Amiodarone	0203020D0AACHCH	2060	Oral Susp	Oral Soln	Y	Amiodarone	0203020D0AA	2	0203020D0AAAUAU	TRUE
Amiodarone	0203020D0AACICI	1900	Oral Susp	Oral Soln	Y	Amiodarone	0203020D0AA	2	0203020D0AAAYAY	TRUE
Disopyramid	0203020F0AAABAB	100348	Cap	Tab		Disopyramid	#N/A	1	0203020F0AAAGAG	TRUE
Disopyramid	0203020F0AAACAC	14552	Cap	Tab		Disopyramid	#N/A	1	0203020F0AAAHAH	TRUE
Disopyramid	0203020F0AAAPAP	112	Cap	Tab		Disopyramid	#N/A	1	0203020F0AAAFAF	TRUE
Disopyramid	0203020G0AAACAC	25446	Tab	Cap		Disopyramid	#N/A	1	0203020G0AAABAB	TRUE
Flecainide Ac	0203020I0AAAKAK	1555807	Tab	Pdrs		Flecainide Ac	#N/A	1	0203020I0AAAEAE	TRUE
Flecainide Ac	0203020I0AABRBR	59050	Oral Soln	Liq Spec		Flecainide Ac	#N/A	2	0203020I0AAAMAM	TRUE
Flecainide Ac	0203020I0AABSBS	30430	Oral Susp	Liq Spec		Flecainide Ac	#N/A	2	0203020I0AAAMAM	TRUE
Mexiletine H	0203020P0AAABAB	11226	Cap	Tab	Y	Mexiletine H	0203020P0AA	1	0203020P0AAAGAG	TRUE
Mexiletine H	0203020P0AAAGAG	1371	Tab	Cap	Y	Mexiletine H	0203020P0AA	1	0203020P0AAABAB	TRUE

Quinidine Su	0203020U0AAAGAG	846	Tab	Cap		Quinidine Su	#N/A	1	0203020U0AAAHAH	TRUE
Carvedilol_Ta	020400080AAACAC	581723	Tab	Cap	Y	Carvedilol_Ca	#N/A	1	020400080AAAAAA	TRUE
Carvedilol_O	020400080AAAPAP	13650	Oral Susp	Liq Spec		Carvedilol_Li	#N/A	2	020400080AAAGAG	TRUE
Atenolol_Tab	0204000E0AAACAC	3302236	Tab	Cap	Y	Atenolol_Cap	#N/A	1	0204000E0AAAHAH	TRUE
Bisoprolol Fu	0204000H0AAAAAA	13527616	Tab	Pdrs		Bisoprolol Fu	#N/A	1	0204000H0AAATAT	TRUE
Bisoprolol Fu	0204000H0AAABAB	5902452	Tab	Pdr Sach		Bisoprolol Fu	#N/A	1	0204000H0AAAYAY	TRUE
Bisoprolol Fu	0204000H0AAAJAJ	20978219	Tab	Pdr Sach		Bisoprolol Fu	#N/A	1	0204000H0AABCBC	TRUE
Bisoprolol Fu	0204000H0AABEBE	30405	Oral Soln	Liq Spec		Bisoprolol Fu	#N/A	2	0204000H0AAAPAP	TRUE
Bisoprolol Fu	0204000H0AABFBF	4478	Oral Susp	Liq Spec		Bisoprolol Fu	#N/A	2	0204000H0AAAPAP	TRUE
Bisoprolol Fu	0204000H0AABGBG	7550	Oral Soln	Liq Spec		Bisoprolol Fu	#N/A	2	0204000H0AAAQAQ	TRUE
Bisoprolol Fu	0204000H0AABHBH	2970	Oral Susp	Liq Spec		Bisoprolol Fu	#N/A	2	0204000H0AAAQAQ	TRUE
Bisoprolol Fu	0204000H0AABIBI	2990	Oral Susp	Oral Soln		Bisoprolol Fu	#N/A	2	0204000H0AAAUAU	TRUE
Metoprolol T	0204000K0AABKBK	7100	Oral Soln	Liq Spec		Metoprolol T	#N/A	2	0204000K0AAAUAU	TRUE
Metoprolol T	0204000K0AABLBL	1350	Oral Susp	Liq Spec		Metoprolol T	#N/A	2	0204000K0AAAUAU	TRUE
Metoprolol T	0204000K0AABMBM	2320	Oral Soln	Liq Spec		Metoprolol T	#N/A	2	0204000K0AAATAT	TRUE
Metoprolol T	0204000K0AABNBN	920	Oral Susp	Liq Spec		Metoprolol T	#N/A	2	0204000K0AAATAT	TRUE
Propranolol I	0204000R0AAAHAH	8556246	Tab	Cap	Y	Propranolol I	#N/A	1	0204000R0AACGCG	TRUE
Propranolol I	0204000R0AAAJAJ	9547001	Tab	Cap	Y	Propranolol I	#N/A	1	0204000R0AADJDJ	TRUE
Propranolol I	0204000R0AAALAL	26329	Tab	Cap		Propranolol I	#N/A	1	0204000R0AACVCV	TRUE
Propranolol I	0204000R0AACHCH	300	Liq Spec	Oral Soln		Propranolol I	#N/A	2	0204000R0AAAGAG	TRUE
Propranolol I	0204000R0AACICI	750	Liq Spec	Liq	Y	Propranolol I	#N/A	2	0204000R0AABUBU	TRUE
Propranolol I	0204000R0AACJCJ	300	Liq Spec	Mix		Propranolol I	#N/A	2	0204000R0AAAQAQ	TRUE
Propranolol I	0204000R0AACLCL	900	Liq Spec	Oral Soln		Propranolol I	#N/A	2	0204000R0AAAZAZ	TRUE
Sotalol HCl_(	0204000T0AAATAT	900	Oral Soln	Oral Susp	Y	Sotalol HCl_(	0204000T0AA	2	0204000T0AABCBC	TRUE
Sotalol HCl_(	0204000T0AABCBC	2705	Oral Susp	Oral Soln	Y	Sotalol HCl_(	0204000T0AA	2	0204000T0AAATAT	TRUE
Hydralazine I	0205010J0AAA3A3	3480	Liq Spec	Susp		Hydralazine I	#N/A	2	0205010J0AAAPAP	TRUE
Hydralazine I	0205010J0AAA4A4	450	Liq Spec	Susp		Hydralazine I	#N/A	2	0205010J0AAAVAV	TRUE
Hydralazine I	0205010J0AAA8A8	400	Liq Spec	Susp		Hydralazine I	#N/A	2	0205010J0AAARAR	TRUE
Methyldopa_	0205020H0AAADAD	328565	Tab	Cap	Y	Methyldopa_	#N/A	1	0205020H0AAAAAA	TRUE
Methyldopa_	0205020H0AAAIAI	300	Liq Spec	Susp		Methyldopa_	#N/A	2	0205020H0AAABAB	TRUE
Doxazosin M	0205040D0AAAAAA	2646966	Tab	Cap	Y	Doxazosin M	#N/A	1	0205040D0AAAEAE	TRUE
Doxazosin M	0205040D0AAABAB	5549195	Tab	Cap	Y	Doxazosin M	#N/A	1	0205040D0AAAGAG	TRUE
Doxazosin M	0205040D0AAACAC	14750955	Tab	Cap	Y	Doxazosin M	#N/A	1	0205040D0AAAFAF	TRUE
Doxazosin M	0205040D0AAAXAX	8550	Oral Soln	Liq Spec		Doxazosin M	#N/A	2	0205040D0AAALAL	TRUE
Doxazosin M	0205040D0AAAYAY	2300	Oral Susp	Liq Spec		Doxazosin M	#N/A	2	0205040D0AAALAL	TRUE
Doxazosin M	0205040D0AAAZAZ	2250	Oral Soln	Liq Spec		Doxazosin M	#N/A	2	0205040D0AAAMAM	TRUE
Doxazosin M	0205040D0AABABA	3870	Oral Susp	Liq Spec		Doxazosin M	#N/A	2	0205040D0AAAMAM	TRUE
Phenoxybenz	0205040M0AAACAC	11681	Cap	Tab		Phenoxybenz	#N/A	1	0205040M0AAAIAI	TRUE
Prazosin HCl_	0205040S0AAACAC	318024	Tab	Cap	Y	Prazosin HCl_	#N/A	1	0205040S0AAAMAM	TRUE
Captopril_Ta	0205051F0AAADAD	76459	Tab	Cap	Y	Captopril_Ca	#N/A	1	0205051F0AABEBE	TRUE
Captopril_Ta	0205051F0AAAEAE	211270	Tab	Cap	Y	Captopril_Ca	#N/A	1	0205051F0AABLBL	TRUE
Captopril_Ta	0205051F0AAAFAF	234980	Tab	Cap	Y	Captopril_Ca	#N/A	1	0205051F0AADUDU	TRUE
Captopril_Lic	0205051F0AABNBN	2000	Liq Spec	Oral Soln		Captopril_Or	#N/A	2	0205051F0AADVDV	TRUE
Captopril_Lic	0205051F0AABRBR	1200	Liq Spec	Susp		Captopril_Su	#N/A	2	0205051F0AABGBG	TRUE
Captopril_Lic	0205051F0AABWBW	3785	Liq Spec	Oral Soln		Captopril_Or	#N/A	2	0205051F0AADXDX	TRUE
Captopril_Lic	0205051F0AABXBX	600	Liq Spec	Susp		Captopril_Su	#N/A	2	0205051F0AAAGAG	TRUE
Enalapril Mal	0205051I0AAAAAA	409495	Tab	Cap	Y	Enalapril Mal	#N/A	1	0205051I0AABXBX	TRUE
Enalapril Mal	0205051I0AAABAB	1136019	Tab	Wafer	N	Enalapril Mal	#N/A	1	0205051I0AABIBI	TRUE
Enalapril Mal	0205051I0AAACAC	1993781	Tab	Wafer	N	Enalapril Mal	#N/A	1	0205051I0AABJBJ	TRUE
Enalapril Mal	0205051I0AAADAD	3120715	Tab	Wafer	N	Enalapril Mal	#N/A	1	0205051I0AABKBK	TRUE
Enalapril Mal	0205051I0AABYBY	15150	Oral Soln	Liq Spec		Enalapril Mal	#N/A	2	0205051I0AAANAN	TRUE
Enalapril Mal	0205051I0AABZBZ	5295	Oral Susp	Liq Spec		Enalapril Mal	#N/A	2	0205051I0AAANAN	TRUE
Lisinopril_Liq	0205051L0AAAGAG	7525	Liq Spec	Oral Soln		Lisinopril_Or	#N/A	2	0205051L0AAAUAU	TRUE
Lisinopril_Liq	0205051L0AAAIAI	2560	Liq Spec	Oral Soln		Lisinopril_Or	#N/A	2	0205051L0AAAWAW	TRUE
Lisinopril_Or	0205051L0AAAYAY	1150	Oral Soln	Liq Spec		Lisinopril_Liq	#N/A	2	0205051L0AAAFAF	TRUE
Lisinopril_Or	0205051L0AAAZAZ	1950	Oral Susp	Liq Spec		Lisinopril_Liq	#N/A	2	0205051L0AAAFAF	TRUE
Perindopril E	0205051M0AAAAAA	3258652	Tab	Pdr Sach		Perindopril E	#N/A	1	0205051M0AAAJAJ	TRUE
Perindopril E	0205051M0AAABAB	5944667	Tab	Pdr Sach		Perindopril E	#N/A	1	0205051M0AAAIAI	TRUE
Perindopril E	0205051M0AAAKAK	4800	Oral Soln	Liq Spec		Perindopril E	#N/A	2	0205051M0AAAGAG	TRUE
Perindopril E	0205051M0AAALAL	1225	Oral Susp	Liq Spec		Perindopril E	#N/A	2	0205051M0AAAGAG	TRUE
Ramipril_Cap	0205051R0AAAAAA	6842531	Cap	Tab	Y	Ramipril_Tab	0205051R0AA	1	0205051R0AAAKAK	TRUE
Ramipril_Cap	0205051R0AAABAB	18762634	Cap	Tab	Y	Ramipril_Tab	0205051R0AA	1	0205051R0AAALAL	TRUE
Ramipril_Cap	0205051R0AAACAC	22071838	Cap	Tab	Y	Ramipril_Tab	0205051R0AA	1	0205051R0AAAMAM	TRUE
Ramipril_Cap	0205051R0AAADAD	30689703	Cap	Tab	Y	Ramipril_Tab	0205051R0AA	1	0205051R0AAANAN	TRUE
Ramipril_Liq	0205051R0AAAEAE	6030	Liq Spec	Oral Soln		Ramipril_Ora	#N/A	2	0205051R0AAAXAX	TRUE
Ramipril_Liq	0205051R0AAAFAF	2530	Liq Spec	Oral Soln		Ramipril_Ora	#N/A	2	0205051R0AAAVAV	TRUE
Ramipril_Tab	0205051R0AAAKAK	322223	Tab	Cap	Y	Ramipril_Cap	0205051R0AA	1	0205051R0AAAAAA	TRUE
Ramipril_Tab	0205051R0AAALAL	683547	Tab	Cap	Y	Ramipril_Cap	0205051R0AA	1	0205051R0AAABAB	TRUE
Ramipril_Tab	0205051R0AAAMAM	984243	Tab	Cap	Y	Ramipril_Cap	0205051R0AA	1	0205051R0AAACAC	TRUE
Ramipril_Tab	0205051R0AAANAN	973626	Tab	Cap	Y	Ramipril_Cap	0205051R0AA	1	0205051R0AAADAD	TRUE
Ramipril_Titr	0205051R0AAAUAU	50	Titration Pack (Ta	Titration Pack (Cap		Ramipril_Titr	#N/A	3	0205051R0AAAIAI	TRUE
Irbesartan_T	0205052I0AAACAC	2372253	Tab	Pdr Sach		Irbesartan_P	#N/A	1	0205052I0AAAIAI	TRUE
Losartan Pot	0205052N0AAAEAE	1670	Oral Soln	Oral Susp	Y	Losartan Pot	0205052N0AA	2	0205052N0AAAJAJ	TRUE
Losartan Pot	0205052N0AAAJAJ	7540	Oral Susp	Oral Soln	Y	Losartan Pot	0205052N0AA	2	0205052N0AAAEAE	TRUE
Valsartan_Ca	0205052V0AAAAAA	446411	Cap	Tab	Y	Valsartan_Ta	0205052V0AA	1	0205052V0AAADAD	TRUE
Valsartan_Ca	0205052V0AAABAB	893555	Cap	Tab	Y	Valsartan_Ta	0205052V0AA	1	0205052V0AAAIAI	TRUE
Valsartan_Ca	0205052V0AAACAC	499682	Cap	Tab	Y	Valsartan_Ta	0205052V0AA	1	0205052V0AAAHAH	TRUE
Valsartan_Ta	0205052V0AAADAD	52315	Tab	Cap	Y	Valsartan_Ca	0205052V0AA	1	0205052V0AAAAAA	TRUE
Valsartan_Ta	0205052V0AAAHAH	5799	Tab	Cap	Y	Valsartan_Ca	0205052V0AA	1	0205052V0AAACAC	TRUE
Valsartan_Ta	0205052V0AAAIAI	7291	Tab	Cap	Y	Valsartan_Ca	0205052V0AA	1	0205052V0AAABAB	TRUE
Glyceryl Trini	0206010F0AAAIAI	100	Tab	Patch		Glyceryl Trini	#N/A	1	0206010F0AAAZAZ	TRUE
Glyceryl Trini	0206010F0AACGCG	28554	Sub A/Spy	Sub P/Spy	Y	Glyceryl Trini	0206010F0AA	2	0206010F0AACICI	TRUE
Glyceryl Trini	0206010F0AACHCH	5517	Sub A/Spy	Sub P/Spy	Y	Glyceryl Trini	0206010F0AA	2	0206010F0AACJCJ	TRUE
Glyceryl Trini	0206010F0AACICI	87127	Sub P/Spy	Sub A/Spy	Y	Glyceryl Trini	0206010F0AA	2	0206010F0AACGCG	TRUE

Glyceryl Trini	0206010F0AACJCJ	14494	Sub P/Spy	Sub A/Spy	Y	Glyceryl Trini	0206010F0AA	2	0206010F0AACHCH	TRUE
Isosorbide Di	0206010I0AAAIAI	82807	Tab	Cap	Y	Isosorbide Di	#N/A	1	0206010I0AAAAAA	TRUE
Isosorbide Di	0206010I0AAAJAJ	27919	Tab	Cap		Isosorbide Di	#N/A	1	0206010I0AAABAB	TRUE
Isosorbide M	0206010K0AAAEAE	1233653	Tab	Cap	Y	Isosorbide M	0206010K0AA	1	0206010K0AAAQAQ	TRUE
Isosorbide M	0206010K0AAAFAF	169753	Cap	Tab	Y	Isosorbide M	0206010K0AA	1	0206010K0AAAUAU	TRUE
Isosorbide M	0206010K0AAAGAG	157165	Tab	Cap	Y	Isosorbide M	0206010K0AA	1	0206010K0AAAPAP	TRUE
Isosorbide M	0206010K0AAAHAH	546345	Cap	Tab	Y	Isosorbide M	0206010K0AA	1	0206010K0AAATAT	TRUE
Isosorbide M	0206010K0AAALAL	1560	Oral Soln	Oral Susp	Y	Isosorbide M	0206010K0AA	2	0206010K0AABBBB	TRUE
Isosorbide M	0206010K0AAAPAP	274219	Cap	Tab	Y	Isosorbide M	0206010K0AA	1	0206010K0AAAGAG	TRUE
Isosorbide M	0206010K0AAAQAQ	316403	Cap	Tab	Y	Isosorbide M	0206010K0AA	1	0206010K0AAAEAE	TRUE
Isosorbide M	0206010K0AAATAT	161422	Tab	Cap	Y	Isosorbide M	0206010K0AA	1	0206010K0AAAHAH	TRUE
Isosorbide M	0206010K0AAAUAU	60893	Tab	Cap	Y	Isosorbide M	0206010K0AA	1	0206010K0AAAFAF	TRUE
Isosorbide M	0206010K0AABBBB	3160	Oral Susp	Oral Soln	Y	Isosorbide M	0206010K0AA	2	0206010K0AAALAL	TRUE
Amlodipine_	0206020A0AAACAC	13730	Liq Spec	Oral Soln		Amlodipine_	#N/A	2	0206020A0AAAQAQ	TRUE
Amlodipine_	0206020A0AAADAD	3080	Liq Spec	Oral Soln		Amlodipine_	#N/A	2	0206020A0AAASAS	TRUE
Diltiazem HC	0206020C0AAAAAA	881384	Tab	Cap	Y	Diltiazem HC	0206020C0AA	1	0206020C0AAAJAJ	TRUE
Diltiazem HC	0206020C0AAACAC	219823	Tab	Cap	Y	Diltiazem HC	0206020C0AA	1	0206020C0AAATAT	FALSE
Diltiazem HC	0206020C0AAAJAJ	291054	Cap	Tab	Y	Diltiazem HC	0206020C0AA	1	0206020C0AAAAAA	FALSE
Diltiazem HC	0206020C0AAARAR	2160	Oral Soln	Oral Susp	Y	Diltiazem HC	0206020C0AA	2	0206020C0AABIBI	TRUE
Diltiazem HC	0206020C0AAASAS	141828	Tab	Cap	Y	Diltiazem HC	0206020C0AA	1	0206020C0AAAUAU	FALSE
Diltiazem HC	0206020C0AAATAT	274921	Cap	Tab	Y	Diltiazem HC	0206020C0AA	1	0206020C0AAACAC	FALSE
Diltiazem HC	0206020C0AAAUAU	229536	Cap	Tab	Y	Diltiazem HC	0206020C0AA	1	0206020C0AAASAS	FALSE
Diltiazem HC	0206020C0AABIBI	3940	Oral Susp	Oral Soln	Y	Diltiazem HC	0206020C0AA	2	0206020C0AAARAR	TRUE
Nifedipine_C	0206020R0AAABAB	369143	Cap	Tab		Nifedipine_T	#N/A	1	0206020R0AAAVAV	TRUE
Nifedipine_T	0206020R0AAAEAE	213802	Tab	Cap	Y	Nifedipine_C	0206020R0AA	1	0206020R0AAAMAM	FALSE
Nifedipine_C	0206020R0AAAHAH	225468	Cap	Tab	Y	Nifedipine_T	0206020R0AA	1	0206020R0AAARAR	TRUE
Nifedipine_C	0206020R0AAAMAM	213718	Cap	Tab	Y	Nifedipine_T	0206020R0AA	1	0206020R0AAAEAE	TRUE
Nifedipine_T	0206020R0AAANAN	123950	Tab	Cap	Y	Nifedipine_C	0206020R0AA	1	0206020R0AABEBE	FALSE
Nifedipine_T	0206020R0AAAPAP	56400	Tab	Cap	Y	Nifedipine_C	0206020R0AA	1	0206020R0AABFBF	FALSE
Nifedipine_T	0206020R0AAARAR	258719	Tab	Cap	Y	Nifedipine_C	0206020R0AA	1	0206020R0AAAHAH	FALSE
Nifedipine_C	0206020R0AABEBE	95930	Cap	Tab	Y	Nifedipine_T	0206020R0AA	1	0206020R0AAANAN	TRUE
Nifedipine_C	0206020R0AABFBF	46764	Cap	Tab	Y	Nifedipine_T	0206020R0AA	1	0206020R0AAAPAP	TRUE
Nifedipine_C	0206020R0AABQBQ	4200	Oral Susp	Liq Spec		Nifedipine_Li	#N/A	2	0206020R0AAATAT	TRUE
Nifedipine_C	0206020R0AABRBR	4040	Oral Susp	Liq Spec		Nifedipine_Li	#N/A	2	0206020R0AABBBB	TRUE
Verapamil H(	0206020T0AAACAC	822364	Tab	Pdrs		Verapamil H(	#N/A	1	0206020T0AAAQAQ	TRUE
Verapamil H(	0206020T0AAAHAH	433418	Tab	Cap	Y	Verapamil H(	0206020T0AA	1	0206020T0AAAKAK	TRUE
Verapamil H(	0206020T0AAAIAI	77911	Cap	Tab	Y	Verapamil H(	0206020T0AA	1	0206020T0AAAUAU	TRUE
Verapamil H(	0206020T0AAAKAK	51749	Cap	Tab	Y	Verapamil H(	0206020T0AA	1	0206020T0AAAHAH	TRUE
Verapamil H(	0206020T0AAAUAU	417540	Tab	Cap	Y	Verapamil H(	0206020T0AA	1	0206020T0AAAIAI	TRUE
Nicorandil_T	0206030N0AAAAAA	5510850	Tab	Pdr Sach		Nicorandil_P	#N/A	1	0206030N0AAAEAE	TRUE
Moxisylyte H	0206040AIAAACAC	13086	Tab	Cap		Moxisylyte H	#N/A	1	0206040AIAAAFAF	TRUE
Heparin Sod_	0208010K0AAABAB	85	Inj	Soln	N	Heparin Sod_	0208010P0AA	1	0208010P0AAADAD	TRUE
Heparin Sod_	0208010K0AABIBI	60	Inj	Soln	N	Heparin Sod_	0208010P0AA	1	0208010P0AAABAB	TRUE
Heparin Sod_	0208010P0AAABAB	4304	Soln	Inj	N	Heparin Sod_	0208010K0AA	1	0208010K0AABIBI	TRUE
Heparin Sod_	0208010P0AAADAD	7771	Soln	Inj	Y	Heparin Sod_	0208010K0AA	1	0208010K0AAABAB	TRUE
Pentosan Pol	0208020I0AAAEAE	2608	Cap	Tab		Pentosan Pol	#N/A	1	0208020I0AAAFAF	TRUE
Warfarin Soc	0208020V0AAAAAA	23339391	Tab	Cap	Y	Warfarin Soc	0208020V0AA	1	0208020V0AABABA	TRUE
Warfarin Soc	0208020V0AAAIAI	1530	Liq Spec	Elix		Warfarin Soc	#N/A	2	0208020V0AAAGAG	TRUE
Warfarin Soc	0208020V0AABABA	28	Cap	Pdrs		Warfarin Soc	#N/A	1	0208020V0AAAYAY	TRUE
Aspirin_Tab	0209000A0AAAJAJ	7175840	Tab	Cap	Y	Aspirin_Cap	#N/A	1	0209000A0AAAZAZ	TRUE
Aspirin_Tab	0209000A0AAAKAK	11192886	Tab E/C	Cap		Aspirin_Cap	#N/A	2	0209000A0AAAZAZ	TRUE
Clopidogrel_	0209000C0AAAAAA	20507218	Tab	Pdrs		Clopidogrel_	#N/A	1	0209000C0AAACAC	TRUE
Clopidogrel_	0209000C0AAAJAJ	50350	Oral Soln	Liq Spec		Clopidogrel_	#N/A	2	0209000C0AAABAB	TRUE
Clopidogrel_	0209000C0AAAKAK	6360	Oral Susp	Liq Spec		Clopidogrel_	#N/A	2	0209000C0AAABAB	TRUE
Dipyridamole	0209000L0AAAHAH	4520	Oral Soln	Oral Susp		Dipyridamole	#N/A	2	0209000L0AAAWAW	TRUE
Tranexamic A	0211000P0AABBBB	17950	Oral Soln	Liq Spec		Tranexamic A	#N/A	2	0211000P0AAAFAF	TRUE
Tranexamic A	0211000P0AABCBC	5950	Oral Susp	Liq Spec		Tranexamic A	#N/A	2	0211000P0AAAFAF	TRUE
Tranexamic A	0211000P0AABDBD	5100	Mthwsh	Nsl Dps		Tranexamic A	#N/A	1	0211000P0AAASAS	TRUE
Atorvastatin_	0212000B0AAAAAA	18447484	Tab	Pdr Sach		Atorvastatin_	#N/A	1	0212000B0AAAJAJ	TRUE
Atorvastatin_	0212000B0AAABAB	36894614	Tab	Pdr Sach		Atorvastatin_	#N/A	1	0212000B0AAAKAK	TRUE
Atorvastatin_	0212000B0AAAFAF	3200	Oral Soln	Oral Susp	Y	Atorvastatin_	0212000B0AA	2	0212000B0AAAQAQ	TRUE
Atorvastatin_	0212000B0AAAQAQ	21060	Oral Susp	Oral Soln	Y	Atorvastatin_	0212000B0AA	2	0212000B0AAAFAF	TRUE
Colestipol H(	0212000K0AAAAAA	11208	Gran Sach	Pdr Sach	Y	Colestipol H(	0212000K0AA	2	0212000K0AAABAB	TRUE
Colestipol H(	0212000K0AAABAB	19381	Pdr Sach	Gran Sach	Y	Colestipol H(	0212000K0AA	2	0212000K0AAAAAA	TRUE
Nicotinic Aci	0212000U0AAABAB	90	Tab	Cap		Nicotinic Aci	#N/A	1	0212000U0AAATAT	TRUE
Pravastatin S	0212000X0AAAAAA	1673685	Tab	Pdr Sach		Pravastatin S	#N/A	1	0212000X0AAAIAI	TRUE
Salbutamol_I	0301011R0AAAPAP	1338528	Inha	Inha B/A	N	Salbutamol_I	0301011R0AA	1	0301011R0AABUBU	TRUE
Salbutamol_(	0301011R0AABGBG	262470	Oral Soln	Syr		Salbutamol_S	#N/A	2	0301011R0AABRBR	TRUE
Salbutamol_(	0301011R0AABMBM	196	Cap	Tab		Salbutamol_T	#N/A	1	0301011R0AABEBE	TRUE
Salbutamol_(	0301011R0AABPBP	336	Cap	Tab		Salbutamol_T	#N/A	1	0301011R0AABFBF	TRUE
Salbutamol_I	0301011R0AABUBU	97624	Inha B/A	Inha	N	Salbutamol_I	0301011R0AA	2	0301011R0AAAPAP	TRUE
Salbutamol_I	0301011R0AABZBZ	1443	Pdr For Inh	Inha		Salbutamol_I	#N/A	3	0301011R0AAAAAA	TRUE
Ephed HCl_T	0301012F0AAAAAA	18571	Tab	Cap		Ephed HCl_C	#N/A	1	0301012F0AAANAN	TRUE
Ephed HCl_T	0301012F0AAABAB	10567	Tab	Cap		Ephed HCl_C	#N/A	1	0301012F0AAAMAM	TRUE
Ipratrop Bro	0301020I0AAAAAA	5	Inha	Inha B/A		Ipratrop Bro	#N/A	1	0301020I0AAAGAG	TRUE
Theophylline	0301030S0AAADAD	44038	Cap	Tab	N	Theophylline	0301030S0AA	1	0301030S0AAANAN	TRUE
Theophylline	0301030S0AAAGAG	2600	Oral Soln	Liq Spec		Theophylline	#N/A	2	0301030S0AABCBC	TRUE
Theophylline	0301030S0AAANAN	4406	Tab	Cap	N	Theophylline	0301030S0AA	1	0301030S0AAADAD	TRUE
Theophylline	0301030S0AAAPAP	23204	Tab	Cap		Theophylline	#N/A	1	0301030S0AAAEAE	TRUE
Budesonide_	0302000K0AAADAD	4	Inha	Pdr For Inh		Budesonide_	#N/A	1	0302000K0AAAXAX	TRUE
Budesonide_	0302000K0AAAGAG	10906	Pdr For Inh	Inha		Budesonide_	#N/A	3	0302000K0AAABAB	TRUE
Montelukast	0303020G0AAACAC	427509	Tab Chble	Gran Sach	N	Montelukast	0303020G0AA	2	0303020G0AAADAD	TRUE
Montelukast	0303020G0AAADAD	267604	Gran Sach	Tab Chble	N	Montelukast	0303020G0AA	2	0303020G0AAACAC	TRUE
Ketotifen Fur	0304010AGAAACAC	14840	Tab	Cap		Ketotifen Fur	#N/A	1	0304010AGAAAAAA	TRUE

Bromphenira	0304010F0AAADAD	168	Cap	Tab		Bromphenira	#N/A	1	0304010F0AAACAC	TRUE
Chlorphenam	0304010G0AAACAC	2945226	Tab	Cap	Y	Chlorphenam	#N/A	1	0304010G0AAAIAI	TRUE
Chlorphenam	0304010G0AAAPAP	1167921	Oral Soln	Syr		Chlorphenam	#N/A	2	0304010G0AAANAN	TRUE
Cetirizine HC	0304010I0AAAAAA	14570891	Tab	Cap	Y	Cetirizine HC	0304010I0AA	1	0304010I0AAADAD	TRUE
Cetirizine HC	0304010I0AAADAD	62469	Cap	Tab	Y	Cetirizine HC	0304010I0AA	1	0304010I0AAAAAA	TRUE
Hydroxyzine	0304010J0AAAAAA	106636	Oral Soln	Liq Spec		Hydroxyzine	#N/A	2	0304010J0AAAEAE	TRUE
Hydroxyzine	0304010J0AAABAB	575289	Tab	Pdrs		Hydroxyzine	#N/A	1	0304010J0AAADAD	TRUE
Diphenhydra	0304010N0AAAGAG	41857	Tab	Cap		Diphenhydra	#N/A	1	0304010N0AAAAAA	TRUE
Diphenhydra	0304010N0AAAPAP	29430	Tab	Cap		Diphenhydra	#N/A	1	0304010N0AAARAR	TRUE
Diphenhydra	0304010N0AAAWAW	400	Liq Spec	Linct		Diphenhydra	#N/A	2	0304010N0AAAQAQ	TRUE
Promethazin	0304010W0AAALAL	1760504	Tab	Suppos		Promethazin	#N/A	1	0304010W0AAAJAJ	TRUE
Alimemazine	0304010Y0AAADAD	156966	Tab	Cap	Y	Alimemazine	#N/A	1	0304010Y0AAALAL	TRUE
Acetylcy_Gra	0307000C0AAAAAA	13674	Gran Sach	Cap		Acetylcy_Cap	#N/A	2	0307000C0AAAIAI	TRUE
Acetylcy_Tab	0307000C0AAAJAJ	6418	Tab Eff	Cap	N	Acetylcy_Cap	0307000C0AA	2	0307000C0AAAKAK	TRUE
Acetylcy_Cap	0307000C0AAAKAK	28146	Cap	Tab	Y	Acetylcy_Tab	0307000C0AA	1	0307000C0AAAMAM	TRUE
Acetylcy_Tab	0307000C0AAAMAM	26561	Tab	Cap	Y	Acetylcy_Cap	0307000C0AA	1	0307000C0AAAKAK	TRUE
Carbocistein	0307000J0AAAAAA	18236337	Cap	Tab		Carbocistein	#N/A	1	0307000J0AAAEAE	TRUE
Codeine Pho	0309010C0AAAAAA	1460883	Linct	Linct Diabetic		Codeine Pho	#N/A	1	0309010C0AAABAB	TRUE
Pholcodine_I	0309010X0AAABAB	512980	Linct	Linct Diabetic		Pholcodine_I	#N/A	1	0309010X0AAAEAE	TRUE
Pholcodine_I	0309010X0AAACAC	57005	Linct Strong	Linct Diabetic		Pholcodine_I	#N/A	2	0309010X0AAAFAF	TRUE
Guaifenesin_	0309020G0AAALAL	180	Oral Soln	Linct		Guaifenesin_	#N/A	2	0309020G0AAAFAF	TRUE
Guaifenesin_	0309020G0AAANAN	125	Oral Soln	Linct		Guaifenesin_	#N/A	2	0309020G0AAAIAI	TRUE
Guaifen/Levo	0309020G0AAAPAP	500	Oral Soln	Sach		Guaifen/Levo	#N/A	1	#N/A	TRUE
Pseudoephe	0310000N0AAABAB	89590	Oral Soln	Linct		Pseudoephe	#N/A	2	0310000N0AAAMAM	TRUE
Melatonin_T	0401010ADAAAAAA	1081719	Tab	Cap	Y	Melatonin_C	0401010ADAA	1	0401010ADAACHCH	TRUE
Melatonin_C	0401010ADAAAEAE	175402	Cap	Tab	Y	Melatonin_T	0401010ADAA	1	0401010ADAABKBK	TRUE
Melatonin_C	0401010ADAAAHAH	7082	Cap	Tab Subling		Melatonin_T	#N/A	1	0401010ADAAAVAV	TRUE
Melatonin_T	0401010ADAAAIAI	7400	Tab	Cap	Y	Melatonin_C	0401010ADAA	1	0401010ADAABQBQ	TRUE
Melatonin_C	0401010ADAAAJAJ	21288	Cap	Tab	Y	Melatonin_T	0401010ADAA	1	0401010ADAAAQAQ	TRUE
Melatonin_T	0401010ADAAAQAQ	150	Tab	Cap	Y	Melatonin_C	0401010ADAA	1	0401010ADAAAJAJ	TRUE
Melatonin_C	0401010ADAABABA	913868	Oral Soln	Liq Spec		Melatonin_Li	#N/A	2	0401010ADAABHBH	TRUE
Melatonin_T	0401010ADAABKBK	90	Tab	Cap	Y	Melatonin_C	0401010ADAA	1	0401010ADAAAEAE	TRUE
Melatonin_T	0401010ADAABLBL	1794	Tab	Cap	Y	Melatonin_C	0401010ADAA	1	0401010ADAABSBS	TRUE
Melatonin_T	0401010ADAABPBP	47585	Tab	Cap	Y	Melatonin_C	0401010ADAA	1	0401010ADAABRBR	TRUE
Melatonin_C	0401010ADAABQBQ	12073	Cap	Tab	Y	Melatonin_T	0401010ADAA	1	0401010ADAAAIAI	TRUE
Melatonin_C	0401010ADAABRBR	119844	Cap	Loz Subling		Melatonin_Lo	#N/A	1	0401010ADAABEBE	TRUE
Melatonin_C	0401010ADAABSBS	45762	Cap	Tab	Y	Melatonin_T	0401010ADAA	1	0401010ADAABLBL	TRUE
Melatonin_C	0401010ADAABXBX	70006	Oral Susp	Liq Spec		Melatonin_Li	#N/A	2	0401010ADAABHBH	TRUE
Melatonin_C	0401010ADAABYBY	64425	Oral Soln	Liq Spec		Melatonin_Li	#N/A	2	0401010ADAAAYAY	TRUE
Melatonin_C	0401010ADAABZBZ	13626	Oral Susp	Liq Spec		Melatonin_Li	#N/A	2	0401010ADAAAYAY	TRUE
Melatonin_C	0401010ADAACACA	21850	Oral Soln	Liq Spec		Melatonin_Li	#N/A	2	0401010ADAABFBF	TRUE
Melatonin_C	0401010ADAACBCB	2990	Oral Susp	Liq Spec		Melatonin_Li	#N/A	2	0401010ADAABFBF	TRUE
Melatonin_C	0401010ADAACDCD	20710	Oral Soln	Liq Spec		Melatonin_Li	#N/A	2	0401010ADAABUBU	TRUE
Melatonin_C	0401010ADAACECE	4280	Oral Susp	Liq Spec		Melatonin_Li	#N/A	2	0401010ADAABUBU	TRUE
Melatonin_C	0401010ADAACFCF	26776	Oral Soln	Liq Spec		Melatonin_Li	#N/A	2	0401010ADAAATAT	TRUE
Melatonin_C	0401010ADAACGCG	11240	Oral Susp	Liq Spec		Melatonin_Li	#N/A	2	0401010ADAAATAT	TRUE
Melatonin_C	0401010ADAACHCH	324	Cap	Tab	Y	Melatonin_T	0401010ADAA	1	0401010ADAAAAAA	TRUE
Chloral Hydra	0401010B0AAAFAF	62020	Oral Soln	Liq Spec		Chloral Hydra	#N/A	2	0401010B0AAAYAY	TRUE
Chloral Hydra	0401010B0AAAQAQ	36	Suppos	Cap		Chloral Hydra	#N/A	1	0401010B0AAAAAA	TRUE
Chloral Hydra	0401010B0AABGBG	11550	Liq Spec	Oral Susp		Chloral Hydra	#N/A	2	0401010B0AABVBV	TRUE
Chloral Hydra	0401010B0AABSBS	219458	Mix	Elix		Chloral Hydra	#N/A	1	0401010B0AAAGAG	TRUE
Lormetazepa	0401010P0AAACAC	49672	Tab	Cap		Lormetazepa	#N/A	1	0401010P0AAAAAA	TRUE
Nitrazepam_	0401010R0AAACAC	1366424	Tab	Cap	Y	Nitrazepam_	#N/A	1	0401010R0AAAIAI	TRUE
Nitrazepam_	0401010R0AAAPAP	2640	Liq Spec	Oral Susp		Nitrazepam_	#N/A	2	0401010R0AAALAL	TRUE
Temazepam_	0401010T0AAAEAE	252368	Oral Soln	Ud Oral Soln		Temazepam_	#N/A	2	0401010T0AABABA	TRUE
Zolpidem Tar	0401010Y0AAAAAA	590261	Tab	Pdr Sach		Zolpidem Tar	#N/A	1	0401010Y0AAACAC	TRUE
Zopiclone_Ta	0401010Z0AAAAAA	5169972	Tab	Pdr Sach		Zopiclone_Po	#N/A	1	0401010Z0AAAIAI	TRUE
Zopiclone_Ta	0401010Z0AAACAC	4289969	Tab	Pdr Sach		Zopiclone_Po	#N/A	1	0401010Z0AAAHAH	TRUE
Zopiclone_O	0401010Z0AAAJAJ	25500	Oral Soln	Liq Spec		Zopiclone_Li	#N/A	2	0401010Z0AAAEAE	TRUE
Zopiclone_O	0401010Z0AAAKAK	2290	Oral Susp	Liq Spec		Zopiclone_Li	#N/A	2	0401010Z0AAAEAE	TRUE
Zopiclone_O	0401010Z0AAALAL	1880	Oral Susp	Liq Spec		Zopiclone_Li	#N/A	2	0401010Z0AAAFAF	TRUE
Zopiclone_O	0401010Z0AAAMAM	6410	Oral Soln	Liq Spec		Zopiclone_Li	#N/A	2	0401010Z0AAAFAF	TRUE
Chlordiazepo	0401020E0AAAAAA	458	Tab	Cap	Y	Chlordiazepo	0401020E0AA	1	0401020E0AAADAD	TRUE
Chlordiazepo	0401020E0AAABAB	16706	Tab	Cap	Y	Chlordiazepo	0401020E0AA	1	0401020E0AAAEAE	TRUE
Chlordiazepo	0401020E0AAADAD	160372	Cap	Tab	Y	Chlordiazepo	0401020E0AA	1	0401020E0AAAAAA	TRUE
Chlordiazepo	0401020E0AAAEAE	107295	Cap	Tab	Y	Chlordiazepo	0401020E0AA	1	0401020E0AAABAB	TRUE
Chlordiazepo	0401020E0AAAUAU	100	Liq Spec	Susp		Chlordiazepo	#N/A	2	0401020E0AAAJAJ	TRUE
Diazepam_O	0401020K0AAA1A1	30113	Oral Soln	Liq Spec		Diazepam_Li	#N/A	2	0401020K0AABHBH	TRUE
Diazepam_O	0401020K0AAA6A6	71630	Oral Soln	Liq Spec		Diazepam_Li	#N/A	2	0401020K0AABNBN	TRUE
Diazepam_In	0401020K0AAACAC	77	Inj	Inj (Emulsion)	Y	Diazepam_In	0401020K0AA	1	0401020K0AAAQAQ	TRUE
Diazepam_Ta	0401020K0AAAHAH	6880790	Tab	Cap	Y	Diazepam_Ca	#N/A	1	0401020K0AAA2A2	TRUE
Diazepam_Ta	0401020K0AAAIAI	5012210	Tab	Cap	Y	Diazepam_Ca	#N/A	1	0401020K0AAA3A3	TRUE
Diazepam_Ta	0401020K0AAAJAJ	727887	Tab	Cap	Y	Diazepam_Ca	#N/A	1	0401020K0AABJBJ	TRUE
Diazepam_In	0401020K0AAAQAQ	119	Inj (Emulsion)	Inj	N	Diazepam_In	0401020K0AA	2	0401020K0AAACAC	TRUE
Diazepam_O	0401020K0AACBCB	32822	Oral Soln	Liq Spec		Diazepam_Li	#N/A	2	0401020K0AABUBU	TRUE
Lorazepam_T	0401020P0AAABAB	2686243	Tab	Cap	Y	Lorazepam_C	#N/A	1	0401020P0AAANAN	TRUE
Lorazepam_L	0401020P0AABHBH	930	Liq Spec	Susp		Lorazepam_S	#N/A	2	0401020P0AAAJAJ	TRUE
Lorazepam_O	0401020P0AACDCD	42530	Oral Soln	Liq Spec		Lorazepam_L	#N/A	2	0401020P0AABIBI	TRUE
Lorazepam_O	0401020P0AACECE	8965	Oral Susp	Liq Spec		Lorazepam_L	#N/A	2	0401020P0AABIBI	TRUE
Lorazepam_O	0401020P0AACFCF	13950	Oral Soln	Liq Spec		Lorazepam_L	#N/A	2	0401020P0AABGBG	TRUE
Lorazepam_O	0401020P0AACGCG	4400	Oral Susp	Liq Spec		Lorazepam_L	#N/A	2	0401020P0AABGBG	TRUE
Oxazepam_L	0401020T0AAAJAJ	100	Liq Spec	Susp		Oxazepam_S	#N/A	2	0401020T0AAAEAE	TRUE
Amobarb So	0401030E0AAAAAA	1541	Cap	Tab		Amobarb So	#N/A	1	0401030E0AAAEAE	TRUE
Amobarb So	0401030E0AAABAB	706	Cap	Tab		Amobarb So	#N/A	1	0401030E0AAAFAF	TRUE

Olanzapine_	040201060AAAAAA	1249961	Tab	Orodisper Tab	Y	Olanzapine_	040201060AA	1	040201060AAAWAW	TRUE
Olanzapine_	040201060AAACAC	1421294	Tab	Orodisper Tab	Y	Olanzapine_	040201060AA	1	040201060AAAXAX	TRUE
Olanzapine_	040201060AAAEAE	9575	Oral Lyophilisate	Orodisper Tab	Y	Olanzapine_	040201060AA	3	040201060AAASAS	TRUE
Olanzapine_	040201060AAAIAI	1485	Oral Soln	Oral Susp	Y	Olanzapine_	040201060AA	2	040201060AABABA	TRUE
Olanzapine_	040201060AAALAL	277973	Tab	Orodisper Tab	Y	Olanzapine_	040201060AA	1	040201060AAAYAY	TRUE
Olanzapine_	040201060AAAQAQ	348989	Tab	Orodisper Tab	Y	Olanzapine_	040201060AA	1	040201060AAAZAZ	TRUE
Olanzapine_	040201060AAASAS	12573	Orodisper Tab	Oral Lyophilisa	Y	Olanzapine_	040201060AA	2	040201060AAAEAE	TRUE
Olanzapine_	040201060AAAWAW	63513	Orodisper Tab	Tab	Y	Olanzapine_	040201060AA	2	040201060AAAAAA	TRUE
Olanzapine_	040201060AAAXAX	51594	Orodisper Tab	Tab	Y	Olanzapine_	040201060AA	2	040201060AAACAC	TRUE
Olanzapine_	040201060AAAYAY	17403	Orodisper Tab	Tab	Y	Olanzapine_	040201060AA	2	040201060AAALAL	TRUE
Olanzapine_	040201060AAAZAZ	28613	Orodisper Tab	Tab	Y	Olanzapine_	040201060AA	2	040201060AAAQAQ	TRUE
Olanzapine_	040201060AABABA	3180	Oral Susp	Oral Soln	Y	Olanzapine_	040201060AA	2	040201060AAAIAI	TRUE
Amisulpride_	0402010A0AAADAD	7630	Liq Spec	Oral Soln		Amisulpride_	#N/A	2	0402010A0AAAKAK	TRUE
Quetiapine_	0402010ABAAABAB	4186846	Tab	Pdr Sach		Quetiapine_	#N/A	1	0402010ABAAAQAQ	TRUE
Quetiapine_	0402010ABAAACAC	1387017	Tab	Pdr Sach		Quetiapine_	#N/A	1	0402010ABAAAPAP	TRUE
Quetiapine_	0402010ABAAAHAH	1630	Oral Soln	Oral Susp	Y	Quetiapine_	0402010ABAA	2	0402010ABAABDBD	TRUE
Quetiapine_	0402010ABAAAIAI	8395	Oral Soln	Oral Susp	Y	Quetiapine_	0402010ABAA	2	0402010ABAABBBB	TRUE
Quetiapine_	0402010ABAAALAL	1100	Oral Soln	Oral Susp	Y	Quetiapine_	0402010ABAA	2	0402010ABAABEBE	TRUE
Quetiapine_	0402010ABAAAMAM	2950	Oral Soln	Oral Susp	Y	Quetiapine_	0402010ABAA	2	0402010ABAABCBC	TRUE
Quetiapine_	0402010ABAABBBB	6140	Oral Susp	Oral Soln	Y	Quetiapine_	0402010ABAA	2	0402010ABAAAIAI	TRUE
Quetiapine_	0402010ABAABCBC	15515	Oral Susp	Oral Soln	Y	Quetiapine_	0402010ABAA	2	0402010ABAAAMAM	TRUE
Quetiapine_	0402010ABAABDBD	25030	Oral Susp	Oral Soln	Y	Quetiapine_	0402010ABAA	2	0402010ABAAAHAH	TRUE
Quetiapine_	0402010ABAABEBE	10080	Oral Susp	Oral Soln	Y	Quetiapine_	0402010ABAA	2	0402010ABAAALAL	TRUE
Chlorpromaz	0402010D0AAA2A2	1580	Liq Spec	Oral Soln	Y	Chlorpromaz	0402010D0AA	2	0402010D0AAAFAF	TRUE
Chlorpromaz	0402010D0AAAFAF	50035	Oral Soln	Liq Spec	Y	Chlorpromaz	0402010D0AA	2	0402010D0AAA2A2	TRUE
Chlorpromaz	0402010D0AAAHAH	380	Tab	Cap	Y	Chlorpromaz	0402010D0AA	1	0402010D0AABDBD	TRUE
Chlorpromaz	0402010D0AAAIAI	564271	Tab	Suppos		Chlorpromaz	#N/A	1	0402010D0AAASAS	TRUE
Chlorpromaz	0402010D0AAAJAJ	356908	Tab	Suppos		Chlorpromaz	#N/A	1	0402010D0AAATAT	TRUE
Chlorpromaz	0402010D0AAAKAK	219352	Tab	Cap	Y	Chlorpromaz	#N/A	1	0402010D0AAAYAY	TRUE
Chlorpromaz	0402010D0AAARAR	12	Suppos	Cap		Chlorpromaz	#N/A	1	0402010D0AAAYAY	TRUE
Chlorpromaz	0402010D0AABDBD	128	Cap	Tab	Y	Chlorpromaz	0402010D0AA	1	0402010D0AAAHAH	TRUE
Haloperidol_	0402010J0AAA7A7	8255	Liq Spec	Liq	Y	Haloperidol_	#N/A	2	0402010J0AAAQAQ	TRUE
Haloperidol_	0402010J0AAAAAA	362406	Cap	Tab	Y	Haloperidol_	0402010J0AA	1	0402010J0AAAIAI	TRUE
Haloperidol_	0402010J0AAAIAI	18111	Tab	Cap	Y	Haloperidol_	0402010J0AA	1	0402010J0AAAAAA	TRUE
Levomeprom	0402010K0AAARAR	2400	Oral Susp	Oral Soln		Levomeprom	#N/A	2	0402010K0AAALAL	TRUE
Promazine H	0402010S0AAADAD	1420665	Oral Soln	Liq Spec		Promazine H	#N/A	2	0402010S0AAALAL	TRUE
Promazine H	0402010S0AAAIAI	457889	Oral Soln	Liq Spec		Promazine H	#N/A	2	0402010S0AAANAN	TRUE
Sulpiride_Tal	0402010U0AAAHAH	454340	Tab	Pdrs		Sulpiride_Pdı	#N/A	1	0402010U0AAALAL	TRUE
Sulpiride_Liq	0402010U0AAANAN	840	Liq Spec	Susp		Sulpiride_Sus	#N/A	2	0402010U0AAAJAJ	TRUE
Thioridazine_	0402010W0AAACAC	120	Oral Soln	Liq Spec		Thioridazine_	#N/A	2	0402010W0AAASAS	TRUE
Lithium Carb	0402030K0AAACAC	68661	Tab	Cap	N	Lithium Carb	#N/A	1	0402030K0AAAKAK	FALSE
Lithium Carb	0402030K0AAAFAF	230017	Tab Slow	Tab	N	Lithium Carb	#N/A	2	0402030K0AAADAD	FALSE
Lithium Carb	0402030K0AAAPAP	1000	Liq Spec	Susp		Lithium Carb	#N/A	2	0402030K0AAAJAJ	TRUE
Valproic Acid	0402030Q0AAAAAA	438076	Tab G/R	Tab	Y	Valproic Acid	040801020AA	2	040801020AAADAD	TRUE
Valproic Acid	0402030Q0AAABAB	501670	Tab G/R	Cap E/C	Y	Valproic Acid	040801020AA	2	040801020AAACAC	TRUE
Amitriptyline	0403010B0AAA6A6	15748	Liq Spec	Oral Soln		Amitriptyline	#N/A	2	0403010B0AABHBH	TRUE
Amitriptyline	0403010B0AAAFAF	153651	Oral Soln	Syr		Amitriptyline	#N/A	2	0403010B0AAAWAW	TRUE
Amitriptyline	0403010B0AAAGAG	34759799	Tab	Cap	Y	Amitriptyline	#N/A	1	0403010B0AAA4A4	TRUE
Amitriptyline	0403010B0AAAHAH	11768335	Tab	Cap	Y	Amitriptyline	#N/A	1	0403010B0AAAPAP	TRUE
Amitriptyline	0403010B0AAAIAI	6574927	Tab	Cap	Y	Amitriptyline	#N/A	1	0403010B0AAASAS	TRUE
Amitriptyline	0403010B0AAANAN	433355	Oral Soln	Syr		Amitriptyline	#N/A	2	0403010B0AAAXAX	TRUE
Clomipramin	0403010F0AAAAAA	343244	Cap	Tab		Clomipramin	#N/A	1	0403010F0AAAIAI	TRUE
Clomipramin	0403010F0AAABAB	494583	Cap	Tab		Clomipramin	#N/A	1	0403010F0AAAFAF	TRUE
Dosulepin HC	0403010J0AAA6A6	650	Oral Susp	Mix		Dosulepin HC	#N/A	2	0403010J0AAAPAP	TRUE
Dosulepin HC	0403010J0AAA7A7	300	Liq Spec	Mix		Dosulepin HC	#N/A	2	0403010J0AAAEAE	TRUE
Dosulepin HC	0403010J0AAAAAA	2538624	Cap	Tab		Dosulepin HC	#N/A	1	0403010J0AAAJAJ	TRUE
Dosulepin HC	0403010J0AAAIAI	1429276	Tab	Cap	Y	Dosulepin HC	#N/A	1	0403010J0AAA2A2	TRUE
Dosulepin HC	0403010J0AABJBJ	16500	Oral Soln	Mix		Dosulepin HC	#N/A	2	0403010J0AAAPAP	TRUE
Dosulepin HC	0403010J0AABKBK	5250	Oral Soln	Liq Spec	Y	Dosulepin HC	0403010J0AA	2	0403010J0AAA7A7	TRUE
Imipramine H	0403010N0AAAEAE	815596	Tab	Cap	Y	Imipramine H	#N/A	1	0403010N0AAAAAA	TRUE
Lofepramine	0403010R0AAAGAG	300	Liq Spec	Susp		Lofepramine	#N/A	2	0403010R0AAABAB	TRUE
Nortriptyline	0403010V0AAADAD	2125620	Tab	Cap	Y	Nortriptyline	#N/A	1	0403010V0AAAAAA	TRUE
Nortriptyline	0403010V0AAAEAE	1060761	Tab	Cap	Y	Nortriptyline	#N/A	1	0403010V0AAABAB	TRUE
Nortriptyline	0403010V0AAANAN	3500	Liq Spec	Susp		Nortriptyline	#N/A	2	0403010V0AAAGAG	TRUE
Trazodone H	0403010X0AAAGAG	380	Liq Spec	Oral Liq		Trazodone H	#N/A	2	0403010X0AAACAC	TRUE
Trimipramine	0403010Y0AAAAAA	174301	Cap	Tab		Trimipramine	#N/A	1	0403010Y0AAADAD	TRUE
Trimipramine	0403010Y0AAACAC	84967	Tab	Cap	Y	Trimipramine	#N/A	1	0403010Y0AAAEAE	TRUE
Moclobemid	0403020K0AAAAAA	66667	Tab	Suppos		Moclobemid	#N/A	1	0403020K0AAABAB	TRUE
Citalopram H	0403030D0AAAAAA	23700098	Tab	Cap	Y	Citalopram H	#N/A	1	0403030D0AAALAL	TRUE
Citalopram H	0403030D0AAABAB	11125054	Tab	Cap	Y	Citalopram H	#N/A	1	0403030D0AAAKAK	TRUE
Fluoxetine H	0403030E0AAACAC	1165080	Oral Soln	Liq Spec		Fluoxetine H	#N/A	2	0403030E0AAAFAF	TRUE
Sertraline HC	0403030Q0AAAQAQ	76665	Oral Susp	Liq Spec		Sertraline HC	#N/A	2	0403030Q0AAADAD	TRUE
Sertraline HC	0403030Q0AAARAR	15305	Oral Susp	Liq Spec		Sertraline HC	#N/A	2	0403030Q0AAACAC	TRUE
Tryptophan_	0403040S0AAABAB	504	Tab	Cap	Y	Tryptophan_	0403040S0AA	1	0403040S0AAAIAI	TRUE
Tryptophan_	0403040S0AAAIAI	14006	Cap	Tab	Y	Tryptophan_	0403040S0AA	1	0403040S0AAABAB	TRUE
Venlafaxine_	0403040W0AAADAD	415409	Cap	Tab	Y	Venlafaxine_	0403040W0A	1	0403040W0AAAJAJ	TRUE
Venlafaxine_	0403040W0AAAEAE	373023	Cap	Tab	Y	Venlafaxine_	0403040W0A	1	0403040W0AAAKAK	TRUE
Venlafaxine_	0403040W0AAAJAJ	1177963	Tab	Cap	Y	Venlafaxine_	0403040W0A	1	0403040W0AAADAD	TRUE
Venlafaxine_	0403040W0AAAKAK	1270012	Tab	Cap	Y	Venlafaxine_	0403040W0A	1	0403040W0AAAEAE	TRUE
Venlafaxine_	0403040W0AAAMAM	231260	Tab	Cap	Y	Venlafaxine_	0403040W0A	1	0403040W0AAASAS	TRUE
Venlafaxine_	0403040W0AAANAN	7060	Oral Soln	Liq Spec		Venlafaxine_	#N/A	2	0403040W0AAAFAF	TRUE
Venlafaxine_	0403040W0AAAPAP	2540	Oral Susp	Liq Spec		Venlafaxine_	#N/A	2	0403040W0AAAFAF	TRUE
Venlafaxine_	0403040W0AAAQAQ	14875	Oral Soln	Liq Spec		Venlafaxine_	#N/A	2	0403040W0AAAGAG	TRUE
Venlafaxine_	0403040W0AAARAR	4600	Oral Susp	Liq Spec		Venlafaxine_	#N/A	2	0403040W0AAAGAG	TRUE

Venlafaxine_	0403040W0AAASAS	107906	Cap	Tab	Y	Venlafaxine_	0403040W0A/	1	0403040W0AAAMAM	TRUE
Mirtazapine_	0403040X0AAAAAA	4921509	Tab	Orodisper Tab	Y	Mirtazapine_	0403040X0AA	1	0403040X0AAAJAJ	TRUE
Mirtazapine_	0403040X0AAAJAJ	338547	Orodisper Tab	Tab	Y	Mirtazapine_	0403040X0AA	2	0403040X0AAAAAA	TRUE
Mirtazapine_	0403040X0AAALAL	486402	Orodisper Tab	Tab	Y	Mirtazapine_	0403040X0AA	2	0403040X0AAANAN	TRUE
Mirtazapine_	0403040X0AAAMAM	419083	Orodisper Tab	Tab	Y	Mirtazapine_	0403040X0AA	2	0403040X0AAAPAP	TRUE
Mirtazapine_	0403040X0AAANAN	5383100	Tab	Orodisper Tab	Y	Mirtazapine_	0403040X0AA	1	0403040X0AAALAL	TRUE
Mirtazapine_	0403040X0AAAPAP	3850396	Tab	Orodisper Tab	Y	Mirtazapine_	0403040X0AA	1	0403040X0AAAMAM	TRUE
Dexamfet Su	0404000L0AAAMAM	1680	Liq Spec	Elix		Dexamfet Su	#N/A	2	0404000L0AAAIAI	TRUE
Methylpheni	0404000M0AAAFAF	1740	Oral Soln	Oral Susp	Y	Methylpheni	0404000M0A/	2	0404000M0AABBBB	TRUE
Methylpheni	0404000M0AAAHAH	348	Tab	Cap	Y	Methylpheni	0404000M0A/	1	0404000M0AAAQAQ	TRUE
Methylpheni	0404000M0AAAQAQ	19921	Cap	Tab	Y	Methylpheni	0404000M0A/	1	0404000M0AAAHAH	FALSE
Methylpheni	0404000M0AAAUAU	12744	Cap	Tab	Y	Methylpheni	#N/A	1	0404000M0AAASAS	FALSE
Methylpheni	0404000M0AABBBB	5640	Oral Susp	Oral Soln	Y	Methylpheni	0404000M0A/	2	0404000M0AAAFAF	TRUE
Modafinil_O	0404000R0AAADAD	900	Oral Soln	Oral Susp	Y	Modafinil_O	0404000R0AA	2	0404000R0AAAEAE	TRUE
Modafinil_O	0404000R0AAAEAE	800	Oral Susp	Oral Soln	Y	Modafinil_O	0404000R0AA	2	0404000R0AAADAD	TRUE
Betahistine I	0406000B0AAADAD	1100	Oral Soln	Oral Susp	Y	Betahistine I	0406000B0AA	2	0406000B0AAAGAG	TRUE
Betahistine I	0406000B0AAAGAG	3450	Oral Susp	Oral Soln	Y	Betahistine I	0406000B0AA	2	0406000B0AAADAD	TRUE
Flunarizine H	0406000E0AAAAAA	3000	Cap	Tab	Y	Flunarizine H	0406000E0AA	1	0406000E0AAADAD	TRUE
Flunarizine H	0406000E0AAADAD	302	Tab	Cap	Y	Flunarizine H	0406000E0AA	1	0406000E0AAAAAA	TRUE
Cyclizine HCl	0406000F0AAACAC	4067810	Tab	Suppos		Cyclizine HCl	#N/A	1	0406000F0AAABAB	TRUE
Cyclizine HCl	0406000F0AABDBD	6460	Oral Soln	Liq Spec		Cyclizine HCl	#N/A	2	0406000F0AAAQAQ	TRUE
Cyclizine HCl	0406000F0AABEBE	17410	Oral Susp	Liq Spec		Cyclizine HCl	#N/A	2	0406000F0AAAQAQ	TRUE
Domperidon	0406000J0AAAJAJ	2743317	Tab	Suppos		Domperidon	#N/A	1	0406000J0AAAIAI	TRUE
Hyoscine Hy	0406000L0AAACAC	46152	Tab	Tab Chble	N	Hyoscine Hy	0406000L0AA	1	0406000L0AAAWAW	TRUE
Hyoscine Hy	0406000L0AAATAT	233400	Tab	Cap	Y	Hyoscine Hy	#N/A	1	0406000L0AAARAR	TRUE
Hyoscine Hy	0406000L0AAAWAW	11684	Tab Chble	Tab	Y	Hyoscine Hy	0406000L0AA	2	0406000L0AAACAC	TRUE
Hyoscine Hy	0406000L0AABMBM	15988	Oral Soln	Liq Spec		Hyoscine Hy	#N/A	2	0406000L0AAAYAY	TRUE
Hyoscine Hy	0406000L0AABNBN	16110	Oral Susp	Liq Spec		Hyoscine Hy	#N/A	2	0406000L0AAAYAY	TRUE
Hyoscine Hy	0406000L0AABPBP	1380	Oral Soln	Liq Spec		Hyoscine Hy	#N/A	2	0406000L0AAAXAX	TRUE
Hyoscine Hy	0406000L0AABQBQ	8320	Oral Susp	Liq Spec		Hyoscine Hy	#N/A	2	0406000L0AAAXAX	TRUE
Metoclopran	0406000P0AAAEAE	3690951	Tab	Suppos		Metoclopran	#N/A	1	0406000P0AAAMAM	TRUE
Ondansetron	0406000S0AAABAB	443195	Tab	Orodisper Tab	Y	Ondansetron	0406000S0AA	1	0406000S0AAAKAK	TRUE
Ondansetron	0406000S0AAACAC	64003	Tab	Orodisper Tab	Y	Ondansetron	0406000S0AA	1	0406000S0AAALAL	TRUE
Ondansetron	0406000S0AAAIAI	8627	Oral Lyophil Tab	Orodisper Film	Y	Ondansetron	0406000S0AA	3	0406000S0AAAMAM	TRUE
Ondansetron	0406000S0AAAJAJ	3020	Oral Lyophil Tab	Orodisper Film	Y	Ondansetron	0406000S0AA	3	0406000S0AAANAN	TRUE
Ondansetron	0406000S0AAAKAK	13227	Orodisper Tab	Tab	Y	Ondansetron	0406000S0AA	2	0406000S0AAABAB	TRUE
Ondansetron	0406000S0AAALAL	3228	Orodisper Tab	Tab	Y	Ondansetron	0406000S0AA	2	0406000S0AAACAC	TRUE
Ondansetron	0406000S0AAAMAM	5556	Orodisper Film	Oral Lyophil Ta	Y	Ondansetron	0406000S0AA	2	0406000S0AAAIAI	TRUE
Ondansetron	0406000S0AAANAN	1264	Orodisper Film	Oral Lyophil Ta	Y	Ondansetron	0406000S0AA	2	0406000S0AAAJAJ	TRUE
Prochlpzine I	0406000T0AAAEAE	20	Suppos	Tab	N	Prochlpzine I	0406000T0AA	1	0406000T0AAAGAG	TRUE
Prochlpzine I	0406000T0AAAGAG	5787053	Tab	Suppos	N	Prochlpzine I	0406000T0AA	1	0406000T0AAAEAE	TRUE
Ketamine_O	0406000W0AAANAN	34374	Oral Soln	Liq Spec		Ketamine_Li	#N/A	2	0406000W0AAAAAA	TRUE
Ketamine_O	0406000W0AAAPAP	9960	Oral Susp	Liq Spec		Ketamine_Li	#N/A	2	0406000W0AAAAAA	TRUE
Aspirin_Tab I	0407010B0AAA3A3	76089	Tab E/C	Cap		Aspirin_Cap	#N/A	2	0407010B0AAASAS	TRUE
Aspirin_Tab	0407010B0AAAFAF	75824	Tab	Cap	Y	Aspirin_Cap	#N/A	1	0407010B0AAASAS	TRUE
Co-Codamol_	0407010F0AAAAAA	25047235	Tab	Cap	Y	Co-Codamol_	0407010F0AA	1	0407010F0AAABAB	TRUE
Co-Codamol_	0407010F0AAABAB	1801667	Cap	Suppos		Co-Codamol_	#N/A	1	0407010F0AAANAN	TRUE
Co-Codamol_	0407010F0AAADAD	18921408	Cap	Suppos		Co-Codamol_	#N/A	1	0407010F0AAALAL	TRUE
Co-Codamol	0407010F0AAAFAF	6404873	Tab	Pdr Sach		Co-Codamol	#N/A	1	0407010F0AAAQAQ	TRUE
Co-Codamol_	0407010F0AAAHAH	49420313	Tab	Cap	Y	Co-Codamol_	0407010F0AA	1	0407010F0AAADAD	TRUE
Co-Codamol_	0407010F0AAAKAK	10577342	Tab	Cap	Y	Co-Codamol_	0407010F0AA	1	0407010F0AAAVAV	TRUE
Co-Codamol_	0407010F0AAAVAV	1822941	Cap	Tab	Y	Co-Codamol_	0407010F0AA	1	0407010F0AAAKAK	TRUE
Paracet_Oral	0407010H0AAA5A5	245820	Oral Susp	Oral Soln	Y	Paracet_Oral	0407010H0AA	2	0407010H0AADPDP	TRUE
Paracet_Oral	0407010H0AAA7A7	2902553	Oral Soln Paed	Oral Susp Paec	Y	Paracet_Oral	0407010H0AA	3	0407010H0AAAWAW	TRUE
Paracet_Cap	0407010H0AAAAAA	9990710	Cap	Capl		Paracet_Cap	0407010H0AA	1	0407010H0AAA4A4	TRUE
Paracet_Oral	0407010H0AAABAB	4540	Oral Soln Paed	Oral Susp Paec	Y	Paracet_Oral	0407010H0AA	3	0407010H0AAAIAI	TRUE
Paracet_Oral	0407010H0AAACAC	10839032	Oral Susp	Liq Spec	Y	Paracet_Liq	0407010H0AA	2	0407010H0AADBDB	TRUE
Paracet_Oral	0407010H0AAAIAI	2951636	Oral Susp Paed	Oral Soln Paed	Y	Paracet_Oral	0407010H0AA	3	0407010H0AAABAB	TRUE
Paracet_Tab	0407010H0AAAMAM	190216311	Tab	Cap	Y	Paracet_Cap	0407010H0AA	1	0407010H0AAAAAA	TRUE
Paracet_Tab	0407010H0AAAQAQ	6570278	Tab Solb	Cap	N	Paracet_Cap	0407010H0AA	2	0407010H0AAAAAA	TRUE
Paracet_Tab	0407010H0AAASAS	680	Tab Solb	Cap		Paracet_Cap	#N/A	2	0407010H0AAANAN	TRUE
Paracet_Oral	0407010H0AAAWAW	4237283	Oral Susp Paed	Oral Soln Paed	Y	Paracet_Oral	0407010H0AA	3	0407010H0AAA7A7	TRUE
Paracet_Sup	0407010H0AABNBN	860	Suppos	Pdr Sach	N	Paracet_Pdr	0407010H0AA	1	0407010H0AADGDG	TRUE
Paracet_Sup	0407010H0AABQBQ	6050	Suppos	Cap		Paracet_Cap	#N/A	1	0407010H0AAANAN	TRUE
Paracet_Sup	0407010H0AABSBS	2831	Suppos	Pdr Sach		Paracet_Pdr	#N/A	1	0407010H0AAA8A8	TRUE
Paracet_Sup	0407010H0AABUBU	12923	Suppos	Cap	N	Paracet_Cap	0407010H0AA	1	0407010H0AAAAAA	TRUE
Paracet_Sup	0407010H0AACBCB	2137	Suppos	Cap		Paracet_Cap	#N/A	1	0407010H0AADADA	TRUE
Paracet_Sup	0407010H0AACMCM	5296	Suppos	Cap		Paracet_Cap	#N/A	1	0407010H0AACQCQ	TRUE
Paracet_Liq	0407010H0AACPCP	8930	Liq Spec	Elix		Paracet_Elix	#N/A	2	0407010H0AAA3A3	TRUE
Paracet_Liq	0407010H0AADBDB	6000	Liq Spec	Oral Susp	Y	Paracet_Oral	0407010H0AA	2	0407010H0AAACAC	TRUE
Paracet_Rap	0407010H0AADCDC	20007	Rapid Tab	Cap		Paracet_Cap	#N/A	2	0407010H0AADADA	TRUE
Paracet_Pdr	0407010H0AADGDG	50	Pdr Sach	Pdrs		Paracet_Pdrs	#N/A	2	0407010H0AAAYAY	TRUE
Paracet_Tab	0407010H0AADLDL	1389	Tab	Pdr Sach	N	Paracet_Pdr	0407010H0AA	1	0407010H0AADGDG	TRUE
Paracet_Oral	0407010H0AADPDP	570677	Oral Soln	Oral Susp	Y	Paracet_Oral	0407010H0AA	2	0407010H0AAA5A5	TRUE
Co-Dydramo	0407010N0AAAAAA	20364757	Tab	Pdr Sach		Co-Dydramo	#N/A	1	0407010N0AAAFAF	TRUE
Co-Dydramo	0407010N0AAACAC	1400	Oral Soln	Oral Susp	Y	Co-Dydramo	0407010N0AA	2	0407010N0AAAGAG	TRUE
Co-Dydramo	0407010N0AAAGAG	1950	Oral Susp	Oral Soln	Y	Co-Dydramo	0407010N0AA	2	0407010N0AAACAC	TRUE
Tramadol HC	040702040AAAAAA	52885881	Cap	Eff Pdr Sach		Tramadol HC	#N/A	1	040702040AAAKAK	TRUE
Tramadol HC	040702040AAACAC	885970	Tab	Cap	Y	Tramadol HC	040702040AA	1	040702040AAAHAH	TRUE
Tramadol HC	040702040AAADAD	89805	Tab	Cap	Y	Tramadol HC	040702040AA	1	040702040AAAIAI	TRUE
Tramadol HC	040702040AAAEAE	241319	Tab	Cap	Y	Tramadol HC	040702040AA	1	040702040AAAJAJ	TRUE
Tramadol HC	040702040AAAFAF	161165	Tab Solb	Orodisper Tab	Y	Tramadol HC	040702040AA	2	040702040AAATAT	TRUE
Tramadol HC	040702040AAAGAG	549400	Cap	Tab	Y	Tramadol HC	040702040AA	1	040702040AAAYAY	TRUE
Tramadol HC	040702040AAAHAH	1023997	Cap	Tab	Y	Tramadol HC	040702040AA	1	040702040AAACAC	TRUE

Tramadol HC	040702040AAAIAI	159142	Cap	Tab	Y	Tramadol HC	040702040AA	1	040702040AAADAD	TRUE
Tramadol HC	040702040AAAJAJ	387664	Cap	Tab	Y	Tramadol HC	040702040AA	1	040702040AAAEAE	TRUE
Tramadol HC	040702040AAATAT	55666	Orodisper Tab	Tab Solb	Y	Tramadol HC	040702040AA	2	040702040AAAFAF	TRUE
Tramadol HC	040702040AAAYAY	356271	Tab	Cap	Y	Tramadol HC	040702040AA	1	040702040AAAGAG	TRUE
Fentanyl_Tal	0407020A0AAAWAW	5713	Tab Sublingual	Tab Buccal	Y	Fentanyl_Tal	0407020A0AA	2	0407020A0AABCBC	TRUE
Fentanyl_Tal	0407020A0AAAXAX	6180	Tab Sublingual	Buccal Film		Fentanyl_Bu	#N/A	2	0407020A0AABTBT	TRUE
Fentanyl_Tal	0407020A0AAAZAZ	2586	Tab Sublingual	Buccal Film		Fentanyl_Bu	#N/A	2	0407020A0AABUBU	TRUE
Fentanyl_Tal	0407020A0AABABA	576	Tab Sublingual	Tab Buccal	Y	Fentanyl_Tal	0407020A0AA	2	0407020A0AABFBF	TRUE
Fentanyl_Tal	0407020A0AABBBB	993	Tab Sublingual	Buccal Film		Fentanyl_Bu	#N/A	2	0407020A0AABVBV	TRUE
Fentanyl_Tal	0407020A0AABCBC	8776	Tab Buccal	Tab Sublingual	Y	Fentanyl_Tal	0407020A0AA	2	0407020A0AAAWAW	TRUE
Fentanyl_Tal	0407020A0AABDBD	9545	Tab Buccal	Buccal Film		Fentanyl_Bu	#N/A	2	0407020A0AABTBT	TRUE
Fentanyl_Tal	0407020A0AABEBE	6813	Tab Buccal	Buccal Film		Fentanyl_Bu	#N/A	2	0407020A0AABUBU	TRUE
Fentanyl_Tal	0407020A0AABFBF	1430	Tab Buccal	Tab Sublingual	Y	Fentanyl_Tal	0407020A0AA	2	0407020A0AABABA	TRUE
Fentanyl_Tal	0407020A0AABGBG	2016	Tab Buccal	Buccal Film		Fentanyl_Bu	#N/A	2	0407020A0AABVBV	TRUE
Codeine Pho	0407020C0AAADAD	10847338	Tab	Cap	Y	Codeine Pho	#N/A	1	0407020C0AAAJAJ	TRUE
Codeine Pho	0407020C0AAAEAE	22397747	Tab	Cap	Y	Codeine Pho	#N/A	1	0407020C0AAAUAU	TRUE
Codeine Pho	0407020C0AAASAS	12	Suppos	Cap		Codeine Pho	#N/A	1	0407020C0AAAUAU	TRUE
Dihydrocode	0407020G0AAAAAA	107925	Oral Soln	Liq Spec		Dihydrocode	#N/A	2	0407020G0AAAPAP	TRUE
Dihydrocode	0407020G0AAACAC	11690805	Tab	Cap	Y	Dihydrocode	#N/A	1	0407020G0AAAQAQ	TRUE
Diamorph HC	0407020K0AACBCB	16303	Tab	Reefer		Diamorph HC	#N/A	1	0407020K0AABYBY	TRUE
Diamorph HC	0407020K0AADCDC	52	Reefer	Cap		Diamorph HC	#N/A	1	0407020K0AAETET	TRUE
Diamorph HC	0407020K0AADIDI	300	Liq Spec	Linct		Diamorph HC	#N/A	2	0309010N0AAACAC	TRUE
Diamorph HC	0407020K0AAEUEU	52	Reefer	Suppos		Diamorph HC	#N/A	1	0407020K0AACJCJ	TRUE
Methadone I	0407020M0AAAEAE	349219	Tab	Cap	Y	Methadone I	#N/A	1	0407020M0AABUBU	TRUE
Methadone I	0407020M0AABIBI	511	Cap	Reefer		Methadone I	#N/A	1	0407020M0AAAJAJ	TRUE
Methadone I	0407020M0AABLBL	200	Cap	Reefer		Methadone I	#N/A	1	0407020M0AAA1A1	TRUE
Methadone I	0407020M0AABMBM	336	Cap	Reefer		Methadone I	#N/A	1	0407020M0AAA2A2	TRUE
Morph Sulf_I	0407020Q0AAA4A4	10	Inj	Epidural Inj		Morph Sulf_I	#N/A	1	0407020Q0AAEQEQ	TRUE
Morph Sulf_I	0407020Q0AAA9A9	25	Inj	Epidural Inj		Morph Sulf_I	#N/A	1	0407020Q0AACICI	TRUE
Morph Sulf_I	0407020Q0AAABAB	81679	Inj	Epidural Inj		Morph Sulf_I	#N/A	1	0407020Q0AACJCJ	TRUE
Morph Sulf_I	0407020Q0AAACAC	8418	Inj	Epidural Inj		Morph Sulf_I	#N/A	1	0407020Q0AAEMEM	TRUE
Morph Sulf_I	0407020Q0AAADAD	7311	Inj	Epidural Inj		Morph Sulf_I	#N/A	1	0407020Q0AACXCX	TRUE
Morph Sulf_	0407020Q0AAAGAG	4586	Tab	Cap	Y	Morph Sulf_(	0407020Q0AA	1	0407020Q0AAEIEI	TRUE
Morph Sulf_	0407020Q0AAAHAH	36596	Tab	Cap	Y	Morph Sulf_(	0407020Q0AA	1	0407020Q0AAEBEB	TRUE
Morph Sulf_	0407020Q0AAAIAI	149946	Tab	Cap	Y	Morph Sulf_(	0407020Q0AA	1	0407020Q0AAEHEH	TRUE
Morph Sulf_	0407020Q0AAAKAK	976217	Tab	Cap	Y	Morph Sulf_(	0407020Q0AA	1	0407020Q0AAEFEF	TRUE
Morph Sulf_	0407020Q0AAALAL	494098	Tab	Cap	Y	Morph Sulf_(	0407020Q0AA	1	0407020Q0AAEGEG	TRUE
Morph Sulf_S	0407020Q0AABMBM	144	Suppos	Cap		Morph Sulf_(	#N/A	1	0407020Q0AADSDS	TRUE
Morph Sulf_	0407020Q0AACDCD	356949	Tab	Suppos	N	Morph Sulf_S	0407020Q0AA	1	0407020Q0AACQCQ	TRUE
Morph Sulf_	0407020Q0AACECE	114087	Tab	Suppos		Morph Sulf_S	#N/A	1	0407020Q0AACRCR	TRUE
Morph Sulf_	0407020Q0AACFCF	141103	Tab	Gran Sach		Morph Sulf_(	#N/A	1	0407020Q0AAFLFL	TRUE
Morph Sulf_(	0407020Q0AACNCN	32164535	Oral Soln	Liq Spec		Morph Sulf_I	#N/A	2	0407020Q0AAEKEK	TRUE
Morph Sulf_(	0407020Q0AACPCP	8256	Gran Sach	Cap	N	Morph Sulf_(	0407020Q0AA	2	0407020Q0AAEGEG	TRUE
Morph Sulf_S	0407020Q0AACQCQ	844	Suppos	Tab	N	Morph Sulf_	0407020Q0AA	1	0407020Q0AACDCD	TRUE
Morph Sulf_(	0407020Q0AACVCV	12552	Gran Sach	Cap		Morph Sulf_(	#N/A	2	0407020Q0AADZDZ	TRUE
Morph Sulf_(	0407020Q0AADCDC	2931	Gran Sach	Cap	Y	Morph Sulf_(	0407020Q0AA	2	0407020Q0AAEHEH	TRUE
Morph Sulf_(	0407020Q0AADDDD	1386	Gran Sach	Cap	Y	Morph Sulf_(	0407020Q0AA	2	0407020Q0AAEBEB	TRUE
Morph Sulf_(	0407020Q0AADEDE	60	Gran Sach	Cap	Y	Morph Sulf_(	0407020Q0AA	2	0407020Q0AAEIEI	TRUE
Morph Sulf_I	0407020Q0AADNDN	100	Liq Spec	Oral Soln		Morph Sulf_(	#N/A	2	0407020Q0AAASAS	TRUE
Morph Sulf_	0407020Q0AADRDR	24068	Tab	Suppos		Morph Sulf_S	#N/A	1	0407020Q0AABVBV	TRUE
Morph Sulf_(	0407020Q0AAEBEB	28760	Cap	Gran Sach	N	Morph Sulf_(	0407020Q0AA	1	0407020Q0AADDDD	TRUE
Morph Sulf_(	0407020Q0AAEFEF	796572	Cap	Tab	Y	Morph Sulf_	0407020Q0AA	1	0407020Q0AAAKAK	TRUE
Morph Sulf_(	0407020Q0AAEGEG	302957	Cap	Gran Sach	N	Morph Sulf_(	0407020Q0AA	1	0407020Q0AACPCP	FALSE
Morph Sulf_(	0407020Q0AAEHEH	97063	Cap	Gran Sach	N	Morph Sulf_(	0407020Q0AA	1	0407020Q0AADCDC	FALSE
Morph Sulf_(	0407020Q0AAEIEI	5934	Cap	Gran Sach	N	Morph Sulf_(	0407020Q0AA	1	0407020Q0AADEDE	FALSE
Morph Sulf_I	0407020Q0AAFXFX	722	Intrasite Gel	Gel		Morph Sulf_(	#N/A	2	0407020Q0AAFSFS	TRUE
Morph Sulf_I	0407020Q0AAFYFY	680	Intrasite Gel	Gel		Morph Sulf_(	#N/A	2	0407020Q0AAFUFU	TRUE
Pethidine HC	0407020V0AAACAC	148040	Tab	Cap	Y	Pethidine HC	#N/A	1	0407020V0AABFBF	TRUE
Rizatriptan_T	0407041R0AAABAB	40888	Tab	Oral Lyophilisa	Y	Rizatriptan_(	0407041R0AA	1	0407041R0AAACAC	TRUE
Rizatriptan_(	0407041R0AAACAC	82836	Oral Lyophilisate	Tab	Y	Rizatriptan_T	0407041R0AA	3	0407041R0AAABAB	TRUE
Tolfenamic A	0407041U0AAABAB	8123	Tab	Cap		Tolfenamic A	#N/A	1	0407041U0AAAAAA	TRUE
Clonidine HC	0407042F0AAAGAG	7300	Liq Spec	Soln		Clonidine HC	#N/A	2	0407042F0AAABAB	TRUE
Clonidine HC	0407042F0AAATAT	64195	Oral Soln	Liq Spec		Clonidine HC	#N/A	2	0407042F0AAAFAF	TRUE
Clonidine HC	0407042F0AAAUAU	27448	Oral Susp	Liq Spec		Clonidine HC	#N/A	2	0407042F0AAAFAF	TRUE
Valproic Acid	040801020AAACAC	53752	Cap E/C	Tab		Valproic Acid	#N/A	2	040801020AAAEAE	TRUE
Valproic Acid	040801020AAADAD	56	Tab	Tab G/R	Y	Valproic Acid	0402030Q0AA	1	0402030Q0AAAAAA	TRUE
Topiramate_	040801050AAAAAA	1153308	Tab	Cap	Y	Topiramate_	040801050AA	1	040801050AAAWAW	TRUE
Topiramate_	040801050AAABAB	750071	Tab	Cap	Y	Topiramate_	#N/A	1	040801050AAANAN	TRUE
Topiramate_	040801050AAACAC	153572	Tab	Cap	Y	Topiramate_	#N/A	1	040801050AABQBQ	TRUE
Topiramate_	040801050AAADAD	1685987	Tab	Cap	Y	Topiramate_	040801050AA	1	040801050AAAVAV	TRUE
Topiramate_	040801050AAAUAU	99431	Cap	Pdrs		Topiramate_	#N/A	1	040801050AABBBB	TRUE
Topiramate_	040801050AAAVAV	265231	Cap	Pdrs		Topiramate_	#N/A	1	040801050AAAIAI	TRUE
Topiramate_	040801050AAAWAW	154642	Cap	Pdrs		Topiramate_	#N/A	1	040801050AAAKAK	TRUE
Topiramate_	040801050AABXBX	93882	Oral Susp	Liq Spec		Topiramate_	#N/A	2	040801050AAALAL	TRUE
Topiramate_	040801050AABYBY	50271	Oral Susp	Liq Spec		Topiramate_	#N/A	2	040801050AAARAR	TRUE
Clobazam_Li	040801060AAA1A1	5700	Liq Spec	Oral Soln		Clobazam_O	#N/A	2	040801060AACPCP	TRUE
Clobazam_Li	040801060AAA2A2	400	Liq Spec	Susp		Clobazam_Su	#N/A	2	040801060AAALAL	TRUE
Clobazam_Li	040801060AAA3A3	2300	Liq Spec	Susp		Clobazam_Su	#N/A	2	040801060AAAPAP	TRUE
Clobazam_Li	040801060AAA4A4	6375	Liq Spec	Oral Soln		Clobazam_O	#N/A	2	040801060AACMCM	TRUE
Clobazam_Li	040801060AABABA	5140	Liq Spec	Susp		Clobazam_Su	#N/A	2	040801060AAAKAK	TRUE
Clobazam_Ta	040801060AABTBT	919269	Tab	Cap	Y	Clobazam_Ca	#N/A	1	040801060AAAAAA	TRUE
Clobazam_Ta	040801060AACKCK	6817	Tab	Cap		Clobazam_Ca	#N/A	1	040801060AABVBV	TRUE
Zonisamide_	0408010ADAAADAD	9150	Oral Soln	Oral Susp	Y	Zonisamide_	0408010ADAA	2	0408010ADAAAEAE	TRUE
Zonisamide_	0408010ADAAAEAE	31620	Oral Susp	Oral Soln	Y	Zonisamide_	0408010ADAA	2	0408010ADAAADAD	TRUE

Pregabalin_C	0408010AEAAACAC	3322473	Cap	Pdr Sach		Pregabalin_P	#N/A	1	0408010AEAAALAL	TRUE
Pregabalin_C	0408010AEAAAHAH	10433	Oral Soln	Oral Susp		Pregabalin_C	#N/A	2	0408010AEAAAPAP	TRUE
Stiripentol_C	0408010AGAAAAAA	2468	Cap	Pdr Sach	N	Stiripentol_P	0408010AGA/	1	0408010AGAAACAC	TRUE
Stiripentol_C	0408010AGAAABAB	2356	Cap	Pdr Sach	Y	Stiripentol_P	0408010AGA/	1	0408010AGAAADAD	TRUE
Stiripentol_P	0408010AGAAACAC	7666	Pdr Sach	Cap	Y	Stiripentol_C	0408010AGA/	2	0408010AGAAAAAA	TRUE
Stiripentol_P	0408010AGAAADAD	4346	Pdr Sach	Cap	N	Stiripentol_C	0408010AGA/	2	0408010AGAAABAB	TRUE
Carbamazepi	0408010C0AAABAB	1761731	Tab	Suppos	N	Carbamazepi	#N/A	1	0408010C0AAAFAF	FALSE
Carbamazepi	0408010C0AAACAC	1713302	Tab	Tab Chble	N	Carbamazepi	0408010C0AA	1	0408010C0AAAKAK	FALSE
Carbamazepi	0408010C0AAAJAJ	168	Tab Chble	Suppos		Carbamazepi	#N/A	2	0408010C0AAAFAF	TRUE
Carbamazepi	0408010C0AAAKAK	56	Tab Chble	Tab	Y	Carbamazepi	0408010C0AA	2	0408010C0AAACAC	TRUE
Clonazepam_	0408010F0AAABAB	2959740	Tab	Orodisper Tab		Clonazepam_	#N/A	1	0408010F0AACZCZ	TRUE
Clonazepam_	0408010F0AABCBC	2991	Liq Spec	Susp		Clonazepam_	#N/A	2	0408010F0AAARAR	TRUE
Clonazepam_	0408010F0AABDBD	360	Liq Spec	Susp		Clonazepam_	#N/A	2	0408010F0AAAYAY	TRUE
Clonazepam_	0408010F0AABEBE	12975	Liq Spec	Elix		Clonazepam_	#N/A	2	0408010F0AAAMAM	TRUE
Clonazepam_	0408010F0AABMBM	1600	Liq Spec	Syr		Clonazepam_	#N/A	2	0408010F0AAAHAH	TRUE
Clonazepam_	0408010F0AABPBP	50	Liq Spec	Syr		Clonazepam_	#N/A	2	0408010F0AAADAD	TRUE
Clonazepam_	0408010F0AACACA	50	Liq Spec	Susp		Clonazepam_	#N/A	2	0408010F0AAAZAZ	TRUE
Clonazepam_	0408010F0AACECE	1800	Liq Spec	Susp		Clonazepam_	#N/A	2	0408010F0AAAWAW	TRUE
Gabapentin_	0408010G0AAACAC	2651980	Cap	Pdrs		Gabapentin_	#N/A	1	0408010G0AAAFAF	TRUE
Gabapentin_	0408010G0AAAQAQ	37310	Liq Spec	Oral Soln	Y	Gabapentin_	0408010G0AA	2	0408010G0AAATAT	TRUE
Gabapentin_	0408010G0AAATAT	2958	Oral Soln	Liq Spec	Y	Gabapentin_	0408010G0AA	2	0408010G0AAAQAQ	TRUE
Gabapentin_	0408010G0AAAYAY	16685	Liq Spec	Oral Soln		Gabapentin_	#N/A	2	0408010G0AABEBE	TRUE
Lamotrigine_	0408010H0AAA1A1	1040624	Tab	Tab Disper	N	Lamotrigine_	#N/A	1	0408010H0AABQBQ	TRUE
Lamotrigine_	0408010H0AAAAAA	3511660	Tab	Cap	Y	Lamotrigine_	#N/A	1	0408010H0AABFBF	TRUE
Lamotrigine_	0408010H0AAABAB	2923099	Tab	Suppos		Lamotrigine_	#N/A	1	0408010H0AABABA	TRUE
Lamotrigine_	0408010H0AAACAC	2239523	Tab	Pdrs		Lamotrigine_	#N/A	1	0408010H0AAAUAU	TRUE
Ethosuximide	0408010I0AAAAAA	195062	Cap	Pdrs		Ethosuximide	#N/A	1	0408010I0AAAGAG	TRUE
Ethosuximide	0408010I0AAABAB	398854	Oral Soln	Liq Spec		Ethosuximide	#N/A	2	0408010I0AAAIAI	TRUE
Phenobarb_E	0408010N0AAACAC	153305	Elix	Liq		Phenobarb_L	#N/A	1	0408010N0AAAUAU	TRUE
Phenobarb_T	0408010N0AAAIAI	105547	Tab	Cap	N	Phenobarb_C	#N/A	1	0408010N0AACJCJ	FALSE
Phenobarb_T	0408010N0AAAJAJ	766038	Tab	Cap	N	Phenobarb_C	#N/A	1	0408010N0AAARAR	FALSE
Phenobarb_T	0408010N0AAALAL	254780	Tab	Cap	N	Phenobarb_C	#N/A	1	0408010N0AAAVAV	FALSE
Phenobarb_C	0408010N0AAASAS	100	Cap	Tab		Phenobarb_T	#N/A	1	0408010N0AAAMAM	TRUE
Phenobarb_L	0408010N0AACLCL	90934	Liq Spec	Elix		Phenobarb_E	#N/A	2	0408010N0AABQBQ	TRUE
Phenobarb_L	0408010N0AACPCP	250	Liq Spec	Elix		Phenobarb_E	#N/A	2	0408010N0AABNBN	TRUE
Phenobarb_L	0408010N0AACTCT	200	Liq Spec	Elix		Phenobarb_E	#N/A	2	0408010N0AAAPAP	TRUE
Phenobarb_L	0408010N0AACUCU	650	Liq Spec	Elix		Phenobarb_E	#N/A	2	0408010N0AAA8A8	TRUE
Phenobarb_L	0408010N0AACWCW	4220	Liq Spec	Elix		Phenobarb_E	#N/A	2	0408010N0AAA5A5	TRUE
Phenobarb_L	0408010N0AACXCX	640	Liq Spec	Elix		Phenobarb_E	#N/A	2	0408010N0AABMBM	TRUE
Phenobarb_L	0408010N0AACYCY	3300	Liq Spec	Elix		Phenobarb_E	#N/A	2	0408010N0AABPBP	TRUE
Phenobarb_L	0408010N0AADIDI	1300	Liq Spec	Soln		Phenobarb_S	#N/A	2	0408010N0AAERER	TRUE
Phenobarb_L	0408010N0AADMDM	7600	Liq Spec	Elix	Y	Phenobarb_E	0408010N0AA	2	0408010N0AAACAC	TRUE
Phenobarb S	0408010P0AAAWAW	450	Liq Spec	Soln		Phenobarb S	#N/A	2	0408010P0AAANAN	TRUE
Phenobarb S	0408010P0AAAYAY	600	Liq Spec	Elix		Phenobarb S	#N/A	2	0408010P0AAAFAF	TRUE
Phenytoin_S	0408010Q0AAAAAA	2382831	Sod Cap	Sod Clear Cap	N	Phenytoin_S	#N/A	2	0408010Q0AAARAR	FALSE
Phenytoin_S	0408010Q0AAADAD	203904	Sod Cap	Suppos	N	Phenytoin_S	#N/A	2	0408010Z0AAATAT	FALSE
Phenytoin_S	0408010Q0AAAGAG	599189	Sod Tab	Sod Cap	N	Phenytoin_S	0408010Q0AA	2	0408010Q0AAAAAA	FALSE
Phenytoin_S	0408010Q0AAAPAP	420576	Sod Cap	Sod Clear Cap	N	Phenytoin_S	#N/A	2	0408010Q0AAASAS	FALSE
Phenytoin_S	0408010Q0AAAYAY	8062	Sod Oral Soln	Oral Susp	Y	Phenytoin_O	0408010Z0AA	3	0408010Z0AAADAD	TRUE
Primidone_C	0408010U0AAACAC	275	Oral Susp	Liq Spec		Primidone_Li	#N/A	2	0408010U0AAALAL	TRUE
Primidone_T	0408010U0AAAXAX	312602	Tab	Cap	Y	Primidone_C	#N/A	1	0408010U0AAAFAF	TRUE
Sod Valpr_Ta	0408010W0AAA1A1	542735	Tab	Cap	Y	Sod Valpr_Ca	0408010W0A/	1	0408010W0AABRBR	TRUE
Sod Valpr_Oı	0408010W0AAAAAA	7496679	Oral Soln	Syr		Sod Valpr_Sy	#N/A	2	0408010W0AAAXAX	TRUE
Sod Valpr_Ta	0408010W0AAABAB	521613	Tab	Cap	Y	Sod Valpr_Ca	#N/A	1	0408010W0AAANAN	TRUE
Sod Valpr_Ta	0408010W0AAACAC	2017294	Tab E/C	Cap		Sod Valpr_Ca	#N/A	2	0408010W0AAA8A8	TRUE
Sod Valpr_Ta	0408010W0AAADAD	1143466	Tab E/C	Cap		Sod Valpr_Ca	#N/A	2	0408010W0AAAFAF	TRUE
Sod Valpr_Oı	0408010W0AAAEAE	2062098	Oral Soln	Liq Spec		Sod Valpr_Liı	#N/A	2	0408010W0AABABA	TRUE
Sod Valpr_Sı	0408010W0AABCBC	36	Suppos	Cap		Sod Valpr_Ca	#N/A	1	0408010W0AAAPAP	TRUE
Sod Valpr_Ca	0408010W0AABRBR	242100	Cap	Tab	N	Sod Valpr_Ta	0408010W0A/	1	0408010W0AAA1A1	TRUE
Vigabatrin_T	0408010X0AAAAAA	56585	Tab	Pdrs		Vigabatrin_P	#N/A	1	0408010X0AAAQAQ	TRUE
Phenytoin_Ta	0408010Z0AAACAC	20617	Tab Chble	Sod Cap	N	Phenytoin_S	0408010Q0AA	2	0408010Q0AAAPAP	TRUE
Phenytoin_O	0408010Z0AAALAL	61970	Oral Susp	Sod Oral Soln	Y	Phenytoin_S	0408010Q0AA	2	0408010Q0AAAYAY	TRUE
Midazolam_(	0408020V0AAAPAP	5	Oromuc Soln	Liq Spec Oromucosal		Midazolam_I	#N/A	2	0408020V0AAAAAA	TRUE
Ropinirole H	0409010H0AAABAB	495985	Tab	Pdr Sach		Ropinirole H	#N/A	1	0409010H0AAAJAJ	TRUE
Co-Beneldop	0409010K0AAAKAK	128365	Cap	Tab		Co-Beneldop	#N/A	1	0409010K0AAAGAG	TRUE
Co-Careldopa	0409010N0AAAAAA	56035	Tab	Cap	Y	Co-Careldopa	#N/A	1	0409010N0AAALAL	TRUE
Co-Careldopa	0409010N0AAAKAK	9410	Oral Soln	Oral Susp	Y	Co-Careldopa	0409010N0AA	2	0409010N0AAAUAU	TRUE
Co-Careldopa	0409010N0AAAUAU	16780	Oral Susp	Oral Soln	Y	Co-Careldopa	0409010N0AA	2	0409010N0AAAKAK	TRUE
Co-Careldopa	0409010N0AAAVAV	11650	Oral Susp	Liq Spec		Co-Careldopa	#N/A	2	0409010N0AAAMAM	TRUE
Pergolide Me	0409010P0AAACAC	2222	Tab	Pdrs		Pergolide Me	#N/A	1	0409010P0AAAFAF	TRUE
Entacapone_	0409010V0AAAAAA	358034	Tab	Pdrs		Entacapone_	#N/A	1	0409010V0AAADAD	TRUE
Trihexypheni	0409020C0AAACAC	246548	Oral Soln	Liq Spec	Y	Trihexypheni	0409020C0AA	2	0409020C0AAAKAK	TRUE
Trihexypheni	0409020C0AAAKAK	100	Liq Spec	Oral Soln	Y	Trihexypheni	0409020C0AA	2	0409020C0AAACAC	TRUE
Trihexypheni	0409020C0AAALAL	14800	Liq Spec	Oral Soln		Trihexypheni	#N/A	2	0409020C0AAAMAM	TRUE
Tetrabenazin	0409030C0AAAGAG	450	Liq Spec	Susp		Tetrabenazin	#N/A	2	0409030C0AAABAB	TRUE
Tetrabenazin	0409030C0AAARAR	13880	Oral Susp	Liq Spec		Tetrabenazin	#N/A	2	0409030C0AAAFAF	TRUE
Tetrabenazin	0409030C0AAASAS	8570	Oral Susp	Liq Spec		Tetrabenazin	#N/A	2	0409030C0AAAIAI	TRUE
Riluzole_Tab	0409030R0AAAAAA	85791	Tab	Pdrs		Riluzole_Pdrs	#N/A	1	0409030R0AAABAB	TRUE
Nicotine_Inh	0410020B0AAAVAV	210	Inhalator + Inh Ca	Skin Patch		Nicotine_Skiı	#N/A	4	0410020B0AAALAL	TRUE
Nicotine_Sut	0410020B0AAAWAW	21405	Subling Tab	Chewing Gum	N	Nicotine_Che	0410020B0AA	2	0410020B0AABABA	TRUE
Nicotine_Loz	0410020B0AAAYAY	83585	Loz	Chewing Gum	N	Nicotine_Che	0410020B0AA	1	0410020B0AABABA	TRUE
Nicotine_Loz	0410020B0AAAZAZ	43376	Loz	Chewing Gum	Y	Nicotine_Che	0410020B0AA	1	0410020B0AABDBD	TRUE
Nicotine_Che	0410020B0AABABA	127516	Chewing Gum	Loz	N	Nicotine_Loz	0410020B0AA	2	0410020B0AAAYAY	TRUE
Nicotine_Che	0410020B0AABDBD	104976	Chewing Gum	Loz	N	Nicotine_Loz	0410020B0AA	2	0410020B0AAAZAZ	TRUE

Nicotine_Inh	0410020B0AABZBZ	214504	Inhalator + Inh Ca	Skin Patch		Nicotine_Skin	#N/A	4	0410020B0AAAMAM	TRUE
Naltrexone H	0410030E0AAATAT	850	Oral Susp	Oral Soln		Naltrexone H	#N/A	2	0410030E0AAARAR	TRUE
Donepezil HC	0411000D0AAAAAA	861611	Tab	Orodisper Tab	Y	Donepezil HC	0411000D0AA	1	0411000D0AAAHAH	TRUE
Donepezil HC	0411000D0AAABAB	2443665	Tab	Orodisper Tab	Y	Donepezil HC	0411000D0AA	1	0411000D0AAAIAI	TRUE
Donepezil HC	0411000D0AAAHAH	2367	Orodisper Tab	Tab	Y	Donepezil HC	0411000D0AA	2	0411000D0AAAAAA	TRUE
Donepezil HC	0411000D0AAAIAI	5888	Orodisper Tab	Tab	Y	Donepezil HC	0411000D0AA	2	0411000D0AAABAB	TRUE
Phenoxymetl	0501011P0AAADAD	2676700	Soln	Susp		Phenoxymetl	#N/A	1	0501011P0AAAHAH	TRUE
Phenoxymetl	0501011P0AAAFAF	2430800	Soln	Susp		Phenoxymetl	#N/A	1	0501011P0AAAQAQ	TRUE
Fluclox Sod_	0501012G0AAAFAF	3104600	Oral Soln	Oral Susp		Fluclox Sod_	#N/A	2	0501012G0AAAHAH	TRUE
Fluclox Sod_	0501012G0AAAPAP	331100	Oral Soln	Mix		Fluclox Sod_	#N/A	2	0501012G0AAALAL	TRUE
Amoxicillin_(	0501013B0AAAAAA	637176	Cap	Tab		Amoxicillin_T	#N/A	1	0501013B0AAA4A4	TRUE
Amoxicillin_(	0501013B0AAABAB	8293873	Cap	Tab		Amoxicillin_T	#N/A	1	0501013B0AAA5A5	TRUE
Ceftazidime I	0501021H0AAACAC	91	Inj	Inf		Ceftazidime I	#N/A	1	0501021H0AAAEAE	TRUE
Cefuroxime A	0501021K0AAAAAA	1043	Tab	Gran Sach		Cefuroxime A	#N/A	1	0501021K0AAADAD	TRUE
Cefalexin_Ca	0501021L0AAAAAA	671557	Cap	Tab	Y	Cefalexin_Ta	0501021L0AA	1	0501021L0AAAGAG	TRUE
Cefalexin_Ca	0501021L0AAABAB	549602	Cap	Tab	Y	Cefalexin_Ta	0501021L0AA	1	0501021L0AAAHAH	TRUE
Cefalexin_Ta	0501021L0AAAGAG	178355	Tab	Cap	Y	Cefalexin_Ca	0501021L0AA	1	0501021L0AAAAAA	TRUE
Cefalexin_Ta	0501021L0AAAHAH	87732	Tab	Cap	Y	Cefalexin_Ca	0501021L0AA	1	0501021L0AAABAB	TRUE
Demeclocycl	0501030F0AAAAAA	28396	Cap	Tab	Y	Demeclocycl	0501030F0AA	1	0501030F0AAAIAI	TRUE
Demeclocycl	0501030F0AAAIAI	1629	Tab	Cap	Y	Demeclocycl	0501030F0AA	1	0501030F0AAAAAA	TRUE
Doxycycline I	0501030I0AAABAB	2629847	Cap	Pdrs		Doxycycline I	#N/A	1	0501030I0AAAFAF	TRUE
Doxycycline I	0501030I0AAAHAH	200	Liq Spec	Syr		Doxycycline I	#N/A	2	0501030I0AAACAC	TRUE
Minocycline	0501030P0AAAAAA	18168	Tab	Cap	Y	Minocycline	0501030P0AA	1	0501030P0AAADAD	TRUE
Minocycline	0501030P0AAABAB	33187	Tab	Cap	Y	Minocycline	0501030P0AA	1	0501030P0AAAEAE	TRUE
Minocycline	0501030P0AAADAD	7760	Cap	Tab	Y	Minocycline	0501030P0AA	1	0501030P0AAAAAA	TRUE
Minocycline	0501030P0AAAEAE	27712	Cap	Tab	Y	Minocycline	0501030P0AA	1	0501030P0AAABAB	TRUE
Oxytetracycli	0501030T0AAAJAJ	4236745	Tab	Cap	Y	Oxytetracycli	#N/A	1	0501030T0AAAAAA	TRUE
Tetracycline_	0501030V0AAAAAA	84	Cap	Tab	Y	Tetracycline_	0501030V0AA	1	0501030V0AAAFAF	TRUE
Tetracycline_	0501030V0AAAFAF	231038	Tab	Cap	Y	Tetracycline_	0501030V0AA	1	0501030V0AAAAAA	TRUE
Azithromycin	0501050A0AAAAAA	114584	Cap	Tab	Y	Azithromycin	0501050A0AA	1	0501050A0AAAGAG	TRUE
Azithromycin	0501050A0AAAGAG	430104	Tab	Cap	Y	Azithromycin	0501050A0AA	1	0501050A0AAAAAA	TRUE
Clarithromyc	0501050B0AAAAAA	489006	Tab	Gran Straw		Clarithromyc	#N/A	1	0501050B0AAAMAM	TRUE
Clarithromyc	0501050B0AAAFAF	930	Pdr Sach	Gran Straw		Clarithromyc	#N/A	2	0501050B0AAAMAM	TRUE
Erythromycin	0501050C0AAABAB	3386647	Tab E/C	Cap	Y	Erythromycin	#N/A	2	0501050C0AAAFAF	TRUE
Erythromycin	0501050C0AAAKAK	83574	Cap E/C	Cap		Erythromycin	#N/A	2	0501050C0AAAFAF	TRUE
Erythromycin	0501050H0AAAAAA	536200	Ethylsuc Susp	Mix		Erythromycin	#N/A	2	0501050C0AAAIAI	TRUE
Erythromycin	0501050H0AAABAB	777400	Ethylsuc Susp	Mix		Erythromycin	#N/A	2	0501050C0AAAJAJ	TRUE
Erythromycin	0501050H0AAAEAE	33025	Ethylsuc Tab	Cap		Erythromycin	#N/A	2	0501050C0AAADAD	TRUE
Erythromycin	0501050H0AAAMAM	1236100	Ethylsuc Susp	Esuc Ctd Susp		Erythromycin	#N/A	2	0501050H0AAAPAP	TRUE
Clindamycin	0501060D0AAANAN	4742	Oral Susp	Oral Soln		Clindamycin	#N/A	2	0501060D0AAAEAE	TRUE
Fusidic Acid_	0501070M0AAAAAA	6300	Mix	Liq Spec		Fusidic Acid_	#N/A	1	0501070M0AAABAB	TRUE
Sod Fusidate	0501070N0AAADAD	21200	Tab	Cap		Sod Fusidate	#N/A	1	0501070N0AAAAAA	TRUE
Sulfapyridine	0501080V0AAADAD	448	Cap	Tab		Sulfapyridine	#N/A	1	0501080V0AAACAC	TRUE
Ethambutol H	0501090H0AAAZAZ	224	Liq Spec	Syr		Ethambutol H	#N/A	2	0501090H0AAANAN	TRUE
Ethambutol H	0501090H0AABCBC	250	Liq Spec	Syr		Ethambutol H	#N/A	2	0501090H0AAAJAJ	TRUE
Isoniazid_Tal	0501090K0AAAIAI	52572	Tab	Cap		Isoniazid_Ca	#N/A	1	0501090K0AACHCH	TRUE
Isoniazid_Ora	0501090K0AACUCU	5500	Oral Soln	Oral Susp		Isoniazid_Ora	#N/A	2	0501090K0AABIBI	TRUE
Pyrazinamide	0501090N0AAAAAA	1178	Tab	Cap		Pyrazinamide	#N/A	1	0501090N0AABYBY	TRUE
Pyrazinamide	0501090N0AABBBB	1200	Liq Spec	Susp		Pyrazinamide	#N/A	2	0501090N0AAAGAG	TRUE
Rifampicin_C	0501090R0AAAAAA	19785	Cap	Tab		Rifampicin_T	#N/A	1	0501090R0AAAHAH	TRUE
Rifampicin_C	0501090R0AAABAB	69026	Cap	Tab		Rifampicin_T	#N/A	1	0501090R0AAAIAI	TRUE
Rifampicin_C	0501090R0AAAFAF	46586	Oral Susp	Liq Spec		Rifampicin_L	#N/A	2	0501090R0AAALAL	TRUE
Dapsone_Tal	0501100H0AAAHAH	20104	Tab	Cap		Dapsone_Ca	#N/A	1	0501100H0AAAAAA	TRUE
Metronidazo	0501110C0AAAEAE	200558	Oral Susp	Liq Spec		Metronidazo	#N/A	2	0501110C0AABQBQ	TRUE
Metronidazo	0501110C0AAAGAG	863	Suppos	Tab	N	Metronidazo	0501110C0AA	1	0501110C0AABHBH	TRUE
Metronidazo	0501110C0AAAIAI	67716	Tab	Suppos		Metronidazo	#N/A	1	0501110C0AABJBJ	TRUE
Metronidazo	0501110C0AABHBH	10848	Tab	Suppos	N	Metronidazo	0501110C0AA	1	0501110C0AAAGAG	TRUE
Ciprofloxacin	0501120L0AAAFAF	716884	Tab	Pdrs		Ciprofloxacin	#N/A	1	0501120L0AABABA	TRUE
Ciprofloxacin	0501120L0AAAGAG	6639	Tab	Cap		Ciprofloxacin	#N/A	1	0501120L0AAAZAZ	TRUE
Ciprofloxacin	0501120L0AABGBG	198100	Gran For Susp	Liq Spec		Ciprofloxacin	#N/A	3	0501120L0AAASAS	TRUE
Nitrofurantoi	0501130R0AAAAAA	1815067	Cap	Pdrs		Nitrofurantoi	#N/A	1	0501130R0AACLCL	TRUE
Nitrofurantoi	0501130R0AAABAB	339689	Cap	Tab	Y	Nitrofurantoi	0501130R0AA	1	0501130R0AAAEAE	TRUE
Nitrofurantoi	0501130R0AAADAD	1404408	Tab	Cap	Y	Nitrofurantoi	0501130R0AA	1	0501130R0AAAAAA	TRUE
Nitrofurantoi	0501130R0AAAEAE	475730	Tab	Cap	Y	Nitrofurantoi	0501130R0AA	1	0501130R0AAABAB	TRUE
Amphoterici	0502030A0AAAAAA	28	Inf(Sod Desoxych	Inf (In Liposom	N	Amphoterici	#N/A	2	0502030A0AAAIAI	FALSE
Nystatin_Ora	0502030B0AAABAB	898320	Oral Susp	Ear Dps		Nystatin_Ear	#N/A	2	1201010K0AAAAAA	TRUE
Nystatin_Ora	0502030B0AAAXAX	541	Oral Susp	Gran For Susp		Nystatin_Gra	#N/A	2	0502030B0AAAFAF	TRUE
Griseofulvin_	0502050B0AACUCU	15068	Oral Susp	Liq Spec		Griseofulvin_	#N/A	2	0502050B0AAAFAF	TRUE
Terbinafine H	0502050C0AAAAAA	1796889	Tab	Suppos		Terbinafine H	#N/A	1	0502050C0AAACAC	TRUE
Terbinafine H	0502050C0AAAEAE	1445	Oral Soln	Oral Susp	Y	Terbinafine H	0502050C0AA	2	0502050C0AAAFAF	TRUE
Terbinafine H	0502050C0AAAFAF	3310	Oral Susp	Oral Soln	Y	Terbinafine H	0502050C0AA	2	0502050C0AAAEAE	TRUE
Ritonavir_Ta	0503010U0AAACAC	88	Tab	Cap		Ritonavir_Ca	#N/A	1	0503010U0AAAAAA	TRUE
Aciclovir_Tab	0503021C0AAABAB	735144	Tab	Tab Disper	N	Aciclovir_Tab	0503021C0AA	1	0503021C0AAAGAG	TRUE
Aciclovir_Tab	0503021C0AAACAC	1587395	Tab	Tab Disper	Y	Aciclovir_Tab	0503021C0AA	1	0503021C0AAAHAH	TRUE
Aciclovir_Tab	0503021C0AAADAD	668078	Tab	Tab Disper	N	Aciclovir_Tab	0503021C0AA	1	0503021C0AAAEAE	TRUE
Aciclovir_Tab	0503021C0AAAEAE	58953	Tab Disper	Tab	Y	Aciclovir_Tab	0503021C0AA	2	0503021C0AAADAD	TRUE
Aciclovir_Tab	0503021C0AAAGAG	152190	Tab Disper	Tab	N	Aciclovir_Tab	0503021C0AA	2	0503021C0AAABAB	TRUE
Aciclovir_Tab	0503021C0AAAHAH	115382	Tab Disper	Tab	N	Aciclovir_Tab	0503021C0AA	2	0503021C0AAACAC	TRUE
Ribavirin_Ca	0503050B0AAABAB	84	Cap	Tab		Ribavirin_Tal	#N/A	1	0503050B0AAAEAE	TRUE
Proguanil HC	0504010M0AAAAAA	1391	Tab	Pdrs		Proguanil HC	#N/A	1	0504010M0AAABAB	TRUE
Quinine Bisul	0504010T0AAAEAE	1430906	Tab	Cap	Y	Quinine Bisul	#N/A	1	0504010T0AAAAAA	TRUE
Quinine Sulf_	0504010Y0AAAFAF	3619414	Tab	Cap	Y	Quinine Sulf_	#N/A	1	0504010Y0AAAJAJ	TRUE
Quinine Sulf_	0504010Y0AAAHAH	3742364	Tab	Cap	Y	Quinine Sulf_	#N/A	1	0504010Y0AAAAAA	TRUE
Quinine Sulf_	0504010Y0AABCBC	2700	Oral Susp	Liq Spec		Quinine Sulf_	#N/A	2	0504010Y0AAAXAX	TRUE

Mepacrine H	0504040M0AAAAAA	7318	Tab	Cap		Mepacrine H	#N/A	1	0504040M0AAAEAE	TRUE
Albendazole_	0505030A0AAABAB	37	Tab Chble	Tab	N	Albendazole_	0505030A0AA	2	0505030A0AAADAD	TRUE
Albendazole_	0505030A0AAADAD	80	Tab	Tab Chble	N	Albendazole_	0505030A0AA	1	0505030A0AAABAB	TRUE
Ins Solb_Inj (	0601011N0AAAAAA	14	Inj (Bov)	Inj (Hum Emp)		Ins Solb_Inj (	#N/A	2	0601011N0AAABAB	TRUE
Ins Solb_Inj (	0601011N0AAACAC	5	Inj (Pore)	Inj (Bov)	Y	Ins Solb_Inj (	0601011N0AA	2	0601011N0AAAAAA	TRUE
Ins Solb_Inj (	0601011N0AAAPAP	25	Inj (Hum Prb)	Inj (Bov)		Ins Solb_Inj (	#N/A	3	0601011N0AAAYAY	TRUE
Ins Isop_Inj (	0601012S0AAASAS	15	Inj (Bov)	Inj (Pore)	N	Ins Isop_Inj (	0601012S0AA	2	0601012S0AAATAT	TRUE
Ins Isop_Inj (	0601012S0AAATAT	196	Inj (Pore)	Inj (Bov)	N	Ins Isop_Inj (	0601012S0AA	2	0601012S0AAASAS	TRUE
Gliclazide_O	0601021M0AAASAS	15790	Oral Susp	Liq Spec		Gliclazide_Li	#N/A	2	0601021M0AAAEAE	TRUE
Gliclazide_O	0601021M0AAAUAU	7100	Oral Susp	Liq Spec		Gliclazide_Li	#N/A	2	0601021M0AAADAD	TRUE
Metformin H	0601022B0AAABAB	109953332	Tab	Pdrs		Metformin H	#N/A	1	0601022B0AAAPAP	TRUE
Metformin H	0601022B0AAADAD	9679233	Tab	Cap	Y	Metformin H	#N/A	1	0601022B0AAAQAQ	TRUE
Metformin H	0601022B0AAAIAI	20730	Liq Spec	Susp		Metformin H	#N/A	2	0601022B0AAAEAE	TRUE
Metformin H	0601022B0AAAJAJ	1000	Liq Spec	Susp		Metformin H	#N/A	2	0601022B0AAAGAG	TRUE
Diazoxide_Ta	0601040E0AAAAAA	13449	Tab	Cap		Diazoxide_Ca	#N/A	1	0601040E0AAAFAF	TRUE
Diazoxide_O	0601040E0AAAMAM	392	Oral Soln	Oral Susp	Y	Diazoxide_O	0601040E0AA	2	0601040E0AABIBI	TRUE
Diazoxide_O	0601040E0AABHBH	2500	Oral Susp	Oral Soln		Diazoxide_O	#N/A	2	0601040E0AAAYAY	TRUE
Diazoxide_O	0601040E0AABIBI	3980	Oral Susp	Oral Soln	Y	Diazoxide_O	0601040E0AA	2	0601040E0AAAMAM	TRUE
Glucagon_Inj	0601040H0AAAEAE	4008	Inj (rys)	Inj	N	Glucagon_Inj	#N/A	2	0601040H0AAAAAA	TRUE
Liothyronine	0602010M0AAAAAA	288049	Tab	Cap	Y	Liothyronine	#N/A	1	0602010M0AAARAR	TRUE
Liothyronine	0602010M0AAADAD	1824	Tab	Cap	Y	Liothyronine	0602010M0AA	1	0602010M0AAAEAE	TRUE
Liothyronine	0602010M0AAAEAE	44606	Cap	Pdrs		Liothyronine	#N/A	1	0602010M0AAAGAG	TRUE
Liothyronine	0602010M0AAAUAU	242	Cap	Tab		Liothyronine	#N/A	1	0602010M0AAAQAQ	TRUE
Levothyrox S	0602010V0AAAFAF	2667	Cap	Pdrs		Levothyrox S	#N/A	1	0602010V0AABPBP	TRUE
Levothyrox S	0602010V0AAAGAG	2845	Cap	Pdr Sach		Levothyrox S	#N/A	1	0602010V0AAAWAW	TRUE
Levothyrox S	0602010V0AABWBW	32271340	Tab	Cap	Y	Levothyrox S	0602010V0AA	1	0602010V0AAAGAG	TRUE
Levothyrox S	0602010V0AABXBX	31584502	Tab	Cap	Y	Levothyrox S	0602010V0AA	1	0602010V0AAAFAF	TRUE
Levothyrox S	0602010V0AABZBZ	34839926	Tab	Cap	Y	Levothyrox S	0602010V0AA	1	0602010V0AACMCM	TRUE
Levothyrox S	0602010V0AACJCJ	30	Cap	Pdrs	N	Levothyrox S	0602010V0AA	1	0602010V0AADNDN	TRUE
Levothyrox S	0602010V0AACMCM	2282	Cap	Pdrs	N	Levothyrox S	0602010V0AA	1	0602010V0AACQCQ	TRUE
Levothyrox S	0602010V0AACQCQ	30	Pdrs	Cap	N	Levothyrox S	0602010V0AA	1	0602010V0AACMCM	TRUE
Levothyrox S	0602010V0AACWCW	3650	Liq Spec	Susp		Levothyrox S	#N/A	2	0602010V0AAAKAK	TRUE
Levothyrox S	0602010V0AACXCX	6510	Liq Spec	Susp		Levothyrox S	#N/A	2	0602010V0AAAQAQ	TRUE
Levothyrox S	0602010V0AACYCY	5850	Liq Spec	Susp		Levothyrox S	#N/A	2	0602010V0AAALAL	TRUE
Levothyrox S	0602010V0AACZCZ	2200	Liq Spec	Susp		Levothyrox S	#N/A	2	0602010V0AAA8A8	TRUE
Levothyrox S	0602010V0AADCDC	140	Liq Spec	Susp		Levothyrox S	#N/A	2	0602010V0AABABA	TRUE
Levothyrox S	0602010V0AADNDN	118	Pdrs	Cap	N	Levothyrox S	0602010V0AA	1	0602010V0AACJCJ	TRUE
Carbimazole_	0602020D0AAAAAA	1878473	Tab	Cap	Y	Carbimazole_	#N/A	1	0602020D0AAACAC	TRUE
Carbimazole_	0602020D0AAABAB	429142	Tab	Cap	Y	Carbimazole_	#N/A	1	0602020D0AAANAN	TRUE
Carbimazole_	0602020D0AAAWAW	6020	Oral Susp	Oral Soln		Carbimazole_	#N/A	2	0602020D0AAAGAG	TRUE
Fludrocort A	0603010I0AAA8A8	501	Liq Spec	Susp		Fludrocort A	#N/A	2	0603010I0AAAMAM	TRUE
Fludrocort A	0603010I0AAACAC	1293900	Tab	Cap	Y	Fludrocort A	#N/A	1	0603010I0AAAIAI	TRUE
Fludrocort A	0603010I0AABYBY	6830	Oral Susp	Liq Spec		Fludrocort A	#N/A	2	0603010I0AAAZAZ	TRUE
Fludrocort A	0603010I0AABZBZ	5410	Oral Susp	Liq Spec		Fludrocort A	#N/A	2	0603010I0AAA1A1	TRUE
Cortisone Ac	0603020F0AAAHAH	1482	Tab	Cap		Cortisone Ac	#N/A	1	0603020F0AAARAR	TRUE
Dexameth_Li	0603020G0AAA6A6	780	Liq Spec	Mix		Dexameth_M	#N/A	2	0603020G0AAASAS	TRUE
Dexameth_Li	0603020G0AAA7A7	360	Liq Spec	Oral Soln		Dexameth_C	#N/A	2	0603020G0AAAWAW	TRUE
Dexameth_T	0603020G0AAABAB	189462	Tab	Pdrs		Dexameth_P	#N/A	1	0603020G0AAAZAZ	TRUE
Hydrocort_T	0603020J0AAADAD	2076212	Tab	Cap	Y	Hydrocort_C	#N/A	1	0603020J0AACHCH	TRUE
Hydrocort_Li	0603020J0AAAJAJ	1300	Liq Spec	Oral Susp	Y	Hydrocort_O	0603020J0AA	2	0603020J0AAAXAX	TRUE
Hydrocort_O	0603020J0AAAKAK	13430	Oral Susp	Liq Spec		Hydrocort_Li	#N/A	2	0603020J0AAAFAF	TRUE
Hydrocort_O	0603020J0AAAXAX	74384	Oral Susp	Liq Spec	Y	Hydrocort_Li	0603020J0AA	2	0603020J0AAAJAJ	TRUE
Hydrocort_Li	0603020J0AABLBL	400	Liq Spec	Oral Susp		Hydrocort_O	#N/A	2	0603020J0AAA4A4	TRUE
Prednisolone	0603020T0AAAAAA	7752541	Tab	Cap	Y	Prednisolone	#N/A	1	0603020T0AAAVAV	TRUE
Prednisolone	0603020T0AAABAB	203103	Tab	Suppos		Prednisolone	#N/A	1	0105020F0AAAFAF	TRUE
Prednisolone	0603020T0AAACAC	17168871	Tab	Suppos		Prednisolone	#N/A	1	0105020F0AAACAC	TRUE
Prednisolone	0603020T0AAAFAF	1174244	Tab E/C	Suppos		Prednisolone	#N/A	2	0105020F0AAAFAF	TRUE
Prednisolone	0603020T0AAAGAG	2262993	Tab E/C	Suppos		Prednisolone	#N/A	2	0105020F0AAACAC	TRUE
Prednisolone	0603020T0AAATAT	7860	Tab E/C	Cap		Prednisolone	#N/A	2	0603020T0AAAVAV	TRUE
Prednisolone	0603020T0AAAYAY	700	Liq Spec	Susp		Prednisolone	#N/A	2	0603020T0AAAMAM	TRUE
Prednisolone	0603020T0AAAZAZ	50	Liq Spec	Susp		Prednisolone	#N/A	2	0603020T0AAALAL	TRUE
Prednisolone	0603020T0AABHBH	591341	Tab Solb	Suppos		Prednisolone	#N/A	2	0105020F0AAACAC	TRUE
Prednisolone	0603020T0AABIBI	150	Liq Spec	Susp		Prednisolone	#N/A	2	0603020T0AAASAS	TRUE
Ethinylestr_T	0604011D0AAALAL	7302	Tab	Cap		Ethinylestr_C	#N/A	1	0604011D0AAAWAW	TRUE
Estradiol_Tab	0604011G0AAAIAI	341156	Tab	Pess		Estradiol_Pes	#N/A	1	0702010G0AAADAD	TRUE
Estradiol_Tab	0604011G0AABDBD	450796	Tab	Val Tab	Y	Estradiol_Val	0604011K0AA	1	0604011K0AAAAAA	TRUE
Estradiol_Val	0604011K0AAAAAA	195014	Val Tab	Tab	Y	Estradiol_Tab	0604011G0AA	2	0604011G0AABDBD	TRUE
Estradiol_Val	0604011K0AAABAB	203571	Val Tab	Pess		Estradiol_Pes	#N/A	2	0702010G0AAADAD	TRUE
Progesterone	0604012S0AAAEAE	8837	Pess	Cap		Progesterone	#N/A	1	0604012S0AAAUAU	TRUE
Progesterone	0604012S0AAANAN	483	Pess	Implant		Progesterone	#N/A	1	0604012S0AAAAAA	TRUE
Progesterone	0604012S0AABZBZ	1461	Vag Cap	Cap		Progesterone	#N/A	2	0604012S0AABWBW	TRUE
Finasteride_	0604020C0AAAAAA	8227991	Tab	Pdr Sach		Finasteride_I	#N/A	1	0604020C0AAADAD	TRUE
Testosterone	0604020K0AABHBH	304031	Gel Sach	Gel	Y	Testosterone	0604020K0AA	2	0604020K0AABKBK	TRUE
Testosterone	0604020K0AABKBK	20253	Gel	Gel Sach	N	Testosterone	0604020K0AA	1	0604020K0AABHBH	TRUE
Testosterone	0604020K0AABMBM	225000	Gel	Crm		Testosterone	#N/A	1	0604020K0AAAGAG	TRUE
Testosterone	0604020P0AAAKAK	50	Crm	Oint		Testosterone	#N/A	1	0604020P0AAAJAJ	TRUE
Prasterone_(	0604030Q0AAAAAA	290	Cap	Tab		Prasterone_T	#N/A	1	0604030Q0AAAIAI	TRUE
Desmopressi	0605020E0AAALAL	112	Cap	Pdrs		Desmopressi	#N/A	1	0605020E0AAATAT	TRUE
Estradiol_Pe	0702010G0AAAGAG	757836	Pess	Val Tab		Estradiol_Val	#N/A	1	0604011K0AAACAC	TRUE
Clotrimazole	0702020F0AAACAC	20	Vag Crm	Crm	Y	Clotrimazole	0702020F0AA	2	0702020F0AAAJAJ	TRUE
Clotrimazole	0702020F0AAAJAJ	549620	Crm	Vag Crm	Y	Clotrimazole	0702020F0AA	1	0702020F0AAACAC	TRUE
Econazole Ni	0702020H0AAAAAA	7035	Crm	Lot		Econazole Ni	#N/A	1	1310020J0AAABAB	TRUE
Econazole Ni	0702020H0AAAEAE	125	Pess L/A	Pess	Y	Econazole Ni	#N/A	2	0702020H0AAABAB	TRUE
Fenticonazol	0702020I0AAADAD	9	Vag Cap	Pess		Fenticonazol	#N/A	2	0702020I0AAABAB	TRUE

Fenticonazol	0702020I0AAAEAE	114	Vag Cap	Pess		Fenticonazol	#N/A	2	0702020I0AAAAAA	TRUE
Nystatin_Pes	0702020T0AAAFAF	71	Pess	Pess Eff		Nystatin_Pes	#N/A	1	0702020T0AAAAAA	TRUE
Metronidazo	0702020X0AAAAAA	123520	Vag Gel	Crm	Y	Metronidazo	1310012K0AA	2	1310012K0AAAXAX	TRUE
Boric Acid_P	0702020Y0AAAAAA	60	Pess	Suppos		Boric Acid_Su	#N/A	1	0107010H0AAAAAA	TRUE
Nonoxinol 9_	0703030G0AAAIAI	9474	Gel	Crm		Nonoxinol 9_	#N/A	1	0703030G0AAABAB	TRUE
Tamsulosin H	0704010U0AAAAAA	16530270	Cap	Tab	Y	Tamsulosin H	0704010U0AA	1	0704010U0AAABAB	TRUE
Tamsulosin H	0704010U0AAABAB	527693	Tab	Cap	Y	Tamsulosin H	0704010U0AA	1	0704010U0AAAAAA	TRUE
Solifenacin_T	0704020ABAAAAAA	3737219	Tab	Pdr Sach		Solifenacin_P	#N/A	1	0704020ABAAACAC	TRUE
Oxybutynin H	0704020J0AAACAC	2487099	Tab	Suppos		Oxybutynin H	#N/A	1	0704020J0AAAQAQ	TRUE
Oxybutynin H	0704020J0AAAIAI	44236	Oral Soln	Liq Spec	Y	Oxybutynin H	0704020J0AA	2	0704020J0AAAKAK	TRUE
Oxybutynin H	0704020J0AAAKAK	74960	Liq Spec	Oral Soln	Y	Oxybutynin H	0704020J0AA	2	0704020J0AAAIAI	TRUE
Oxybutynin H	0704020J0AAAMAM	2500	Liq Spec	Oral Soln		Oxybutynin H	#N/A	2	0704020J0AAAWAW	TRUE
Tolterodine_	0704020N0AAABAB	2123895	Tab	Pdr Sach		Tolterodine_	#N/A	1	0704020N0AAAFAF	TRUE
Tolterodine_	0704020N0AAAJAJ	11680	Oral Susp	Oral Soln		Tolterodine_	#N/A	2	0704020N0AAAEAE	TRUE
Pot Cit_Cap	0704030G0AAAPAP	1460	Cap	Pdrs		Pot Cit_Pdrs	#N/A	1	0704030G0AAAUAU	TRUE
Sod Cit_Pdr S	0704030J0AAAHAH	774	Pdr Sach	Gran Sach	Y	Sod Cit_Gran	0704030J0AA	2	0704030J0AAAIAI	TRUE
Sod Cit_Gran	0704030J0AAAIAI	676	Gran Sach	Pdr Sach	Y	Sod Cit_Pdr S	0704030J0AA	2	0704030J0AAAHAH	TRUE
Alprostadil_C	0704050B0AAAVAV	454	Cont Pack Inj	S/Pack Inj	Y	Alprostadil_S	0704050B0AA	3	0704050B0AABLBL	TRUE
Alprostadil_C	0704050B0AAAWAW	227	Cont Pack Inj	S/Pack Inj	N	Alprostadil_S	0704050B0AA	3	0704050B0AABMBM	TRUE
Alprostadil_U	0704050B0AAAZAZ	609	Urethral Stick	Urethral Suppos		Alprostadil_U	#N/A	2	0704050B0AAASAS	TRUE
Alprostadil_C	0704050B0AABFBF	514	Cont Pack Inj	S/Pack Inj	N	Alprostadil_S	0704050B0AA	3	0704050B0AABNBN	TRUE
Alprostadil_S	0704050B0AABLBL	12	S/Pack Inj	Cont Pack Inj	Y	Alprostadil_C	0704050B0AA	2	0704050B0AAAVAV	TRUE
Alprostadil_S	0704050B0AABMBM	13	S/Pack Inj	Cont Pack Inj	Y	Alprostadil_C	0704050B0AA	2	0704050B0AAAWAW	TRUE
Alprostadil_S	0704050B0AABNBN	7	S/Pack Inj	Cont Pack Inj	N	Alprostadil_C	0704050B0AA	2	0704050B0AABFBF	TRUE
Yohimbine H	0704050Y0AAAMAM	16	Tab	Cap		Yohimbine H	#N/A	1	0704050Y0AAACAC	TRUE
Sildenafil_Ta	0704050Z0AAABAB	111337	Tab	Pess		Sildenafil_Pe	#N/A	1	0604012V0AAAAAA	TRUE
Sildenafil_Or	0704050Z0AAAFAF	1100	Oral Soln	Oral Susp	Y	Sildenafil_Or	0704050Z0AA	2	0704050Z0AAALAL	TRUE
Sildenafil_Or	0704050Z0AAAGAG	537	Oral Soln	Oral Susp	Y	Sildenafil_Or	0704050Z0AA	2	0704050Z0AAAKAK	TRUE
Sildenafil_Or	0704050Z0AAAKAK	7532	Oral Susp	Oral Soln	Y	Sildenafil_Or	0704050Z0AA	2	0704050Z0AAAGAG	TRUE
Sildenafil_Or	0704050Z0AAALAL	2150	Oral Susp	Oral Soln	Y	Sildenafil_Or	0704050Z0AA	2	0704050Z0AAAFAF	TRUE
Calc Folinate	0801000I0AAAHAH	8986	Tab	Cap		Calc Folinate	#N/A	1	0801000I0AAAUAU	TRUE
Calc Folinate	0801000I0AAAWAW	300	Liq Spec	Mthwsh		Calc Folinate	#N/A	2	0801000I0AAAVAV	TRUE
Mercaptopur	0801030L0AAABAB	3346	Tab	Cap	Y	Mercaptopur	0801030L0AA	1	0801030L0AAAGAG	TRUE
Mercaptopur	0801030L0AAAGAG	3526	Cap	Tab	Y	Mercaptopur	0801030L0AA	1	0801030L0AAABAB	TRUE
Mercaptopur	0801030L0AAALAL	28	Cap	Tab		Mercaptopur	#N/A	1	0801030L0AAAJAJ	TRUE
Imatinib Mes	0801050AAAAACAC	156	Tab	Cap		Imatinib Mes	#N/A	1	0801050AAAAAAAA	TRUE
Hydroxycarb	0801050P0AAABAB	4900	Oral Soln	Oral Susp	Y	Hydroxycarb	0801050P0AA	2	0801050P0AAADAD	TRUE
Hydroxycarb	0801050P0AAADAD	3490	Oral Susp	Oral Soln	Y	Hydroxycarb	0801050P0AA	2	0801050P0AAABAB	TRUE
Azathioprine	0802010G0AAADAD	700719	Tab	Cap	Y	Azathioprine	#N/A	1	0802010G0AABWBW	TRUE
Azathioprine	0802010G0AAAEAE	4127793	Tab	Cap	Y	Azathioprine	#N/A	1	0802010G0AABUBU	TRUE
Azathioprine	0802010G0AAAHAH	638	Cap	Pdrs		Azathioprine	#N/A	1	0802010G0AABMBM	TRUE
Azathioprine	0802010G0AAAPAP	9660	Oral Soln	Oral Susp	Y	Azathioprine	0802010G0AA	2	0802010G0AACHCH	TRUE
Azathioprine	0802010G0AACHCH	34332	Oral Susp	Oral Soln	Y	Azathioprine	0802010G0AA	2	0802010G0AAAPAP	TRUE
Azathioprine	0802010G0AACICI	5150	Oral Susp	Oral Soln		Azathioprine	#N/A	2	0802010G0AAASAS	TRUE
Tacrolimus_C	0802020T0AAAGAG	4768	Oral Soln	Oral Susp		Tacrolimus_C	#N/A	2	0802020T0AAAZAZ	TRUE
Tacrolimus_L	0802020T0AAALAL	8480	Liq Spec	Oral Susp		Tacrolimus_C	#N/A	2	0802020T0AAAYAY	TRUE
Tacrolimus_C	0802020T0AAANAN	3000	Cap	Tab		Tacrolimus_T	#N/A	1	0802020T0AABCBC	TRUE
Tacrolimus_C	0802020T0AABFBF	200	Cap	Pdrs		Tacrolimus_P	#N/A	1	0802020T0AAADAD	TRUE
Diethylstilbe	0803010K0AAAKAK	38939	Tab	Cap		Diethylstilbe	#N/A	1	0803010K0AAAMAM	TRUE
Anastrozole_	0803041B0AAAAAA	1597392	Tab	Cap	Y	Anastrozole_	#N/A	1	0803041B0AAACAC	TRUE
Tamoxifen Ci	0803041S0AAAHAH	900	Oral Susp	Susp		Tamoxifen Ci	#N/A	2	0803041S0AAAEAE	TRUE
Ferr Fumar_C	0901011F0AAACAC	2051240	Oral Soln	Liq Spec		Ferr Fumar_L	#N/A	2	0901011F0AAAJAJ	TRUE
Ferr Fumar_C	0901011F0AAAHAH	2079334	Cap	Tab		Ferr Fumar_T	#N/A	1	0901011F0AAADAD	TRUE
Ferr Sulf_Tab	0901011P0AAACAC	13930400	Tab	Cap	Y	Ferr Sulf_Cap	#N/A	1	0901011P0AAAUAU	TRUE
Ferr Sulf_Ora	0901011P0AACKCK	1900	Oral Soln	Liq Spec		Ferr Sulf_Liq	#N/A	2	0901011P0AABPBP	TRUE
Ferr Sulf_Ora	0901011P0AACLCL	1400	Oral Susp	Liq Spec		Ferr Sulf_Liq	#N/A	2	0901011P0AABPBP	TRUE
Folic Acid_Ta	0901020G0AAAGAG	13230128	Tab	Cap	Y	Folic Acid_Ca	#N/A	1	0901020G0AABTBT	TRUE
Folic Acid_Ta	0901020G0AABFBF	1085622	Tab	Cap	Y	Folic Acid_Ca	#N/A	1	0901020G0AABNBN	TRUE
Folic Acid_Li	0901020G0AABZBZ	100	Liq Spec	Susp		Folic Acid_Su	#N/A	2	0901020G0AAAVAV	TRUE
Folic Acid_Or	0901020G0AACCCC	5810	Oral Soln	Oral Susp		Folic Acid_Or	#N/A	2	0901020G0AACZCZ	TRUE
St.Marks_Ora	0902012H0AAAKAK	477	Oral Rehydration	Electrolyte Pdrs		St.Marks_Ele	#N/A	3	0902012H0AAAIAI	TRUE
Sod Chlor_Ca	0902012L0AAAAAA	772	Cap	Tab		Sod Chlor_Ta	#N/A	1	0902012L0AAALAL	TRUE
Sod Chlor_Ca	0902012L0AAARAR	928	Cap	Tab		Sod Chlor_Ta	#N/A	1	0902012L0AAAIAI	TRUE
Sod Chlor_Ca	0902012L0AAAUAU	100	Cap	Tab		Sod Chlor_Ta	#N/A	1	0902012L0AAAMAM	TRUE
Sod Chlor_Li	0902012L0AABRBR	25350	Liq Spec	Oral Soln		Sod Chlor_Or	#N/A	2	0902012L0AADDDD	TRUE
Sod Chlor_Or	0902012L0AADFDF	30660	Oral Soln	Liq Spec		Sod Chlor_Li	#N/A	2	0902012L0AACACA	TRUE
Pot Bicarb_C	0902013P0AAABAB	1368	Cap	Tab		Pot Bicarb_T	#N/A	1	0902013P0AAADAD	TRUE
Sod Bicarb_C	0902013S0AAACAC	3586981	Cap	Pdrs		Sod Bicarb_P	#N/A	1	0101012B0AAAKAK	TRUE
Sod Bicarb_C	0902013S0AAADAD	112	Cap	Tab	Y	Sod Bicarb_T	0902013S0AA	1	0902013S0AAAPAP	TRUE
Sod Bicarb_C	0902013S0AAAFAF	60	Cap	Tab		Sod Bicarb_T	#N/A	1	0902013S0AAAQAQ	TRUE
Sod Bicarb_T	0902013S0AAAPAP	77467	Tab	Cap	Y	Sod Bicarb_C	0902013S0AA	1	0902013S0AAADAD	TRUE
Sod Chlor_I/V	0902021S0AAA2A2	57	I/V Inf	Ster Buff Spy		Sod Chlor_St	#N/A	2	1311010S0AAAVAV	TRUE
Sod Chlor_I/V	0902021S0AAAXAX	666572	I/V Inf	Eye Dps	N	Sod Chlor_Ey	1108010K0AA	2	1108010K0AAAAAA	TRUE
Sod Chlor_I/V	0902021S0AAAYAY	157	I/V Inf	Blad Irrig		Sod Chlor_Bl	#N/A	2	0704040J0AAAGAG	TRUE
Sod Chlor_I/V	0902021S0AAAZAZ	339	I/V Inf	Blad Irrig		Sod Chlor_Bl	#N/A	2	0704040J0AAAMAM	TRUE
Sod Chlor_I/V	0902021S0AACJCJ	258	I/V Inf	Blad Irrig		Sod Chlor_Bl	#N/A	2	0704040J0AAAFAF	TRUE
Sod Chlor_I/V	0902021S0AACQCQ	2	I/V Inf	Blad Irrig		Sod Chlor_Bl	#N/A	2	0704040J0AAARAR	TRUE
Calc Carb_Ta	0905011D0AAADAD	53219	Tab Eff	Cap		Calc Carb_Ca	#N/A	2	0101021C0AAAHAH	TRUE
Calc Carb_Ta	0905011D0AAAEAE	7129	Tab	Cap		Calc Carb_Ca	#N/A	1	0101021C0AAAHAH	TRUE
Calc Glucon_	0905011K0AAAAAA	7000	Tab Eff	Tab	N	Calc Glucon_	#N/A	2	0905011K0AAAHAH	TRUE
Calc Glucon_	0905011K0AAAGAG	360	Tab	Cap		Calc Glucon_	#N/A	1	0905011K0AAARAR	TRUE
Mag Glycero	0905013G0AAA2A2	24554	Tab	Cap	Y	Mag Glycero	0905013G0AA	1	0905013G0AAA4A4	TRUE
Mag Glycero	0905013G0AAA4A4	16110	Cap	Pdrs		Mag Glycero	#N/A	1	0905013G0AABVBV	TRUE
Mag Glycero	0905013G0AABMBM	7632	Cap	Tab	Y	Mag Glycero	0905013G0AA	1	0905013G0AACXCX	TRUE

Mag Glycero	0905013G0AACVCV	84275	Oral Soln	Oral Susp	Y	Mag Glycero	0905013G0AA	2	0905013G0AACWCW	TRUE
Mag Glycero	0905013G0AACWCW	11850	Oral Susp	Oral Soln	Y	Mag Glycero	0905013G0AA	2	0905013G0AACVCV	TRUE
Mag Glycero	0905013G0AACXCX	336	Tab	Cap	Y	Mag Glycero	0905013G0AA	1	0905013G0AABMBM	TRUE
Mag Glycero	0905013G0AACZCZ	1000	Oral Susp	Oral Soln		Mag Glycero	#N/A	2	0905013G0AABXBX	TRUE
Mag Orotate	0905013M0AAACAC	1008	Tab	Cap		Mag Orotate	#N/A	1	0905013M0AAADAD	TRUE
Phos/Sod_O	090502100AAAMAM	16740	Oral Soln	Oral Susp		Phos/Sod_O	#N/A	2	090502100AAANAN	TRUE
Sod Dihydrog	0905021L0AAAGAG	600	Oral Susp	Oral Soln	Y	Sod Dihydrog	0905021L0AA	2	0905021L0AAASAS	TRUE
Sod Dihydrog	0905021L0AAASAS	10460	Oral Soln	Oral Susp	Y	Sod Dihydrog	0905021L0AA	2	0905021L0AAAGAG	TRUE
Sod Fluoride_	0905030G0AAATAT	608	Tab	Cap		Sod Fluoride_	#N/A	1	0905030G0AAAHAH	TRUE
Zn Sulf_Cap 2	0905041Q0AAAAAA	43971	Cap	Tab		Zn Sulf_Tab 2	#N/A	1	0905041Q0AAAMAM	TRUE
Selenium_Or	0905050A0AAAAAA	3123	Oral Soln	Inj	N	Selenium_Inj	0905050A0AA	2	0905050A0AAACAC	TRUE
Selenium_Inj	0905050A0AAACAC	466	Inj	Oral Soln	N	Selenium_Or	0905050A0AA	1	0905050A0AAAAAA	TRUE
Betacarotene	0906012B0AAACAC	168	Cap	Tab		Betacarotene	#N/A	1	0906012B0AAALAL	TRUE
Nicotinamide	0906022K0AAAAAA	1570	Tab	Cap		Nicotinamide	#N/A	1	0906022K0AAAHAH	TRUE
Nicotinamide	0906022K0AAACAC	1830	Tab	Cap	Y	Nicotinamide	0906022K0AA	1	0906022K0AAAGAG	TRUE
Nicotinamide	0906022K0AAAGAG	2514	Cap	Tab	Y	Nicotinamide	0906022K0AA	1	0906022K0AAACAC	TRUE
Nicotinamide	0906022K0AAAPAP	3272	Tab	Cap		Nicotinamide	#N/A	1	0906022K0AAAMAM	TRUE
Pyridox HCl_	0906024N0AAAGAG	79135	Tab	Cap	Y	Pyridox HCl_	#N/A	1	0906024N0AABJBJ	TRUE
Pyridox HCl_	0906024N0AAAIAI	336845	Tab	Cap	Y	Pyridox HCl_	#N/A	1	0906024N0AAATAT	TRUE
Pyridox HCl_	0906024N0AAAJAJ	240	Tab	Cap		Pyridox HCl_	#N/A	1	0906024N0AABEBE	TRUE
Pyridox HCl_	0906024N0AAANAN	4131	Tab	Cap		Pyridox HCl_	#N/A	1	0906024N0AAAQAQ	TRUE
Pyridox HCl_	0906024N0AABMBM	600	Liq Spec	Oral Soln		Pyridox HCl_	#N/A	2	0906024N0AABABA	TRUE
Pyridox HCl_	0906024N0AABUBU	500	Liq Spec	Susp		Pyridox HCl_	#N/A	2	0906024N0AABCBC	TRUE
Pyridox HCl_	0906024N0AABWBW	1200	Liq Spec	Susp		Pyridox HCl_	#N/A	2	0906024N0AAA9A9	TRUE
Pyridox HCl_	0906024N0AACJCJ	420	Liq Spec	Susp		Pyridox HCl_	#N/A	2	0906024N0AABBBB	TRUE
Pyridox HCl_	0906024N0AACXCX	3550	Oral Soln	Liq Spec		Pyridox HCl_	#N/A	2	0906024N0AABLBL	TRUE
Pyridox HCl_	0906024N0AACYCY	4800	Oral Susp	Liq Spec		Pyridox HCl_	#N/A	2	0906024N0AABLBL	TRUE
Riboflavin_Li	0906025P0AAA3A3	150	Liq Spec	Syr		Riboflavin_Sy	#N/A	2	0906025P0AAAJAJ	TRUE
Riboflavin_Li	0906025P0AAA9A9	560	Liq Spec	Syr		Riboflavin_Sy	#N/A	2	0906025P0AAAVAV	TRUE
Riboflavin_Ta	0906025P0AAAAAA	456	Tab	Cap	Y	Riboflavin_Ca	0906025P0AA	1	0906025P0AABFBF	TRUE
Riboflavin_Ta	0906025P0AAAQAQ	28	Tab	Cap		Riboflavin_Ca	#N/A	1	0906025P0AAACAC	TRUE
Riboflavin_Ca	0906025P0AAAUAU	11210	Cap	Pdrs		Riboflavin_Pd	#N/A	1	0906025P0AAAKAK	TRUE
Riboflavin_Ca	0906025P0AABFBF	6727	Cap	Pdrs		Riboflavin_Pd	#N/A	1	0906025P0AABEBE	TRUE
Riboflavin_Ta	0906025P0AABIBI	30	Tab	Cap	Y	Riboflavin_Ca	0906025P0AA	1	0906025P0AAAUAU	TRUE
Thiamine HC	0906026M0AAAGAG	7643861	Tab	Cap	Y	Thiamine HC	#N/A	1	0906026M0AABEBE	TRUE
Thiamine HC	0906026M0AAAXAX	1910	Oral Soln	Oral Susp	Y	Thiamine HC	0906026M0AA	2	0906026M0AABKBK	TRUE
Thiamine HC	0906026M0AABIBI	5880	Oral Soln	Liq Spec		Thiamine HC	#N/A	2	0906026M0AAA1A1	TRUE
Thiamine HC	0906026M0AABJBJ	2060	Oral Susp	Liq Spec		Thiamine HC	#N/A	2	0906026M0AAA1A1	TRUE
Thiamine HC	0906026M0AABKBK	1700	Oral Susp	Oral Soln	Y	Thiamine HC	0906026M0AA	2	0906026M0AAAXAX	TRUE
Biotin_Tab 5	090602800AAAGAG	10750	Tab	Pdrs		Biotin_Pdrs 5	#N/A	1	090602800AACECE	TRUE
Pot Aminobe	090602800AAANAN	2352	Cap	Tab		Pot Aminobe	#N/A	1	090602800AAAVAV	TRUE
Biotin_Tab 1	090602800AACPCP	1200	Tab	Cap		Biotin_Cap 1	#N/A	1	090602800AACQCQ	TRUE
Ascorbic Aci	0906031C0AAAFAF	115511	Tab	Cap	Y	Ascorbic Aci	#N/A	1	0906031C0AAA9A9	TRUE
Ascorbic Aci	0906031C0AAAGAG	118448	Tab	Pdrs		Ascorbic Aci	#N/A	1	0906031C0AABTBT	TRUE
Ascorbic Aci	0906031C0AAAHAH	79444	Tab	Tab Chble	N	Ascorbic Aci	#N/A	1	0906031C0AABMBM	TRUE
Ascorbic Aci	0906031C0AAAIAI	188142	Tab	Cap	Y	Ascorbic Aci	#N/A	1	0906031C0AABIBI	TRUE
Ascorbic Aci	0906031C0AAALAL	597	Tab Chble	Cap		Ascorbic Aci	#N/A	2	0906031C0AABIBI	TRUE
Ascorbic Aci	0906031C0AAAPAP	14	Tab	Tab Chble		Ascorbic Aci	#N/A	1	0906031C0AAAKAK	TRUE
Ascorbic Aci	0906031C0AAAUAU	14	Tab Eff	Cap		Ascorbic Aci	#N/A	2	0906031C0AABIBI	TRUE
Ascorbic Aci	0906031C0AABABA	84	Cap	Tab	Y	Ascorbic Aci	0906031C0AA	1	0906031C0AABJBJ	TRUE
Ascorbic Aci	0906031C0AABJBJ	388	Tab	Cap	Y	Ascorbic Aci	0906031C0AA	1	0906031C0AABABA	TRUE
Ascorbic Aci	0906031C0AABNBN	1390	Tab Chble	Pdrs		Ascorbic Aci	#N/A	2	0906031C0AABTBT	TRUE
Colecal_Tab	0906040G0AAACAC	17198	Tab	Cap	Y	Colecal_Cap	0906040G0AA	1	0906040G0AAAHAH	TRUE
Colecal_Cap	0906040G0AAAHAH	645	Cap	Tab	Y	Colecal_Tab	0906040G0AA	1	0906040G0AAACAC	TRUE
Colecal_Cap	0906040G0AAANAN	3127999	Cap	Tab	Y	Colecal_Tab	0906040G0AA	1	0906040G0AACSCS	TRUE
Colecal_Oral	0906040G0AAATAT	200	Oral Susp	Oral Soln	Y	Colecal_Oral	0906040G0AA	2	0906040G0AACUCU	TRUE
Colecal_Oral	0906040G0AAAUAU	190	Oral Susp	Oral Soln		Colecal_Oral	#N/A	2	0906040G0AACMCM	TRUE
Colecal_Cap	0906040G0AABABA	188	Cap	Tab	Y	Colecal_Tab	0906040G0AA	1	0906040G0AADBDB	TRUE
Colecal_Cap	0906040G0AABBBB	2570	Cap	Tab	Y	Colecal_Tab	0906040G0AA	1	0906040G0AACYCY	TRUE
Colecal_Cap	0906040G0AABCBC	3609	Cap	Tab	Y	Colecal_Tab	0906040G0AA	1	0906040G0AACQCQ	TRUE
Colecal_Cap	0906040G0AABDBD	36	Cap	Tab	Y	Colecal_Tab	0906040G0AA	1	0906040G0AACRCR	TRUE
Colecal_Cap	0906040G0AABEBE	5328	Cap	Tab	Y	Colecal_Tab	0906040G0AA	1	0906040G0AACPCP	TRUE
Colecal_Tab	0906040G0AABGBG	402861	Tab	Cap	Y	Colecal_Cap	0906040G0AA	1	0906040G0AABHBH	TRUE
Colecal_Cap	0906040G0AABHBH	497926	Cap	Tab	Y	Colecal_Tab	0906040G0AA	1	0906040G0AABGBG	TRUE
Colecal_Cap	0906040G0AABIBI	255240	Cap	Tab	Y	Colecal_Tab	0906040G0AA	1	0906040G0AABRBR	TRUE
Colecal_Cap	0906040G0AABKBK	3496	Cap	Tab	Y	Colecal_Tab	0906040G0AA	1	0906040G0AACNCN	TRUE
Colecal_Oral	0906040G0AABNBN	4502	Oral Soln	Oral Susp		Colecal_Oral	#N/A	2	0906040G0AAAXAX	TRUE
Colecal_Tab	0906040G0AABRBR	172925	Tab	Cap	Y	Colecal_Cap	0906040G0AA	1	0906040G0AABIBI	TRUE
Colecal & Cal	0906040G0AABSBS	149125	Tab	Tab Chble	Y	Colecal & Cal	0906040G0AA	1	0906040G0AABYBY	TRUE
Colecal_Oral	0906040G0AABTBT	27063	Oral Dps	Oral Soln		Colecal_Oral	#N/A	2	0906040G0AACKCK	TRUE
Colecal & Cal	0906040G0AABWBW	4952150	Tab Chble	Tab	Y	Colecal & Cal	0906040G0AA	2	0906040G0AACCCC	TRUE
Colecal & Cal	0906040G0AABYBY	8076605	Tab Chble	Tab	Y	Colecal & Cal	0906040G0AA	2	0906040G0AABSBS	TRUE
Colecal & Cal	0906040G0AACACA	196	Tab Chble	Tab Eff	Y	Colecal & Cal	0906040G0AA	2	0906040G0AACBCB	TRUE
Colecal & Cal	0906040G0AACBCB	736568	Tab Eff	Tab Chble	Y	Colecal & Cal	0906040G0AA	2	0906040G0AACACA	TRUE
Colecal & Cal	0906040G0AACCCC	229304	Tab	Tab Chble	Y	Colecal & Cal	0906040G0AA	1	0906040G0AABWBW	TRUE
Colecal & Cal	0906040G0AACECE	120	Tab Chble	Tab	Y	Colecal & Cal	0906040G0AA	2	0906040G0AABSBS	TRUE
Colecal_Oral	0906040G0AACLCL	18	Oral Dps	Oral Soln	N	Colecal_Oral	0906040G0AA	2	0906040G0AADGDG	TRUE
Colecal_Tab	0906040G0AACNCN	10817	Tab	Cap	Y	Colecal_Cap	0906040G0AA	1	0906040G0AABKBK	TRUE
Colecal_Tab	0906040G0AACPCP	9072	Tab	Cap	Y	Colecal_Cap	0906040G0AA	1	0906040G0AABEBE	TRUE
Colecal_Tab	0906040G0AACQCQ	18350	Tab	Cap	Y	Colecal_Cap	0906040G0AA	1	0906040G0AABCBC	TRUE
Colecal_Tab	0906040G0AACRCR	31824	Tab	Cap	Y	Colecal_Cap	0906040G0AA	1	0906040G0AABDBD	TRUE
Colecal_Tab	0906040G0AACSCS	651500	Tab	Cap	Y	Colecal_Cap	0906040G0AA	1	0906040G0AAANAN	TRUE
Colecal_Oral	0906040G0AACUCU	11100	Oral Soln	Oral Susp	Y	Colecal_Oral	0906040G0AA	2	0906040G0AAATAT	TRUE
Colecal & Cal	0906040G0AACWCW	6329	Tab	Tab Chble	Y	Colecal & Cal	0906040N0AA	1	0906040N0AAEXEX	TRUE

Colecal_Tab	0906040G0AACYCY	1620	Tab	Cap	Y	Colecal_Cap	0906040G0AA	1	0906040G0AABBBB	TRUE
Colecal_Tab	0906040G0AADBDB	31962	Tab	Cap	Y	Colecal_Cap	0906040G0AA	1	0906040G0AABABA	TRUE
Colecal_Oral	0906040G0AADGDG	210	Oral Soln	Oral Dps	N	Colecal_Oral	0906040G0AA	2	0906040G0AACLCL	TRUE
Colecal_Cap	0906040G0AADJDJ	3390	Cap	Tab		Colecal_Tab	#N/A	1	0906040G0AAASAS	TRUE
Ergocalcifero	0906040N0AADCDC	800	Oral Susp	Oral Soln	Y	Ergocalcifero	0906040N0AA	2	0906040N0AAFGFG	TRUE
Ergocalcifero	0906040N0AADLDL	460	Liq Spec	Oral Soln	Y	Ergocalcifero	0906040N0AA	2	0906040N0AAFIFI	TRUE
Calc/Vit D_Ta	0906040N0AADXDX	322	Tab Chble	Cap		Calc/Vit D_Ca	#N/A	2	0906040N0AAEJEJ	TRUE
Colecal & Cal	0906040N0AAEEEE	178	Tab	Tab Chble		Colecal & Cal	#N/A	1	0906040N0AAFCFC	TRUE
Ergocalcifero	0906040N0AAEIEI	500	Oral Susp	Oral Soln	Y	Ergocalcifero	0906040N0AA	2	0906040N0AAFJFJ	TRUE
Colecal & Cal	0906040N0AAEXEX	40586	Tab Chble	Tab	Y	Colecal & Cal	0906040G0AA	2	0906040G0AACWCW	TRUE
Ergocalcifero	0906040N0AAFDFD	5100	Oral Soln	Soln		Ergocalcifero	#N/A	2	0906040N0AACHCH	TRUE
Ergocalcifero	0906040N0AAFGFG	2650	Oral Soln	Oral Susp	Y	Ergocalcifero	0906040N0AA	2	0906040N0AADCDC	TRUE
Ergocalcifero	0906040N0AAFIFI	2000	Oral Soln	Liq Spec	Y	Ergocalcifero	0906040N0AA	2	0906040N0AADLDL	TRUE
Ergocalcifero	0906040N0AAFJFJ	3810	Oral Soln	Oral Susp	Y	Ergocalcifero	0906040N0AA	2	0906040N0AAEIEI	TRUE
Ergocalcifero	0906040N0AAFKFK	270	Oral Soln	Oral Susp		Ergocalcifero	#N/A	2	0906040N0AADDDD	TRUE
Vit E_Cap 75	0906050P0AAAAAA	49406	Cap	Gelucap		Vit E_Geluca	#N/A	1	0906050P0AAALAL	TRUE
Vit E_Cap 20	0906050P0AAABAB	54083	Cap	Succ Tab		Vit E_Succ Ta	#N/A	1	0906050P0AAAZAZ	TRUE
Vit E_Cap 40	0906050P0AAAFAF	52601	Cap	Tab		Vit E_Tab 40	#N/A	1	0906050P0AACACA	TRUE
Vit E_Cap 10	0906050P0AAAKAK	399	Cap	Tab		Vit E_Tab 10	#N/A	1	0906050P0AAAGAG	TRUE
Tocoph Acet_	0906050T0AAAFAF	174774	Susp	Liq Spec		Tocoph Acet_	#N/A	1	0906050T0AAAHAH	TRUE
Tocoph Acet_	0906050T0AAAPAP	2602	Tab Chble	Tab	Y	Tocoph Acet_	#N/A	2	0906050T0AAAEAE	TRUE
Menadiol So	0906060L0AAAGAG	1160	Oral Soln	Oral Susp	Y	Menadiol So	0906060L0AA	2	0906060L0AAAPAP	TRUE
Menadiol So	0906060L0AAAPAP	2020	Oral Susp	Oral Soln	Y	Menadiol So	0906060L0AA	2	0906060L0AAAGAG	TRUE
Phytomenad	0906060Q0AAACAC	9885	Tab	Cap	Y	Phytomenad	0906060Q0AA	1	0906060Q0AABABA	TRUE
Phytomenad	0906060Q0AABABA	128	Cap	Tab	Y	Phytomenad	0906060Q0AA	1	0906060Q0AAACAC	TRUE
Levocarnitine	0908010C0AAABAB	160	Tab Chble	Tab	Y	Levocarnitine	#N/A	2	0908010C0AAACAC	TRUE
Sod Benz_Ca	0908010N0AAABAB	784	Cap	Tab	Y	Sod Benz_Ta	0908010N0AA	1	0908010N0AAAXAX	TRUE
Sod Benz_Ta	0908010N0AAAXAX	6472	Tab	Cap	Y	Sod Benz_Ca	0908010N0AA	1	0908010N0AAABAB	TRUE
Sod Benz_Or	0908010N0AABIBI	65475	Oral Soln	Liq		Sod Benz_Liq	#N/A	2	0908010N0AAAAAA	TRUE
Sod Phenylbu	0908010P0AAACAC	450	Cap	Tab	Y	Sod Phenylbu	0908010P0AA	1	0908010P0AAAGAG	TRUE
Sod Phenylbu	0908010P0AAAGAG	9250	Tab	Cap	Y	Sod Phenylbu	0908010P0AA	1	0908010P0AAACAC	TRUE
Betaine Anhy	0908010T0AAAAAA	1628	Tab	Cap		Betaine Anhy	#N/A	1	0908010T0AAABAB	TRUE
Arginine_Cap	091101000AACSCS	1464	Cap	Pdrs		Arginine_Pdr	#N/A	1	091101000AAEGEG	TRUE
Arginine_Tab	091101000AADQDQ	196	Tab	Cap	Y	Arginine_Cap	091101000AA	1	091101000AACSCS	TRUE
Arginine_Ora	091101000AAELEL	37200	Oral Soln	Liq Spec		Arginine_Liq	#N/A	2	091101000AADJDJ	TRUE
Glycine_Pdrs	091101000AAERER	800	Pdrs	Pdr Sach	N	Glycine_Pdr S	091101000AA	1	091101000AAFBFB	TRUE
Glycine_Cap	091101000AAEYEY	2400	Cap	Pdrs		Glycine_Pdrs	#N/A	1	091101000AAESES	TRUE
Glycine_Pdr S	091101000AAFBFB	240	Pdr Sach	Pdrs	N	Glycine_Pdrs	091101000AA	2	091101000AAERER	TRUE
Arginine_Ora	091101000AAFSFS	8900	Oral Soln	Liq Spec		Arginine_Liq	#N/A	2	091101000AADVDV	TRUE
Ubidecareno	091102000AAAIAI	13104	Cap	Tab	Y	Ubidecareno	091102000AA	1	091102000AABMBM	TRUE
Ubidecareno	091102000AABMBM	28	Tab	Cap	Y	Ubidecareno	091102000AA	1	091102000AAAIAI	TRUE
Glucosamine	091200000AADGDG	4138	Tab	Cap	Y	Glucosamine	091200000AA	1	091200000AADJDJ	TRUE
Glucosamine	091200000AADJDJ	2014	Cap	Tab	Y	Glucosamine	091200000AA	1	091200000AADGDG	TRUE
Glucosamine	091200000AADYDY	796	Tab	Cap		Glucosamine	#N/A	1	091200000AAERER	TRUE
Glucosamine	091200000AAEEEE	12180	Cap	Tab	Y	Glucosamine	091200000AA	1	091200000AAELEL	TRUE
Glucosamine	091200000AAELEL	1166	Tab	Cap	Y	Glucosamine	091200000AA	1	091200000AAEEEE	TRUE
Tenoxicam_T	100101040AAAAAA	4075	Tab	Gran Sach		Tenoxicam_G	#N/A	1	100101040AAABAB	TRUE
Meloxicam_T	1001010AAAAAAAA	924340	Tab	Suppos		Meloxicam_S	#N/A	1	1001010AAAAADAD	TRUE
Meloxicam_T	1001010AAAAABAB	856521	Tab	Suppos		Meloxicam_S	#N/A	1	1001010AAAAACAC	TRUE
Ibuprofen Ly	1001010ADAAACAC	6952	Tab	Sach		Ibuprofen Ly	#N/A	1	1001010ADAAADAD	TRUE
Diclofenac So	1001010C0AAADAD	167680	Tab E/C	Suppos	Y	Diclofenac So	1001010C0AA	2	1001010C0AAATAT	TRUE
Diclofenac So	1001010C0AAAEAE	3168802	Tab E/C	Suppos	Y	Diclofenac So	1001010C0AA	2	1001010C0AAAUAU	TRUE
Diclofenac So	1001010C0AAAFAF	41647	Tab	Cap	Y	Diclofenac So	1001010C0AA	1	1001010C0AAANAN	TRUE
Diclofenac So	1001010C0AAALAL	276872	Tab	Cap	Y	Diclofenac So	1001010C0AA	1	1001010C0AAAWAW	TRUE
Diclofenac So	1001010C0AAANAN	33221	Cap	Tab	Y	Diclofenac So	1001010C0AA	1	1001010C0AAAFAF	TRUE
Diclofenac So	1001010C0AAATAT	2645	Suppos	Tab E/C	N	Diclofenac So	1001010C0AA	1	1001010C0AAADAD	TRUE
Diclofenac So	1001010C0AAAUAU	27714	Suppos	Tab E/C	N	Diclofenac So	1001010C0AA	1	1001010C0AAAEAE	TRUE
Diclofenac So	1001010C0AAAWAW	218872	Cap	Tab	Y	Diclofenac So	1001010C0AA	1	1001010C0AAALAL	TRUE
Fenoprofen_	1001010G0AAABAB	356	Tab	Tab Disper		Fenoprofen_	#N/A	1	1001010G0AAACAC	TRUE
Flurbiprofen_	1001010I0AAACAC	14697	Tab	Suppos		Flurbiprofen_	#N/A	1	1001010I0AAAAAA	TRUE
Ibuprofen_Ca	1001010J0AAAAAA	123208	Cap	Capl		Ibuprofen_Ca	#N/A	1	1001010J0AAAHAH	TRUE
Ibuprofen_Ca	1001010J0AAABAB	25760	Cap	Tab		Ibuprofen_Ta	#N/A	1	1001010J0AABLBL	TRUE
Ibuprofen_Ta	1001010J0AAADAD	2098494	Tab	Cap	Y	Ibuprofen_Ca	1001010J0AA	1	1001010J0AAAAAA	TRUE
Ibuprofen_Ta	1001010J0AAAEAE	11897988	Tab	Cap	Y	Ibuprofen_Ca	1001010J0AA	1	1001010J0AAAUAU	TRUE
Ibuprofen_Ta	1001010J0AAAFAF	787906	Tab	Gran Eff Sach	Y	Ibuprofen_G	1001010J0AA	1	1001010J0AAANAN	TRUE
Ibuprofen_G	1001010J0AAANAN	19369	Gran Eff Sach	Tab	N	Ibuprofen_Ta	1001010J0AA	3	1001010J0AAAFAF	TRUE
Ibuprofen_Ca	1001010J0AAAUAU	77671	Cap	Gran Eff Sach		Ibuprofen_G	#N/A	1	1001010J0AAAXAX	TRUE
Ibuprofen_O	1001010J0AABHBH	5682564	Oral Susp	Oral Soln		Ibuprofen_O	#N/A	2	1001010J0AABCBC	TRUE
Ibuprofen_O	1001010J0AABNBN	55059	Orodisper Tab	Cap	Y	Ibuprofen_Ca	1001010J0AA	2	1001010J0AAAAAA	TRUE
Indometacin_	1001010K0AAADAD	103192	Cap	Tab		Indometacin_	#N/A	1	1001010K0AAAJAJ	TRUE
Indometacin_	1001010K0AAAQAQ	2600	Oral Soln	Mix		Indometacin_	#N/A	2	1001010K0AAAEAE	TRUE
Indometacin_	1001010K0AABBBB	12630	Oral Susp	Mix		Indometacin_	#N/A	2	1001010K0AAAEAE	TRUE
Mefenamic A	1001010N0AAAAAA	364237	Cap	Tab		Mefenamic A	#N/A	1	1001010N0AAAEAE	TRUE
Mefenamic A	1001010N0AAABAB	8490	Oral Susp	Liq Spec		Mefenamic A	#N/A	2	1001010N0AAAIAI	TRUE
Naproxen_Ta	1001010P0AAADAD	10976779	Tab	Tab E/C	Y	Naproxen_Ta	1001010P0AA	1	1001010P0AAAHAH	TRUE
Naproxen_Ta	1001010P0AAAEAE	19582658	Tab	Gran Sach		Naproxen_Gr	#N/A	1	1001010P0AAAFAF	TRUE
Naproxen_Ta	1001010P0AAAHAH	1951434	Tab E/C	Tab	Y	Naproxen_Ta	1001010P0AA	2	1001010P0AAADAD	TRUE
Naproxen_Ta	1001010P0AAAIAI	2506811	Tab E/C	Gran Sach		Naproxen_Gr	#N/A	2	1001010P0AAAFAF	TRUE
Naproxen_Ta	1001010P0AAAJAJ	97785	Tab E/C	Tab	Y	Naproxen_Ta	#N/A	2	1001010P0AAAGAG	TRUE
Naproxen_Li	1001010P0AAARAR	5830	Liq Spec	Oral Susp		Naproxen_O	#N/A	2	1001010P0AABABAB	TRUE
Naproxen_O	1001010P0AABCBC	6050	Oral Susp	Liq Spec		Naproxen_Li	#N/A	2	1001010P0AAAXAX	TRUE
Piroxicam_Ca	1001010R0AAAAAA	35137	Cap	Tab Disper		Piroxicam_Ta	#N/A	1	1001010R0AAADAD	TRUE
Piroxicam_Ca	1001010R0AAABAB	30019	Cap	Suppos		Piroxicam_Su	#N/A	1	1001010R0AAACAC	TRUE
Piroxicam_Ta	1001010R0AAAEAE	56	Tab Disper	Cap	N	Piroxicam_Ca	1001010R0AA	2	1001010R0AAABAB	TRUE

Tiaprofenic A	1001010T0AAACAC	9476	Tab	Gran Sach		Tiaprofenic A	#N/A	1	1001010T0AAAAAA	TRUE
Nabumetone	1001010X0AAAAAA	208817	Tab	Tab Disper	N	Nabumetone	#N/A	1	1001010X0AAACAC	TRUE
Hydroxychlor	1001030C0AAAAAA	4872780	Tab	Pdrs		Hydroxychlor	#N/A	1	1001030C0AABGBG	TRUE
Methotrexat	1001030U0AAAHAH	160	Liq Spec	Oral Soln		Methotrexat	#N/A	2	1001030U0AABTBT	TRUE
Allopurinol_T	1001040C0AAABAB	7459650	Tab	Pdr Sach		Allopurinol_P	#N/A	1	1001040C0AAAUAU	TRUE
Allopurinol_C	1001040C0AAALAL	1250	Oral Soln	Oral Susp	Y	Allopurinol_C	1001040C0AA	2	1001040C0AAAXAX	TRUE
Allopurinol_C	1001040C0AAAPAP	3380	Oral Soln	Oral Susp	Y	Allopurinol_C	1001040C0AA	2	1001040C0AAAWAW	TRUE
Allopurinol_C	1001040C0AAAWAW	14370	Oral Susp	Oral Soln	Y	Allopurinol_C	1001040C0AA	2	1001040C0AAAPAP	TRUE
Allopurinol_C	1001040C0AAAXAX	2550	Oral Susp	Oral Soln	Y	Allopurinol_C	1001040C0AA	2	1001040C0AAALAL	TRUE
Glucosamine	1001050A0AAABAB	2108	Tab	Cap		Glucosamine	#N/A	1	1001050A0AAAMAM	TRUE
Glucosamine	1001050A0AAACAC	206	Tab Chble	Tab	Y	Glucosamine	1001050A0AA	2	1001050A0AAAHAH	TRUE
Glucosamine	1001050A0AAAHAH	60	Tab	Tab Chble	Y	Glucosamine	1001050A0AA	1	1001050A0AAACAC	TRUE
Pyridostig Br	1002010Q0AAAIAI	1790	Liq Spec	Oral Soln		Pyridostig Br	#N/A	2	1002010Q0AABFBF	TRUE
Pyridostig Br	1002010Q0AAANAN	3600	Oral Soln	Oral Susp	Y	Pyridostig Br	1002010Q0AA	2	1002010Q0AABHBH	TRUE
Pyridostig Br	1002010Q0AABGBG	7500	Oral Susp	Oral Soln		Pyridostig Br	#N/A	2	1002010Q0AAAMAM	TRUE
Pyridostig Br	1002010Q0AABHBH	1800	Oral Susp	Oral Soln	Y	Pyridostig Br	1002010Q0AA	2	1002010Q0AAANAN	TRUE
Baclofen_Liq	1002020C0AAA1A1	300	Liq Spec	Syr		Baclofen_Syr	#N/A	2	1002020C0AAAUAU	TRUE
Dantrolene S	1002020J0AAARAR	12640	Oral Soln	Mix		Dantrolene S	#N/A	2	1002020J0AAAGAG	TRUE
Dantrolene S	1002020J0AAAUAU	1880	Liq Spec	Susp		Dantrolene S	#N/A	2	1002020J0AAAFAF	TRUE
Dantrolene S	1002020J0AAAVAV	225	Liq Spec	Susp		Dantrolene S	#N/A	2	1002020J0AAAKAK	TRUE
Dantrolene S	1002020J0AABHBH	18430	Oral Susp	Mix		Dantrolene S	#N/A	2	1002020J0AAAGAG	TRUE
Dantrolene S	1002020J0AABIBI	5670	Oral Soln	Oral Susp	Y	Dantrolene S	1002020J0AAI	2	1002020J0AABQBQ	TRUE
Dantrolene S	1002020J0AABQBQ	7405	Oral Susp	Oral Soln	Y	Dantrolene S	1002020J0AAI	2	1002020J0AABIBI	TRUE
Dantrolene S	1002020J0AABRBR	3350	Oral Susp	Oral Soln		Dantrolene S	#N/A	2	1002020J0AAAXAX	TRUE
Tizanidine HC	1002020T0AAAIAI	30900	Oral Soln	Liq Spec		Tizanidine HC	#N/A	2	1002020T0AAADAD	TRUE
Tizanidine HC	1002020T0AAAJAJ	19980	Oral Susp	Liq Spec		Tizanidine HC	#N/A	2	1002020T0AAADAD	TRUE
Dimethyl Sulf	100302040AAAAAA	100	Crm	Ster Soln		Dimethyl Sulf	#N/A	1	0704040F0AAAAAA	TRUE
Ibuprofen_Cr	1003020P0AAAAAA	720	Crm	Gel	Y	Ibuprofen_G	1003020P0AA	1	1003020P0AAACAC	TRUE
Ibuprofen_G	1003020P0AAACAC	9735440	Gel	Crm	Y	Ibuprofen_Cr	1003020P0AA	1	1003020P0AAAAAA	TRUE
Ibuprofen_G	1003020P0AAAIAI	8138830	Gel	Crm		Ibuprofen_Cr	#N/A	1	1003020P0AAABAB	TRUE
Salicylic Acid	1003020W0AAAAAA	1656000	Gel	Crm	Y	Salicylic Acid	1003020W0AA	1	1003020W0AAABAB	TRUE
Salicylic Acid	1003020W0AAABAB	526125	Crm	Gel	Y	Salicylic Acid	1003020W0AA	1	1003020W0AAAAAA	TRUE
Ciprofloxacin	1103010B0AAAAAA	37560	Eye Dps	Ear Dps	N	Ciprofloxacin	1201010ACAA	2	1201010ACAAAAAA	TRUE
Chlorampher	1103010C0AAAAAA	697130	Eye Dps	Eye Oint		Chlorampher	#N/A	2	1103010C0AAACAC	TRUE
Chlorampher	1103010C0AAADAD	170876	Eye Oint	Crm		Chlorampher	#N/A	2	1310011B0AAAAAA	TRUE
Dibromprop	1103010E0AAAAAA	100	Eye Oint	Crm	N	Dibromprop	1310050K0AA	2	1310050K0AAAAAA	TRUE
Gentamicin S	1103010G0AAAFAF	42920	Ear/Eye Dps	Crm		Gentamicin S	#N/A	2	1310012I0AAAAAA	TRUE
Ofloxacin_Ey	1103010Y0AAAAAA	13845	Eye Dps	Ear Dps	N	Ofloxacin_Ea	1201010ABAA	2	1201010ABAAAAAA	TRUE
Betameth So	1104010D0AAABAB	777	Eye Oint	Ear Dps		Betameth So	#N/A	2	1201010E0AAAAAA	TRUE
Betameth So	1104010D0AAAGAG	146690	Ear/Eye/Nsl Dps	Ear Dps		Betameth So	#N/A	2	1201010E0AAAAAA	TRUE
Fluorome_Ey	1104010K0AAAAAA	26340	Eye Dps	Eye Oint		Fluorome_Ey	#N/A	2	1104010K0AAAEAE	TRUE
Prednisolone	1104010S0AABBBB	14480	Ear/Eye Dps	Ear Dps		Prednisolone	#N/A	2	1201010U0AAABAB	TRUE
Prednisolone	1104010S0AABLBL	780	Eye Dps	Ear Dps		Prednisolone	#N/A	2	1104010S0AABHBH	TRUE
Prednisolone	1104010S0AABMBM	190	Eye Dps	Ear Dps		Prednisolone	#N/A	2	1104010S0AABIBI	TRUE
Sod Cromogl	1104020T0AAAAAA	1093506	Eye Dps Aq	Aq Nsl Spy		Sod Cromogl	#N/A	3	1202010P0AAAHAH	TRUE
Atrop Sulf_Ey	1105000B0AAADAD	50	Eye Dps	Eye Oint		Atrop Sulf_Ey	#N/A	2	1105000B0AAAGAG	TRUE
Atrop Sulf_Ey	1105000B0AAAEAE	40760	Eye Dps	Eye Oint	N	Atrop Sulf_Ey	1105000B0AA	2	1105000B0AAAHAH	TRUE
Atrop Sulf_Ey	1105000B0AAAHAH	4	Eye Oint	Eye Dps	N	Atrop Sulf_Ey	1105000B0AA	2	1105000B0AAAEAE	TRUE
Acetazolamic	1106000B0AAA2A2	1600	Liq Spec	Liq	N	Acetazolamic	#N/A	2	1106000B0AAAEAE	TRUE
Acetazolamic	1106000B0AAACAC	342367	Tab	Pdrs		Acetazolamic	#N/A	1	1106000B0AAARAR	TRUE
Acetazolamic	1106000B0AAASAS	1368	Liq Spec	Susp		Acetazolamic	#N/A	2	1106000B0AAAHAH	TRUE
Acetazolamic	1106000B0AABQBQ	4355	Oral Susp	Oral Soln		Acetazolamic	#N/A	2	1106000B0AAATAT	TRUE
Piloc HCl_Eye	1106000X0AAAEAE	9190	Eye Dps	Eye Gel		Piloc HCl_Eye	#N/A	2	1106000X0AABDBD	TRUE
Timolol_Eye	1106000Z0AAAAAA	116455	Eye Dps	Gel Eye Dps	N	Timolol_Gel	1106000Z0AA	2	1106000Z0AAAPAP	TRUE
Timolol_Eye	1106000Z0AAABAB	87020	Eye Dps	Gel Eye Dps	N	Timolol_Gel	1106000Z0AA	2	1106000Z0AAAQAQ	TRUE
Timolol_Gel	1106000Z0AAAPAP	6488	Gel Eye Dps	Eye Dps	N	Timolol_Eye	1106000Z0AA	3	1106000Z0AAAAAA	TRUE
Timolol_Gel	1106000Z0AAAQAQ	2328	Gel Eye Dps	Eye Dps	N	Timolol_Eye	1106000Z0AA	3	1106000Z0AAABAB	TRUE
Ciclosporin_E	1108010AAAAACAC	354	Eye Oint	Eye Dps		Ciclosporin_E	#N/A	2	1108010AAAAAEAE	TRUE
Ciclosporin_E	1108010AAAAAIAI	17	Eye Oint	Eye Dps		Ciclosporin_E	#N/A	2	1108010AAAAAAAA	TRUE
Acetylcy_Eye	1108010C0AAADAD	16070	Eye Dps	Blad Wsht		Acetylcy_Bla	#N/A	2	0704040W0AAAAAA	TRUE
Sod Chlor_Ey	1108010K0AAAAAA	1060	Eye Dps	Eye Irrig		Sod Chlor_Ey	#N/A	2	1108010K0AABIBI	TRUE
Sod Chlor_Ey	1108010K0AAAJAJ	5	Eye Oint	Eye Dps	N	Sod Chlor_Ey	1108010K0AA	2	1108010K0AAAWAW	TRUE
Sod Chlor_Ey	1108010K0AAAQAQ	10	Eye Dps	Ster Soln		Sod Chlor_St	#N/A	2	0902012L0AACECE	TRUE
Sod Chlor_Ey	1108010K0AAAWAW	15	Eye Dps	Eye Oint	N	Sod Chlor_Ey	1108010K0AA	2	1108010K0AAAJAJ	TRUE
Sod Chlor_Ey	1108010K0AACFCF	4105	Eye Oint	Eye Dps		Sod Chlor_Ey	#N/A	2	1108010K0AAABAB	TRUE
Ofloxacin_Ea	1201010ABAAAAAA	5	Ear Dps	Eye Dps	N	Ofloxacin_Ey	1103010Y0AA	2	1103010Y0AAAAAA	TRUE
Ciprofloxacin	1201010ACAAAAAA	417	Ear Dps	Eye Dps	N	Ciprofloxacin	1103010B0AA	2	1103010B0AAAAAA	TRUE
Alum Acet_E	1201010C0AAABAB	10	Ear Dps	Lot		Alum Acet_L	#N/A	2	1311060B0AAANAN	TRUE
Docusate Soc	1201030F0AAACAC	75	Ear Dps	Ear Drop Cap		Docusate Soc	#N/A	2	1201030F0AAAAAA	TRUE
Beclomet Dip	1202010C0AAAAAA	132252	Nsl Spy	Inha B/A		Beclomet Dip	#N/A	2	0302000C0AAASAS	TRUE
Beclomet Dip	1202010C0AAACAC	2	Aq Nsl Spy	Nsl Spy		Beclomet Dip	#N/A	3	1202010C0AAAFAF	TRUE
Fluticasone F	1202010M0AAADAD	14	Nsl Spy	Inha		Fluticasone F	#N/A	2	0302000N0AAAKAK	TRUE
Sod Chlor_Ne	1202020L0AABQBQ	900	Neb Soln	Inh Soln		Sod Chlor_In	#N/A	2	1202020L0AABDBD	TRUE
Sod Chlor_Ne	1202020L0AABZBZ	300	Neb Soln	Eye Dps		Sod Chlor_Ey	#N/A	2	1108010K0AACBCB	TRUE
Mupirocin_N	1202030R0AAAAAA	20856	Nsl Oint	Crm	N	Mupirocin_C	1310011M0AA	2	1310011M0AAABAB	TRUE
Hydrocort_Pa	1203010M0AAABAB	56	Pastil	Cap		Hydrocort_C	#N/A	1	0603020J0AAARAR	TRUE
Triamcinol Ac	1203010T0AAAAAA	246	Oromucosal Past	Crm		Triamcinol Ac	#N/A	2	1304000Z0AAAAAA	TRUE
Doxycycline I	1203010U0AAABAB	5831	Tab	Cap		Doxycycline I	#N/A	1	1203010U0AAAAAA	TRUE
Chlorhex Glu	1203040E0AAABAB	6378262	Mthwsh	Crm		Chlorhex Glu	#N/A	1	1310050J0AAAFAF	TRUE
Chlorhex Glu	1203040E0AAACAC	14400	Mthwsh (Mint)	Crm		Chlorhex Glu	#N/A	2	1310050J0AAAFAF	TRUE
Hydrogen Pe	1203040I0AAADAD	62750	Mthwsh	Crm		Hydrogen Pe	#N/A	1	1311070J0AAAAAA	TRUE
Piloc HCl_Tab	1203050P0AAABAB	81354	Tab	Cap	Y	Piloc HCl_Cap	#N/A	1	1203050P0AAAAAA	TRUE
Cetomacrogo	1301010D0AAAAAA	4951865	Crm (For A) BP	Crm (For B) BP		Cetomacrogo	#N/A	4	1301010D0AAABAB	TRUE
Glycerol_Crm	130201000AACLCL	1730	Crm	Eye Dps		Glycerol_Eye	#N/A	1	1108020L0AAAMAM	TRUE

Dexpanth_Oi	1302010E0AAACAC	1290	Oint	Crm		Dexpanth_Cr	#N/A	1	1302010E0AAABAB	TRUE
Urea_Crm 1C	1302010U0AAAFAF	492470	Crm	Aq Soln		Urea_Aq Solı	#N/A	1	1309000U0AAADAD	TRUE
Urea_Crm 5°	1302010U0AAAKAK	97802	Crm	Face Wsh		Urea_Face W	#N/A	1	1302010U0AAARAR	TRUE
Urea_Lot 10°	1302010U0AAAMAM	409551	Lot	Aq Soln		Urea_Aq Solı	#N/A	1	1309000U0AAADAD	TRUE
Urea_Shamp	1302010U0AAASAS	46000	Shampoo	Crm	N	Urea_Crm 5°	1302010U0AA	1	1302010U0AAAKAK	TRUE
Urea_Scalp A	1302010U0AAAWAW	3000	Scalp Applic	Crm	N	Urea_Crm 5°	1302010U0AA	2	1302010U0AAAKAK	TRUE
Chlorhex Glu	1302010Z0AAAAAA	42000	Emollient/Crm	Crm	Y	Chlorhex Glu	1311020L0AA	1	1310050J0AAABAB	TRUE
Crotamiton_	1303000I0AAAAAA	639290	Crm	Lot	N	Crotamiton_	1303000I0AA	1	1303000I0AAABAB	TRUE
Crotamiton_	1303000I0AAABAB	2550	Lot	Crm	N	Crotamiton_	1303000I0AA	1	1303000I0AAAAAA	TRUE
Lido HCl_Gel	1303000Q0AAAAAA	450	Gel	Mthwsh		Lido HCl_Mth	#N/A	1	1502010J0AADWDW	TRUE
Alclometasoı	1304000B0AAAAAA	8950	Crm	Oint		Alclometasoı	#N/A	1	1304000B0AABABA	TRUE
Beclomet Dip	1304000C0AAAAAA	3450	Crm	Oint	Y	Beclomet Dip	1304000C0AA	1	1304000C0AABABA	TRUE
Beclomet Dip	1304000C0AABABA	3750	Oint	Crm	Y	Beclomet Dip	1304000C0AA	1	1304000C0AAAAAA	TRUE
Betameth Dip	1304000D0AAAAAA	36290	Crm	Oint	Y	Betameth Dip	1304000D0AA	1	1304000D0AABABA	TRUE
Betameth Dip	1304000D0AABABA	40950	Oint	Crm	Y	Betameth Dip	1304000D0AA	1	1304000D0AAAAAA	TRUE
Betameth Dip	1304000D0AABCBC	44640	Scalp Lot	Crm	N	Betameth Dip	1304000D0AA	2	1304000D0AAAAAA	TRUE
Betameth Va	1304000F0AAAAAA	3702431	Crm	Lot	N	Betameth Va	1304000F0AA	1	1304000F0AABCBC	TRUE
Betameth Va	1304000F0AAABAB	1571000	Crm	Oint	Y	Betameth Va	1304000F0AA	1	1304000F0AABBBB	TRUE
Betameth Va	1304000F0AABABA	2301460	Oint	Crm	Y	Betameth Va	1304000F0AA	1	1304000F0AAAAAA	TRUE
Betameth Va	1304000F0AABBBB	946000	Oint	Crm	Y	Betameth Va	1304000F0AA	1	1304000F0AAABAB	TRUE
Betameth Va	1304000F0AABCBC	62600	Lot	Crm	Y	Betameth Va	1304000F0AA	1	1304000F0AAAAAA	TRUE
Betameth Va	1304000F0AABDBD	4143400	Scalp Applic	Crm	N	Betameth Va	1304000F0AA	2	1304000F0AAAAAA	TRUE
Betameth Va	1304000F0AACACA	67050	Crm	Oint	Y	Betameth Va	1304000F0AA	1	1304000F0AACDCD	TRUE
Betameth Va	1304000F0AACBCB	57710	Crm	Lot		Betameth Va	#N/A	1	1304000F0AACFCF	TRUE
Betameth Va	1304000F0AACDCD	50340	Oint	Crm	Y	Betameth Va	1304000F0AA	1	1304000F0AACACA	TRUE
Betameth Va	1304000F0AACECE	32920	Oint	Crm		#VALUE!	#VALUE!	0	#VALUE!	TRUE
Clobetasol Pı	1304000G0AAAAAA	1139340	Crm	Oint	Y	Clobetasol Pı	1304000G0AA	1	1304000G0AABABA	TRUE
Clobetasol Pı	1304000G0AABABA	1319720	Oint	Crm	Y	Clobetasol Pı	1304000G0AA	1	1304000G0AAAAAA	TRUE
Clobetasol Pı	1304000G0AABBBB	229170	Scalp Applic	Crm	N	Clobetasol Pı	1304000G0AA	2	1304000G0AAAAAA	TRUE
Clobet But_C	1304000H0AAAAAA	1704530	Crm	Oint	Y	Clobet But_C	1304000H0AA	1	1304000H0AABABA	TRUE
Clobet But_C	1304000H0AABABA	1791110	Oint	Crm	Y	Clobet But_C	1304000H0AA	1	1304000H0AAAAAA	TRUE
Diflucortolor	1304000L0AAAAAA	3450	Crm	Fatty Oint		Diflucortolor	#N/A	1	1304000L0AABABA	TRUE
Diflucortolor	1304000L0AAABAB	7110	Oily Crm	Crm	Y	Diflucortolor	1304000L0AA	2	1304000L0AAAAAA	TRUE
Diflucortolor	1304000L0AABBBB	1470	Oint	Crm	Y	Diflucortolor	1304000L0AA	1	1304000L0AAAAAA	TRUE
Fluocinolone	1304000N0AAABAB	26050	Crm	Gel	Y	Fluocinolone	1304000N0AA	1	1304000N0AABDBD	TRUE
Fluocinolone	1304000N0AAADAD	23350	Crm	Oint	Y	Fluocinolone	1304000N0AA	1	1304000N0AABCBC	TRUE
Fluocinolone	1304000N0AABBBB	36600	Oint	Crm	Y	Fluocinolone	1304000N0AA	1	1304000N0AAABAB	TRUE
Fluocinolone	1304000N0AABCBC	22000	Oint	Crm	Y	Fluocinolone	1304000N0AA	1	1304000N0AAADAD	TRUE
Fluocinolone	1304000N0AABDBD	75780	Gel	Crm	Y	Fluocinolone	1304000N0AA	1	1304000N0AAABAB	TRUE
Fluocinolone	1304000N0AACACA	5565	Crm	Oint	Y	Fluocinolone	1304000N0AA	1	1304000N0AACCCC	TRUE
Fluocinolone	1304000N0AACBCB	4440	Crm	Oint	Y	Fluocinolone	1304000N0AA	1	1304000N0AACDCD	TRUE
Fluocinolone	1304000N0AACCCC	3000	Oint	Crm	Y	Fluocinolone	1304000N0AA	1	1304000N0AACACA	TRUE
Fluocinolone	1304000N0AACDCD	3330	Oint	Crm	Y	Fluocinolone	1304000N0AA	1	1304000N0AACBCB	TRUE
Fluocinonide	1304000P0AAAAAA	20825	Crm	Oint	Y	Fluocinonide	1304000P0AA	1	1304000P0AABABA	TRUE
Fluocinonide	1304000P0AABABA	27525	Oint	Crm	Y	Fluocinonide	1304000P0AA	1	1304000P0AAAAAA	TRUE
Fludroxycort	1304000T0AAAAAA	12900	Crm	Oint	Y	Fludroxycort	1304000T0AA	1	1304000T0AABABA	TRUE
Fludroxycort	1304000T0AABABA	15300	Oint	Crm	Y	Fludroxycort	1304000T0AA	1	1304000T0AAAAAA	TRUE
Hydrocort_C	1304000V0AAACAC	342045	Crm	Ear Dps		Hydrocort_E	#N/A	1	1201010Q0AAABAB	TRUE
Hydrocort_C	1304000V0AAADAD	3596865	Crm	Ear Dps		Hydrocort_E	#N/A	1	1201010Q0AAAAAA	TRUE
Hydrocort_C	1304000V0AAAFAF	67875	Crm	Eye Oint		Hydrocort_E	#N/A	1	1104010M0AAAEAE	TRUE
Hydrocort_C	1304000V0AAAWAW	88440	Crm	Eye Oint		Hydrocort_E	#N/A	1	1104010M0AAAMAM	TRUE
Hydrocort_O	1304000V0AABBBB	69240	Oint	Crm	Y	Hydrocort_C	1304000V0AA	1	1304000V0AAACAC	TRUE
Hydrocort_O	1304000V0AABCBC	1205800	Oint	Crm	Y	Hydrocort_C	1304000V0AA	1	1304000V0AAADAD	TRUE
Hydrocort_O	1304000V0AABDBD	23940	Oint	Crm	Y	Hydrocort_C	1304000V0AA	1	1304000V0AAAFAF	TRUE
Hydrocort/M	1304000V0AACHCH	3274380	Crm	Oint	Y	Hydrocort/M	1304000V0AA	1	1304000V0AACSCS	TRUE
Hydrocort/M	1304000V0AACSCS	773820	Oint	Crm	Y	Hydrocort/M	1304000V0AA	1	1304000V0AACHCH	TRUE
Hydrocort Bu	1304000W0AAAAAA	90910	Crm	Emollient Crm		Hydrocort Bu	#N/A	1	1304000W0AAABAB	TRUE
Hydrocort Bu	1304000W0AABABA	23010	Oint	Crm	Y	Hydrocort Bu	1304000W0A	1	1304000W0AAAAAA	TRUE
Hydrocort Bu	1304000W0AABBBB	29000	Scalp Lot	Crm	N	Hydrocort Bu	1304000W0A	2	1304000W0AAAAAA	TRUE
Hydrocort Bu	1304000W0AABDBD	3400	Emuls	Crm	Y	Hydrocort Bu	1304000W0A	1	1304000W0AAAAAA	TRUE
Hydrocort Ac	1304000X0AAAAAA	3375	Crm	Ear Dps		Hydrocort Ac	#N/A	1	1201010G0AAAEAE	TRUE
Hydrocort Ac	1304000X0AABABA	225	Oint	Crm	Y	Hydrocort Ac	1304000X0AA	1	1304000X0AAAAAA	TRUE
Hydrocort Ac	1304000X0AACBCB	1149450	Crm	Gel		Hydrocort Ac	#N/A	1	1304000X0AACICI	TRUE
Mometasone	1304000Y0AAAAAA	1307170	Crm	Oint	Y	Mometasone	1304000Y0AA	1	1304000Y0AABABA	TRUE
Mometasone	1304000Y0AABABA	1762890	Oint	Crm	Y	Mometasone	1304000Y0AA	1	1304000Y0AAAAAA	TRUE
Mometasone	1304000Y0AABBBB	71400	Scalp Lot	Crm	N	Mometasone	1304000Y0AA	2	1304000Y0AAAAAA	TRUE
Coal Tar_Oin	1305020C0AAAVAV	3000	Oint	Crm		Coal Tar_Crm	#N/A	1	1305020C0AABVBV	TRUE
Coal Tar_Oin	1305020C0AABSBS	2200	Oint	Crm		Coal Tar_Crm	#N/A	1	1305020C0AACBCB	TRUE
Calcipotriol_	1305020D0AAAAAA	1556520	Oint	Crm	Y	Calcipotriol_	1305020D0AA	1	1305020D0AAABAB	TRUE
Calcipotriol_	1305020D0AAABAB	1320	Crm	Oint	Y	Calcipotriol_	1305020D0AA	1	1305020D0AAAAAA	TRUE
Calcipotriol/I	1305020D0AAAFAF	1543050	Oint	Gel	Y	Calcipotriol/I	1305020D0AA	1	1305020D0AAAGAG	TRUE
Calcipotriol/I	1305020D0AAAGAG	1088100	Gel	Oint	Y	Calcipotriol/I	1305020D0AA	1	1305020D0AAAFAF	TRUE
Dithranol_Cr	1305020F0AABKBK	6300	Crm	Oint		Dithranol_Oi	#N/A	1	1305020F0AACICI	TRUE
Dithranol_Cr	1305020F0AABMBM	3750	Crm	Lipid Crm		Dithranol_Lip	#N/A	1	1305020F0AAEAEA	TRUE
Dithranol_Cr	1305020F0AACZCZ	13250	Crm	Oint		Dithranol_Oi	#N/A	1	1305020F0AABNBN	TRUE
Dithranol_Cr	1305020F0AADADA	6200	Crm	Oint		Dithranol_Oi	#N/A	1	1305020F0AABQBQ	TRUE
Dithranol_Cr	1305020F0AADBDB	3700	Crm	Oint		Dithranol_Oi	#N/A	1	1305020F0AACUCU	TRUE
Dithranol_Cr	1305020F0AADTDT	1150	Crm	Lipid Crm		Dithranol_Lip	#N/A	1	1305020F0AAEBEB	TRUE
Methoxsalen	1305020L0AAAJAJ	28	Tab	Cap		Methoxsalen	#N/A	1	1305020L0AAAEAE	TRUE
Tacalcitol_Oi	1305020R0AAAAAA	23030	Oint	Lot	Y	Tacalcitol_Lo	1305020R0AA	1	1305020R0AAABAB	TRUE
Tacalcitol_Lo	1305020R0AAABAB	2970	Lot	Oint	Y	Tacalcitol_Oi	1305020R0AA	1	1305020R0AAAAAA	TRUE
Salic Acid_Cr	1305020S0AAA4A4	3400	Crm	Collod		Salic Acid_Co	#N/A	1	1307000M0AAAKAK	TRUE
Salic Acid_Oi	1305020S0AAABAB	117140	Oint	Collod		Salic Acid_Co	#N/A	1	1307000M0AAARAR	TRUE
Salic Acid_Lo	1305020S0AAAEAE	500	Lot	Collod		Salic Acid_Co	#N/A	1	1307000M0AAARAR	TRUE

Tacrolimus_(	1305030C0AAACAC	92700	Oint	Oral Gel		Tacrolimus_(	#N/A	1	0802020T0AAAUAU	TRUE
Benzoyl Per_	1306010C0AAAAAA	1720	Gel	Crm		Benzoyl Per_	#N/A	1	1306010C0AAAZAZ	TRUE
Benzoyl Per_	1306010C0AAABAB	349180	Gel	Crm	Y	Benzoyl Per_	1306010C0AA	1	1306010C0AAADAD	TRUE
Benzoyl Per_	1306010C0AAACAC	17560	Gel	A-Bact Skin W:	Y	Benzoyl Per_	1306010C0AA	1	1306010C0AAAJAJ	TRUE
Benzoyl Per_	1306010C0AAADAD	4040	Crm	Gel	Y	Benzoyl Per_	1306010C0AA	1	1306010C0AAABAB	TRUE
Benzoyl Per_	1306010C0AAAJAJ	6450	A-Bact Skin Wsh	Crm		Benzoyl Per_	#N/A	3	1306010C0AAAKAK	TRUE
Clindamycin	1306010F0AAABAB	166320	Lot	Gel	N	Clindamycin	1306010F0AA	1	1306010F0AAADAD	TRUE
Clindamycin	1306010F0AAADAD	52830	Gel	Lot	N	Clindamycin	1306010F0AA	1	1306010F0AAABAB	TRUE
Adapalene_(	1306010H0AAAAAA	309285	Gel	Crm	Y	Adapalene_(	1306010H0AA	1	1306010H0AAABAB	TRUE
Adapalene_(	1306010H0AAABAB	243315	Crm	Gel	N	Adapalene_(	1306010H0AA	1	1306010H0AAAAAA	TRUE
Erythromycir	1306010I0AAAAAA	600	Top Soln	Gel		Erythromycir	#N/A	2	1306010I0AAADAD	TRUE
Tretinoin_Ge	1306010V0AAABAB	240	Gel	Crm	Y	Tretinoin_Cri	1306010V0AA	1	1306010V0AAAEAE	TRUE
Tretinoin_Cri	1306010V0AAAEAE	61	Crm	Gel	Y	Tretinoin_Ge	1306010V0AA	1	1306010V0AAABAB	TRUE
Isotretinoin_	1306020J0AAABAB	116282	Cap	Tab		Isotretinoin_	#N/A	1	1306020J0AAADAD	TRUE
Formaldehyc	1307000C0AAABAB	90	Soln Gel	Soln	N	Formaldehyc	#N/A	2	1307000C0AABHBH	TRUE
Formaldehyc	1307000C0AAAFAF	942	Soln	Lot		Formaldehyc	#N/A	1	1307000C0AAAAAA	TRUE
Formaldehyc	1307000C0AAALAL	2000	Buff Soln	Lot		Formaldehyc	#N/A	2	1307000C0AAAGAG	TRUE
Glutaraldehy	1307000F0AAAAAA	3050	Soln	Gel		Glutaraldehy	#N/A	1	1307000F0AAABAB	TRUE
Salic Acid_Oi	1307000M0AAAEAE	3735	Oint	Collod		Salic Acid_Cc	#N/A	1	1307000M0AAA9A9	TRUE
Salic Acid_So	1307000M0AABABA	20330	Soln	Collod		Salic Acid_Cc	#N/A	1	1307000M0AABJBJ	TRUE
Salic Acid_Ge	1307000M0AABMBM	8230	Gel	Collod		Salic Acid_Cc	#N/A	1	1307000M0AABJBJ	TRUE
Salic Acid_M	1307000M0AABSBS	397	Medic Plastr	Collod		Salic Acid_Cc	#N/A	2	1307000M0AAAFAF	TRUE
Salic Acid_Oi	1307000M0AABVBV	7800	Oint	Collod		Salic Acid_Cc	#N/A	1	1307000M0AAAGAG	TRUE
Caustic_Appl	1307000Q0AAAEAE	679	Applic	Point		Caustic_Poin	#N/A	1	1307000Q0AAAFAF	TRUE
Coal Tar_Ext	1309000C0AAANAN	1476625	Ext Shampoo	Emuls		Coal Tar_Emi	#N/A	2	1305020C0AABLBL	TRUE
Coal Tar_Ext	1309000C0AAATAT	444000	Ext Shampoo	Crm		Coal Tar_Crm	#N/A	2	1305020C0AABVBV	TRUE
Minoxidil_So	1309000H0AAAAAA	60	Soln	Gel	Y	Minoxidil_Ge	1309000H0AA	1	1309000H0AAAKAK	TRUE
Minoxidil_Ge	1309000H0AAAKAK	60	Gel	Lot		Minoxidil_Lo	#N/A	1	1309000H0AAABAB	TRUE
Minoxidil_Fo	1309000H0AAALAL	17580	Foam Aero	Lot		Minoxidil_Lo	#N/A	2	1309000H0AAAIAI	TRUE
Ketoconazol(	1309000I0AAAAAA	8113920	Shampoo	Crm	N	Ketoconazol(	1310020L0AA	1	1310020L0AAAAAA	TRUE
Benzalk Chlo	1309000L0AAABAB	334500	Shampoo	Gel		Benzalk Chlo	#N/A	1	1309000L0AAAAAA	TRUE
Selenium Sul	1309000S0AAABAB	749100	Shampoo	Crm		Selenium Sul	#N/A	1	1309000S0AAACAC	TRUE
Mupirocin_C	1310011M0AAAAAA	69135	Oint	Crm	N	Mupirocin_C	1310011M0A/	1	1310011M0AAABAB	TRUE
Mupirocin_C	1310011M0AAABAB	25215	Crm	Nsl Oint	N	Mupirocin_N	1202030R0AA	1	1202030R0AAAAAA	TRUE
Neomycin Su	1310011P0AAAAAA	165	Crm	Ear Dps		Neomycin Su	#N/A	1	1201010T0AAAAAA	TRUE
Fusidic Acid_	1310012F0AAABAB	1881930	Crm	Caviject		Fusidic Acid_	#N/A	1	1310012F0AAAAAA	TRUE
Fusidic Acid_	1310012F0AAACAC	30	Gel	Caviject		Fusidic Acid_	#N/A	1	1310012F0AAAAAA	TRUE
Metronidazo	1310012K0AAAQAQ	225	Gel	Crm		Metronidazo	#N/A	1	1310012K0AAAFAF	TRUE
Metronidazo	1310012K0AAARAR	386910	Gel	Crm	N	Metronidazo	1310012K0AA	1	1310012K0AAAXAX	TRUE
Metronidazo	1310012K0AAAXAX	249440	Crm	Gel	Y	Metronidazo	1310012K0AA	1	1310012K0AAARAR	TRUE
Terbinafine I	131002030AAAAAA	687417	Crm	Gel	Y	Terbinafine I	131002030AA	1	131002030AAACAC	TRUE
Terbinafine I	131002030AAACAC	4200	Gel	Crm	Y	Terbinafine I	131002030AA	1	131002030AAAAAA	TRUE
Terbinafine I	131002030AAADAD	896	Soln	Crm	Y	Terbinafine I	131002030AA	1	131002030AAAAAA	TRUE
Clotrimazole	1310020H0AAAAAA	42360	Soln	Crm	Y	Clotrimazole	1310020H0AA	1	1310020H0AAABAB	TRUE
Clotrimazole	1310020H0AAABAB	2486105	Crm	Eye Dps		Clotrimazole	#N/A	1	1103020C0AAAAAA	TRUE
Econazole Ni	1310020J0AAAAAA	6360	Crm	Lot		Econazole Ni	#N/A	1	1310020J0AAABAB	TRUE
Ketoconazol(	1310020L0AAAAAA	202590	Crm	Shampoo	N	Ketoconazol(	1309000I0AA/	1	1309000I0AAAAAA	TRUE
Miconazole I	1310020N0AAAAAA	1366140	Crm	Dust Pdr	N	Miconazole I	1310020N0AA	1	1310020N0AAABAB	TRUE
Miconazole I	1310020N0AAABAB	50360	Dust Pdr	Crm	N	Miconazole I	1310020N0AA	2	0702020P0AAAFAF	TRUE
Nystatin_Crn	1310020U0AAAAAA	60	Crm	Dust Pdr		Nystatin_Dus	#N/A	1	1310020U0AAABAB	TRUE
Tolnaftate_D	1310020Y0AAABAB	1200	Dust Pdr	Crm		Tolnaftate_C	#N/A	2	1310020Y0AAAAAA	TRUE
Malathion_A	1310040M0AAACAC	200	Alcoholic Lot	Aq Lot	Y	Malathion_A	1310040M0A/	2	1310040M0AAADAD	TRUE
Malathion_A	1310040M0AAADAD	509200	Aq Lot	Alcoholic Lot	Y	Malathion_A	1310040M0A/	2	1310040M0AAACAC	TRUE
Dimeticone_	1310040V0AAAAAA	249550	Lot	Crm		Dimeticone_	#N/A	1	1302020D0AAAJAJ	TRUE
Dimeticone_	1310040V0AAAEAE	53	Soln Spy	Lot Spy		Dimeticone_	#N/A	2	1310040V0AAADAD	TRUE
Cetrimide_Cr	1310050D0AAAAAA	7160	Crm	Soln		Cetrimide_Sc	#N/A	1	1311030G0AAAKAK	TRUE
Hydrogen Pe	1310050H0AAAAAA	10425	Crm	Lipid Crm		Hydrogen Pe	#N/A	1	1310050H0AAABAB	TRUE
Chlorhex Glu	1310050J0AAAAAA	510	Clr Gel	Soln	N	Chlorhex Glu	1311020L0AA	2	1311020L0AAALAL	TRUE
Dibromprop	1310050K0AAAAAA	650	Crm	Eye Oint	N	Dibromprop	1103010E0AA	1	1103010E0AAAAAA	TRUE
Ims_70%	1311010A0AAADAD	3600		Soln		#VALUE!	#VALUE!	0	#VALUE!	TRUE
Isopropyl Alc	1311010I0AAABAB	4700		Pre-Inj Swab		#VALUE!	#VALUE!	0	#VALUE!	TRUE
Sod Chlor_Sc	1311010S0AAADAD	5100	Soln	Eye Dps	Y	Sod Chlor_Ey	1108010K0AA	1	1108010K0AAAAAA	TRUE
Chlorhex Glu	1311020L0AAAEAE	450	Cleansing Lot	Soln		Chlorhex Glu	#N/A	2	1311020L0AABPBP	TRUE
Chlorhex Glu	1311020L0AAAFAF	10750	Crm	Emollient/Crm	Y	Chlorhex Glu	1302010Z0AA	1	1302010Z0AAAAAA	TRUE
Chlorhex Glu	1311020L0AAAKAK	39200	Soln Conc	Crm		Chlorhex Glu	#N/A	2	1310050J0AAAGAG	TRUE
Chlorhex Glu	1311020L0AAALAL	57425	Soln	Clr Gel	N	Chlorhex Glu	1310050J0AA/	1	1310050J0AAAAAA	TRUE
Chlorhex Glu	1311020L0AAANAN	44400	Soln	Crm	Y	Chlorhex Glu	1311020L0AA	1	1310050J0AAABAB	TRUE
Chlorhex Glu	1311020L0AAAPAP	4	Soln	Eye Dps		Chlorhex Glu	#N/A	1	110301020AAABAB	TRUE
Povidone-Ioc	1311040K0AAAFAF	8500	Alcoholic Soln	Antis Soln		Povidone-Ioc	#N/A	2	1311040K0AAAAAA	TRUE
Povidone-Ioc	1311040K0AAAKAK	32500	Surg Scrub	Scalp/Skin Cleanser		Povidone-Ioc	#N/A	2	1311040K0AAAJAJ	TRUE
Povidone-Ioc	1311040K0AAATAT	60590	Soln	Alcoholic Soln	N	Povidone-Ioc	1311040K0AA	1	1311040K0AAAFAF	TRUE
Sod Hypochl(	1311040T0AABABA	10000	Soln	Sterilising Soln		Sod Hypochl(	#N/A	1	1311040T0AAACAC	TRUE
Triclosan_Liq	1311050U0AAAIAI	67700	Liq	Crm		Triclosan_Cri	#N/A	1	1311050U0AAAEAE	TRUE
Glycopyrroni	1312000G0AAAMAM	531	Crm	Oint		Glycopyrroni	#N/A	1	1312000G0AAAEAE	TRUE
Glycopyrroni	1312000G0AAAUAU	200	Aq Crm	Crm		Glycopyrroni	#N/A	2	1312000G0AAANAN	TRUE
Glycopyrroni	1312000G0AABCBC	17000	Top Soln	Crm		Glycopyrroni	#N/A	2	1312000G0AAAYAY	TRUE
Heparinoid_(	1314000H0AAAAAA	264750	Crm	Gel	Y	Heparinoid_(	1314000H0AA	1	1314000H0AAABAB	TRUE
Heparinoid_(	1314000H0AAABAB	129400	Gel	Crm	Y	Heparinoid_(	1314000H0AA	1	1314000H0AAAAAA	TRUE
Hydroquinon	1315000G0AAAWAW	100	Crm	Oint		Hydroquinon	#N/A	1	1315000G0AAARAR	TRUE
Rabies_Vac I	1404000N0AAAAAA	72	Vac Inact (HDC)	Vac Inact (PCE(	N	Rabies_Vac I	1404000N0AA	3	1404000N0AAABAB	TRUE
Rabies_Vac I	1404000N0AAABAB	38	Vac Inact (PCEC)	Vac Inact (HDC	N	Rabies_Vac I	1404000N0AA	3	1404000N0AAAAAA	TRUE
Meningoc_V	1404000X0AAAHAH	4	Vac Group B	Vac C		Meningoc_V	#N/A	3	1404000X0AAAFAF	TRUE
Cocaine_Mth	1502010G0AAADAD	2700	Mthwsh	Eye Dps		Cocaine_Eye	#N/A	1	1107000F0AAADAD	TRUE
Lido_Oint 5%	1502010I0AAAEAE	68160	Oint	Medic Plastr	N	Lido_Medic I	1502010J0AAI	1	1502010J0AAELEL	TRUE

Lido HCl_Top	1502010J0AAAKAK	40	Top Soln	Gel		Lido HCl_Gel	#N/A	2	1502010J0AABPBP	TRUE
Lido HCl_Inj :	1502010J0AABDBD	8249	Inj	Anhy Inj		Lido HCl_Anh	#N/A	1	1502010J0AAAQAQ	TRUE
Lido HCl_Inj :	1502010J0AABEBE	13662	Inj	Anhy Inj		Lido HCl_Anh	#N/A	1	1502010J0AAARAR	TRUE
Lido HCl_Gel	1502010J0AABMBM	85	Gel	Mthwsh		Lido HCl_Mth	#N/A	1	1502010J0AAEFEF	TRUE
Lido HCl/Prilc	1502010J0AABYBY	98853	Crm	Skin Patch		Lido HCl/Prilc	#N/A	1	1502010J0AADZDZ	TRUE
Lido_Medic F	1502010J0AAELEL	579615	Medic Plastr	Oint	N	Lido_Oint 5%	1502010I0AA/	2	1502010I0AAAEAE	TRUE
Lido HCl_Gel	1502010J0AAEPEP	1565	Gel	Antis Gel (S)		Lido HCl_Ant	#N/A	1	1502010J0AACSCS	TRUE
Ammon Sulf_	190500000AAACAC	224	Cap	Tab		Ammon Sulf_	#N/A	1	190500000AABKBK	TRUE
Acetic Acid_S	190600000AAA9A9	1000	Soln	Ear Dps		Acetic Acid_E	#N/A	1	1201010B0AAAEAE	TRUE
Peppermint_	190601000AAAKAK	5114	Water Conc BP	Water BP	N	Peppermint_	190601000AA	3	190601000AAALAL	TRUE
Peppermint_	190601000AAALAL	64800	Water BP	Water Conc BF	N	Peppermint_	190601000AA	2	190601000AAAKAK	TRUE
				Tab						TRUE
				Tab						TRUE
				Tab Chble						TRUE
				Pdrs						TRUE
				Tab						TRUE
				Tab						TRUE
				Susp						TRUE
				Susp						TRUE
				Oral Susp						TRUE
				Oral Soln						TRUE
				Oral Susp						TRUE
				Oral Soln						TRUE
				Oral Susp						TRUE
				Oral Soln						TRUE
				Oral Susp						TRUE
				Oral Soln						TRUE
				Mthwsh						TRUE
				Oral Susp						TRUE
				Mthwsh						TRUE
				Oral Soln						TRUE
				Oral Susp						TRUE
				Oral Soln						TRUE
				Disper Tab						TRUE
				Tab Eff						TRUE
				Disper Tab						TRUE
				Tab						TRUE
				Susp						TRUE
				Oral Susp						TRUE
				Oral Soln						TRUE
				Tab						TRUE
				Tab E/C						TRUE
				Cap E/C						TRUE
				Tab E/C						TRUE
				Tab						TRUE
				Tab E/C						TRUE
				Tab						TRUE
				Tab						TRUE
				Oint						TRUE
				Gel						TRUE
				Syr						TRUE
				Syr						TRUE
				Pdrs						TRUE
				Susp						TRUE
				Syr						TRUE
				Susp						TRUE
				Syr						TRUE
				Oral Susp						TRUE
				Susp						TRUE
				Syr						TRUE
				Susp						TRUE
				Syr						TRUE
				Syr						TRUE
				Syr						TRUE
				Syr						TRUE
				Susp						TRUE
				Susp						TRUE
				Syr						TRUE
				Syr						TRUE
				Syr						TRUE
				Syr						TRUE
				Susp						TRUE
				Susp						TRUE
				Susp						TRUE
				Susp						TRUE
				Susp						TRUE
				Oral Susp						TRUE
				Oral Soln						TRUE
				Oral Susp						TRUE
				Oral Soln						TRUE
				Oral Susp						TRUE
				Oral Soln						TRUE
				Oral Susp						TRUE
				Oral Soln						TRUE

Oral Susp	TRUE
Susp	TRUE
Oral Soln	TRUE
Susp	TRUE
Pdrs	TRUE
Susp	TRUE
Mix	TRUE
Oral Soln	TRUE
Susp	TRUE
Oral Soln	TRUE
Susp	TRUE
Susp	TRUE
Syr	TRUE
Oral Susp	TRUE
Oral Soln	TRUE
Oral Susp	TRUE
Oral Soln	TRUE
Oral Susp	TRUE
Susp	TRUE
Oral Susp	TRUE
Pdrs	TRUE
Wafer	TRUE
Oral Susp	TRUE
Oral Soln	TRUE
Oral Susp	TRUE
Oral Susp	TRUE
Oral Susp	TRUE
Oral Soln	TRUE
Oral Susp	TRUE
Oral Soln	TRUE
Oral Susp	TRUE
Oral Susp	TRUE
Oral Susp	TRUE
Oral Susp	TRUE
Pdrs	TRUE
Tab	TRUE
Pdr Sach	TRUE
Suppos	
Tab Chble	
Tab E/C	
Pdr Sach	
Suppos	
Tab	
Tab Chble	
Oral Susp	
Oral Soln	
Susp	
Oral Susp	
Oral Soln	
Inha B/A	
Nsl Spy	
Suppos	
Oral Soln	
Pdrs	
Pdrs	
Tab	
Tab Eff	
Tab Eff	
Syr	
Liq Spec	
Oral Susp	
Loz Subling	
Orodisper Tab	
Tab Disper	
Tab Subling	
Orodisper Tab	
Tab	
Tab Disper	
Tab Subling	
Oral Soln	
Oral Susp	
Oral Soln	
Oral Susp	
Oral Soln	
Oral Susp	
Oral Soln	
Oral Susp	
Syr	
Oral Soln	
Syr	
Liq Spec	
Syr	
Pdrs	
Suppos	

Oral Susp
Oral Soln
Oral Soln
Oral Susp
Oral Susp
Susp
Suppos
Suppos
Suppos
Susp
Tab Sublingual
Oral Susp
Susp
Oral Soln
Susp
Oral Susp
Susp
Oral Soln
Susp
Oral Susp
Suppos
Tab
Oral Soln
Oral Susp
Susp
Oral Susp
Tab
Oral Susp
Syr
Suppos
Oral Soln
Susp
Syr
Oral Soln
Oral Susp
Syr
Oral Susp
Susp
Syr
Mix
Oral Susp
Syr
Syr
Oral Susp
Oral Soln
Oral Susp
Oral Soln
Oral Soln
Oral Susp
Oral Susp
Susp
Syr
Oral Soln
Susp
Syr
Suppos
Oral Susp
Syr
Oral Soln
Syr
Oral Susp
Oral Soln
Oral Susp
Oral Soln
Suppos
Tab
Tab Eff Solb
Tab Solb
Suppos
Tab E/C
Tab Eff Solb
Tab Solb
Suppos
Tab Eff
Tab
Tab Eff
Tab
Suppos
Syr
Pdrs
Suppos
Tab
Tab Solb
Tab Spec Fine Grade

Syr
Syr
Syr
Capl
Pdrs
Suppos
Tab Solb
Tab Spec Fine Grade
Capl
Pdrs
Suppos
Tab
Tab Spec Fine Grade
Pdrs
Suppos
Tab
Syr
Pdrs
Solb Gran Sach
Tab
Tab Solb
Pdrs
Tab
Tab Solb
Pdrs
Capl
Pdrs
Tab
Tab Solb
Tab Spec Fine Grade
Rapid Tab
Tab
Tab
Oral Susp
Syr
Syr
Suppos
Tab
Solb Gran Sach
Suppos
Tab
Tab Solb
Pdrs
Solb Gran Sach
Suppos
Tab Solb
Tab Buccal
Tab Buccal
Tab Buccal
Tab Sublingual
Tab Sublingual
Tab Sublingual
Suppos
Suppos
Tab
Suppos
Tab Disper
Suppos
Oral Soln
Suppos
Suppos
Tab
Suppos
Suppos
Gran Sach
Gran Sach
Gran Sach
Gran Sach
Tab
Tab
Tab
Tab
Tab
Tab
Tab
Tab
Tab
Suppos
Oral Susp
Oral Soln
Tab G/R
Pdrs
Pdrs
Tab

Tab
Oral Susp
Susp
Syr
Oral Susp
Susp
Pdrs
Suppos
Tab Chble
Tab
Pdrs
Syr
Oral Soln
Oral Susp
Susp
Syr
Syr
Oral Susp
Oral Susp
Oral Susp
Tab Disper
Liq Spec
Pdrs
Pdrs
Suppos
Pdrs
Oral Soln
Oral Susp
Soln
Soln
Liq
Mix
Sod Tab
Suppos
Sod Clear Cap
Suppos
Sod Pdrs
Sod Tab
Suppos
Tab Chble
Suppos
Suppos
Tab
Tab
Sod Clear Cap
Sod Pdrs
Sod Tab
Suppos
Oral Soln
Oral Susp
Susp
Loz
Subling Tab
Subling Tab
Tab Chble
Tab Disp
Tab Chble
Tab Disper
Tab Chble
Tab Chble
Tab
Pdr Sach
Tab
Cap E/C
Ethylsuc Cap
Ethylsuc Sach
Ethylsuc Tab
Suppos
Ethylsuc Cap
Ethylsuc Sach
Ethylsuc Tab
Suppos
Tab E/C
Ethylsuc Sach
Tab E/C
Susp
Syr
Syr
Syr
Tab
Pdrs
Inf(Sod Cholesteryl)
Oral Susp Add Free
Mix

Tab Uncoated
Mix
Susp
Syr
Inj (Hum Prb)
Inj (Hum Pyr)
Inj (Pore Mc)
Inj (Pore)
Inj (Hum Emp)
Inj (Hum Prb)
Inj (Hum Pyr)
Inj (Pore Mc)
Inj (Hum Emp)
Inj (Hum Pyr)
Inj (Pore)
Sach
Susp
Susp
Pdrs
Tab
Tab
Pdrs
Tab
Pdr Sach
Pdrs
Pdrs
Pdrs
Tab
Tab
Tab
Tab
Pdrs
Susp
Susp
Oral Soln
Susp
Syr
Pdrs
Suppos
Syr
Syr
Tab E/C
Tab E/C
Tab E/C
Tab Solb
Tab
Tab
Tab Solb
Tab
Tab
Tab E/C
Skin Patch
Vag Ring
Val Tab
Skin Patch
Tab
Vag Ring
Implant
Tab
Tab
Tab Sublingual
Oint
Pdr
Gel
Top Soln
Oral Susp
Susp
Oral Susp
Susp
Oral Susp
Sach
Sach
Tab
Susp
Susp
Oral Soln Paed
Oral Susp
Syr
Oral Soln
Oral Soln Paed
Syr
Tab Chble
Syr
Susp

Tab E/C
Tab
Eye Irrig
Eye Lot
Eye Oint
Irrig
Nels
Nsl Douche
Nsl Dps
Nsl Mist
Nsl Soln
Soln
Soln Ster
Top Irrig
Top Irrig
Top Irrig
Pdrs
Sach
Tab
Pdrs
Sach
Tab Eff
Pdrs
Pdrs
Tab
Soln
Soln
Pdrs
Pdrs
Susp
Oral Susp
Susp
Syr
Oral Soln
Susp
Syr
Pdrs
Tab
Tab
Pdrs
Susp
Oral Susp
Susp
Oral Soln
Susp
Susp
Pdrs
Pdrs
Tab Chble
Pdrs
Tab Chble
Tab Eff
Pdrs
Tab
Tab Eff
Pdrs
Tab
Tab Chble
Tab
Tab
Tab Chble
Liq Spec
Tab
Pdrs
Tab Eff
Orodisper Tab
Rapid Tab
Tab
Tab Eff
Tab Eff Solb
Capl
Orodisper Tab
Rapid Tab
Tab Eff
Tab Eff Solb
Gran Eff Sach
Tab
Capl
Rapid Tab
Tab
Tab Eff
Tab Eff Solb
Oral Susp
Oral Soln

Tab Disper
Suppos
Tab E/C
Suppos
Tab
Tab Disper
Suppos
Oral Susp
Susp
Susp
Susp
Susp
Susp
Susp
Susp
Oral Susp
Susp
Oral Soln
Susp
Susp
Oral Susp
Oral Soln
Spy
Spy
Eye Dps
Soln
Eye Oint
Oint
Ear/Eye/Nsl Dps
Eye/Ear/Nose Dps
Nsl Dps
Eye Oint
Eye/Ear/Nose Dps
Nsl Dps
Eye/Ear Dps
Eye Dps
Eye Dps Viscous
Nsl Dps
Eye Lot
Eye Oint
I/V Inf
Irrig
Nels
Nsl Douche
Nsl Dps
Nsl Mist
Nsl Soln
Soln
Soln Ster
Nsl Dps
Nsl Dps
Eye Lot
Nsl Dps
Ster Soln
Ster Resp Soln
Eye Oint
Inh Soln
Nsl Dps
Soln
Ster Resp Soln
Ster Soln
Oint
Oromucosal Tab
Pdrs
Mthwsh
Oint
Mthwsh (Aniseed)
Mthwsh (Mint)
Paste
Mthwsh
Mthwsh (Aniseed)
Paste
Ear Dps
Lot
Scalp Applic
Shampoo
Crm
Face Wsh
Scalp Applic
Face Wsh
Shampoo
Soln
Oint
Scalp Lot

Scalp Lot
Oint
Oint
Scalp Applic
Lot
Scalp Applic
Oint
Scalp Applic
Lot
Oint
Oint
Lot
Scalp Applic
Scalp Applic
Oint
Oily Crm
Oint
Fatty Oint
Oint
Fatty Oint
Oily Crm
Lot
Oint
Gel
Lot
Lot
Oint
Scalp Lot
Scalp Lot
Eye Dps
Eye Oint
Lot
Oint
Eye Dps
Eye Oint
High Lipid Crm
Lot
Mthwsh
Oint
Eye Oint Lanolin Free
Lot
Oint
Oint
Ear Dps
Eye Dps
Eye Oint
Lot
Ear Dps
Eye Dps
Eye Oint
High Lipid Crm
Lot
Mthwsh
Eye Oint
Eye Oint Lanolin Free
Lot
Emuls
Oint
Scalp Lot
Emollient Crm
Emuls
Scalp Lot
Emollient Crm
Emuls
Oint
Emollient Crm
Oint
Scalp Lot
Oint
Ear Dps
Scalp Lot
Scalp Lot
Oint
Emuls
Ext Shampoo
Oint Strong
Paste
Soln
Oint Strong
Paste
Soln
Paste
Pomade
Oint

Paste
Pomade
Wax Sticks
Paste
Pomade
Paste
Pomade
Wax Sticks
Paste
Pomade
Wax Sticks
Oint
Paste
Pomade
Lot
Oint
Paste
Crm
Gel
Lot
Paste
Soln
Crm
Gel
Oint
Paste
Soln
Lot
Crm
Lot
Lot
Gel
Lot
Lactobi Lot
Lot
Lot
Soln
Crm
Gel
Soln
Oint
Paste
Crm
Gel
Oint
Paste
Soln
Emuls
Oint
Oint Strong
Paste
Soln
Lot
Soln
Soln
Nsl Oint
Oint
Eye Dps
Eye Dps Ud
Eye Oint
Oint
Gel
Crm
Top Soln
Vag Gel
Top Soln
Vag Gel
Soln
Soln
Gel
Eye Dps
Pdr
Pdr
Soln
Pdr
Gel
Oint
Soln
Lot
Lot
Soln
Eye Irrig
Eye Lot
Eye Oint

I/V Inf
Irrig
Nels
Nsl Douche
Nsl Dps
Nsl Mist
Nsl Soln
Soln Ster
Soln
Emollient/Crm
Oint
Soln
Skin Cleanser Soln
Antis Soln
Oint
Lot
Soln
Oint
Soln
Soln
Vac Inact (PDE)
Vac Inact (PDE)
Skin Patch
Soln
Mucilage
Skin Patch
Gel (S)
Oint
Soln Glacial
