# Supplementary material for: New mechanism to identify cost savings in English NHS prescribing: minimising ‘price per unit’, a cross-sectional study
Source: BMJ Open. 2018 Feb 8;8(2):e019643. doi: 10.1136/bmjopen-2017-019643 (PMC5829890; doi:10.1136/bmjopen-2017-019643)
Supplement: Supplementary file 2 [file bmjopen-2017-019643supp002.pdf]

Appendix B – BNF codes excluded from price-per-unit analysis

0302000C0____BE
0302000C0____BF
0302000C0____BH
0302000C0____BG
0904010H0%
0904010H0%
1311070S0____AA
1311020L0____BS
0301020S0____AA
190700000BBCJA0
0604011L0BGAAAH
1502010J0____BY
0107010S0AAAGAG
060106000BBAAA0
190201000AABJBJ
190201000AABKBK
190201000AABLBL
190201000AABMBM
190201000AABNBN
190202000AAADAD
